# Regioselective *para*‐Carboxylation of Catechols with a Prenylated Flavin Dependent Decarboxylase

**DOI:** 10.1002/anie.201708091

**Published:** 2017-10-02

**Authors:** Stefan E. Payer, Stephen A. Marshall, Natalie Bärland, Xiang Sheng, Tamara Reiter, Andela Dordic, Georg Steinkellner, Christiane Wuensch, Susann Kaltwasser, Karl Fisher, Stephen E. J. Rigby, Peter Macheroux, Janet Vonck, Karl Gruber, Kurt Faber, Fahmi Himo, David Leys, Tea Pavkov‐Keller, Silvia M. Glueck

**Affiliations:** ^1^ Department of Chemistry, Organic & Bioorganic Chemistry University of Graz, NAWI Graz, BioTechMed Graz Heinrichstrasse 28/2 8010 Graz Austria; ^2^ Manchester Institute of Biotechnology University of Manchester 131 Princess Street Manchester M1 7DN UK; ^3^ Max Planck Institute of Biophysics Max-von-Laue Strasse 3 60438 Frankfurt am Main Germany; ^4^ Department of Organic Chemistry Arrhenius Laboratory Stockholm University 10691 Stockholm Sweden; ^5^ Institute of Molecular Biosciences University of Graz, NAWI Graz, BioTechMed Graz Humboldtstrasse 50 8010 Graz Austria; ^6^ Institute of Biochemistry Graz University of Technology Petersgasse 12 8010 Graz Austria; ^7^ Austrian Centre of Industrial Biotechnology (ACIB) Austria

**Keywords:** biocatalysis, carboxylation, catechols, prenylated FMN

## Abstract

The utilization of CO_2_ as a carbon source for organic synthesis meets the urgent demand for more sustainability in the production of chemicals. Herein, we report on the enzyme‐catalyzed *para*‐carboxylation of catechols, employing 3,4‐dihydroxybenzoic acid decarboxylases (AroY) that belong to the UbiD enzyme family. Crystal structures and accompanying solution data confirmed that AroY utilizes the recently discovered prenylated FMN (prFMN) cofactor, and requires oxidative maturation to form the catalytically competent prFMN^iminium^ species. This study reports on the in vitro reconstitution and activation of a prFMN‐dependent enzyme that is capable of directly carboxylating aromatic catechol substrates under ambient conditions. A reaction mechanism for the reversible decarboxylation involving an intermediate with a single covalent bond between a quinoid adduct and cofactor is proposed, which is distinct from the mechanism of prFMN‐associated 1,3‐dipolar cycloadditions in related enzymes.

Carboxylation reactions have received considerable attention in view of the use of CO_2_ as an abundant C_1_ building block for sustainable chemical production.^1^ However, to date, only a few examples of CO_2_ fixation reactions have been realized on industrial scale, mainly owing to the high energy input required for substrate activation. In recent years, biocatalysts^2^ have been exploited as attractive alternatives to chemical methods^1^, ^3^ to catalyze carboxylation reactions under mild, aqueous conditions. Whereas the biocatalytic carboxylation of aldehydes (TPP‐dependent pyruvate decarboxylases),^4^ epoxides (epoxide carboxylases from *Xanthobacter* sp.),^5^ and heteroaromatic compounds, such as pyrroles (pyrrole‐2‐carboxylate decarboxylase from *Bacillus megaterium*)^6^ and indoles (indole‐3‐carboxylate decarboxylase from *Arthrobacter nicotianae*),2e exhibited narrow substrate specificity, promising results were obtained in the biocatalytic carboxylation of phenols and styrenes. *ortho*‐Benzoic acid decarboxylases and phenolic acid decarboxylases show a relaxed substrate specificity for the *ortho*‐carboxylation of phenols^7^ and the β‐carboxylation^8^ of styrenes, respectively, whilst maintaining their exquisite regioselectivity.

To expand the toolbox for biocatalytic carboxylation, we searched for enzymes enabling the regiocomplementary *para*‐carboxylation of phenols. Most of the already characterized enzymes either require an ATP‐consuming activation (phosphorylation) step prior to carboxylation (phenylphosphate carboxylases),^9^ suffer from a rapid loss of activity under aerobic conditions, especially after purification (4‐hydroxybenzoate^10^ and 3,4‐dihydroxybenzoate decarboxylases2d, 11) or have not been extensively examined in vitro yet,^12^ which limits their usability for biotransformations. Based on a literature survey and a preliminary activity screen of heterologously expressed potential *para*‐carboxylases (see the Supporting Information), 3,4‐dihydroxybenzoic acid decarboxylases from *Enterobacter cloacae* (*Ec*AroY) and *Klebsiella pneumoniae* (*Kp*AroY, 89 % identical) were selected for further studies.2d, 13 Both enzymes belong to the UbiD family and are related to a ferulic acid decarboxylase (Fdc1), which has been shown to facilitate the (de)carboxylation of cinnamic acids in the presence of a recently discovered prenylated flavin (prFMN) cofactor.^14^

Owing to the natural occurrence of the UbiD‐associated prenyltransferase UbiX in the *E. coli* expression host, lyophilized whole cells displayed high decarboxylation activity in initial screenings (see the Supporting Information). However, upon purification of the decarboxylase from the *E. coli* host, only little enzyme activity could be detected for either of the two enzymes, despite additional co‐expression with the UbiD‐associated prenyltransferase UbiX to provide sufficient prFMN in vivo. Upon in vitro reconstitution with reduced prFMN (Figure 1 a),14a, 15 decarboxylation activity could be detected with 3,4‐dihydroxybenzoic acid (3,4‐DHBA, **1**) following brief exposure to oxygen to generate the active prFMN^iminium^ form (Figure 1 b). The lack of activity for anaerobically reconstituted protein clearly demonstrates the requirement for oxidative maturation of the prFMN cofactor. EPR and UV/Vis spectroscopy revealed the presence of a radical semiquinone intermediate following in vitro reconstitution and oxidation, which is reminiscent of an intermediate observed with the related *E. coli* UbiD^15^ (see the Supporting Information). After reconstitution and maturation, the enzyme activity has a half‐life of only 5–8 min under aerobic conditions but remained unchanged for at least 14 h under anaerobic conditions (Figure 1 c).

**Figure 1:**
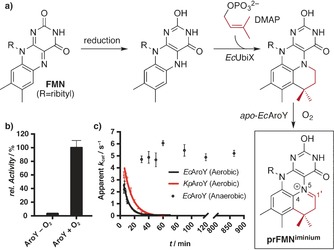
a) Established in vitro reconstitution of AroY with prFMN generated by UbiX from FMN and DMAP and subsequent aerial oxidation. b) AroY requires oxygen for activation; the activity was measured using 150 μm 3,4‐DHBA (**1**) and is relative to the highest activity detected. c) Decay of oxidized *Kp*AroY and *Ec*AroY when kept under aerobic or anaerobic conditions. Measured with 150 μm **1**. *t* ${}_{1/2}$ =8.1±1.3 min for *Kp*AroY and 5.3±1.2 min for *Ec*AroY.

A comparison of the *Kp*AroY and *Ec*AroY crystal structures revealed only small differences, in accordance with the similar activities observed in solution experiments. The AroY monomer consists of an N‐terminal prFMN‐binding domain (residues 1–339), an oligomerization domain (residues 340–475), and a C‐terminal α‐helix (residues 476–495; Figure 2 a). A comparison with the previously reported structures of the fungal Fdc1 and *E. coli* UbiD^14^ shows that AroY structures adopt an “open” conformation, where the position of the prFMN domain is more akin to that observed for UbiD, compared to the more closed conformation observed for Fdc1 (see the Supporting Information). This open conformation is observed for all crystallographically independent AroY monomers, and in the 4.6 Å cryo‐EM solution structure of *apo‐Ec*AroY (Figure 2 a).

**Figure 2:**
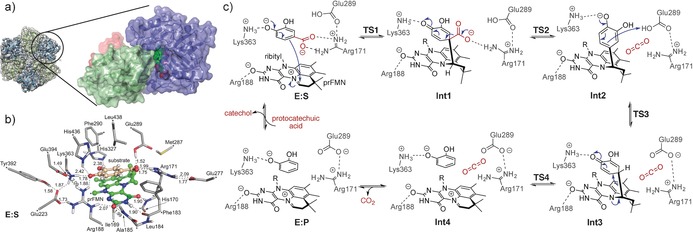
AroY structure and suggested mechanism based on calculations. a) Hexameric quaternary structure of *Ec*AroY. Dimer pairs are shown in ribbon representation in green and blue. The cryo‐EM envelope is shown as a gray translucent surface. A detailed view of the *Ec*AroY monomeric structure (in the circle) showing the prFMN‐binding domain (blue), oligomerization domain (green), and C‐terminal helix (red) is also given. b) Optimized structure of the active‐site model employed in the computational study. Atoms marked with asterisks were fixed during the geometry optimization. The prFMN cofactor is shown in green and the substrate in salmon ball‐and‐stick presentation. Distances are given in Å. For clarity, only polar hydrogen atoms and the hydrogen atoms on the substrate are shown. c) Reaction mechanism suggested on the basis of the calculations.

The active site of AroY is clearly defined by the prenylated isoalloxazine moiety of the cofactor and the presence of key conserved residues (Figure 2 b). It is situated near the hinge point of the prFMN‐binding domain motion, and at the cleft between the oligomerization and the prFMN‐binding domains. Two water molecules are clearly defined in the active site; one of them forms hydrogen bonds to His327 and Lys363 while the other one interacts with Lys363, His436, and Arg188. We hypothesize that the two water molecules mimic the two hydroxy groups at the *meta*‐ and *para*‐positions of the substrate protocatechuic acid (**1**). Given the relative rigidity of the substrate, we superimposed a catechol moiety onto the two water molecules, and the carboxylate moiety was positioned in close proximity to the prFMN iminium group. While this has some similarity to the structure of the Fdc1:substrate complex (PDB‐ID: 4ZA7),14a the exact relative position of the substrate carboxylate moiety and the prFMN N5−C1′ iminium linkage is different. In Fdc1, the substrate α‐carbon atom adjacent to the carboxyl group is located directly above the prFMN C1′ atom whereas in AroY, the α‐carbon atom is located above the isoalloxazine N5 atom (see the Supporting Information).

Exchanging several amino acids within the putative catechol‐binding motif (Arg188, His327, Lys363 to Ala and His436 to Lys or Thr) led to a complete loss of activity. The same effect was observed upon exchange of Arg181 or Glu289, which are located near the carboxylate group of the substrate, to Ala. The analogous Glu282 in Fdc1 is proposed to be required for the donation of a proton to the covalently bound intermediate,14a, 16 which hints at a similar role for Glu289 as a catalytic acid in AroY.

Based on the available structural, mutational, and kinetic data as well as DFT calculations (see the Supporting information), we propose a reaction mechanism involving a quinoid intermediate (Figure 2 c). This intermediate has chemical similarity to that proposed for the phenolic acid decarboxylases (PAD)8a and is different from the 1,3‐dipolar cycloaddition mechanism proposed for Fdc1.14a, 16, 17, 18 The calculations, employing a large model of the active site with 283 atoms (Figure 2 b), suggested that the generation of a cycloadduct is unlikely in the case of AroY as it would require the formation of a very strained intermediate (Figure S29). The latter is not required in the case of the cinnamic acid like substrates of Fdc1, for which previous calculations have validated the proposed 1,3‐dipolar cycloaddition mechanism to the exocyclic alkene.16b, 17b Instead, the mechanism involving a quinoid intermediate was calculated to have feasible energy barriers, which are significantly lower than those of the 1,3‐cycloaddition mechanism. The calculated energy profile and optimized structures of all intermediates and transition states along the reaction pathway are given in Figures S30–S32.

In view of synthetic applicability, the substrate scope of *Ec*AroY was investigated with different protocatechuic acid derivatives (Scheme 1 a, **1**–**3**). *Ec*AroY exhibited high decarboxylation activity with 3,4‐dihydroxybenzoic acid (**1**, >99 % after 1 h; Scheme 1 a) and gallic acid (**2**), which reacted at a slightly lower rate than **1** (ca. 83 % conv. after 1 h). For its isomer 2,3,4‐trihydroxybenzoic acid (**3**), however, no reaction was observed. Very similar kinetic parameters for the decarboxylation of **1** with purified and in vitro reconstituted enzymes were determined for both AroY enzymes (*Kp*AroY: *v*_max_^app^=4.7±0.4 s^−1^, *K*_m_^app^=96±23 μm; *Ec*AroY: *v*_max_^app^=4.6±0.4 s^−1^, *K*_m_^app^=61±17 μm). These parameters are reported as apparent values given the very low prevalence of inactive species (i.e., enzyme‐bound FMN or radical prFMN).

**Scheme 1:**
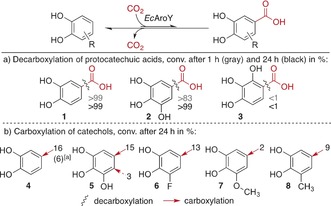
*Ec*AroY substrate screening with lyophilized *E. coli* whole cells containing the heterologously expressed decarboxylase. Potassium bicarbonate (3 m) was used as the CO_2_ source in the carboxylation assays. [a] Pressurized CO_2_ (30 bar) was used for the carboxylation.

To force the reaction equilibrium towards the carboxylation of suitable substrates, we subjected phenols **4**–**8** to a 3 m potassium bicarbonate buffer as a CO_2_ source (Scheme 1 b). Whereas the carboxylation of simple phenols was not successful (nonsubstrates are shown in Table S7), *Ec*AroY catalyzed the regioselective *para*‐carboxylation of catechol (**4**) in the presence of either bicarbonate or pressurized CO_2_ (30 bar). Apart from **4**, *Ec*AroY also accepted pyrogallol (**5**) as a substrate, and carboxylation with bicarbonate predominantly occurred in the *para*‐position to the central hydroxy group to give **2**. Carboxylation in vicinity to the peripheral hydroxy groups occurred only to a minor extent to give **3**.

As the catechol scaffold appears to be crucial for substrate acceptance, the carboxylation of substituted catechols **6**–**8** with bicarbonate was tested (Scheme 1 b). Small electron‐withdrawing (3‐F, **6**) to medium‐sized electron‐donating (3‐OMe, **7**; 3‐Me, **8**) substituents were tolerated in the 3‐position, and carboxylation occurred exclusively at the 5‐position, that is, in *para*‐position to the central hydroxy group, which was confirmed by NMR spectroscopy (see the Supporting Information).

In conclusion, our data contribute to a better understanding of the ATP‐independent *para*‐carboxylation of phenolic substrates. Crystal structures and in vitro reconstitution data unambiguously demonstrate that prFMN is employed as a cofactor, and that oxidative maturation is required for activity. The exact mechanism of oxidative maturation and the cause of the observed oxygen sensitivity remain unclear at this stage. Owing to the preference of AroY for catechols over simple phenols, a second hydroxy group seems to be mandatory for ideal substrate positioning (with a hydrogen bond between OH and His327; Figure 2 b) in the active site of the enzyme. The second hydroxy group further enhances the nucleophilicity of the (catechol) substrate to facilitate the nucleophilic addition step onto prFMN. Electron‐withdrawing and ‐donating groups are tolerated in the *meta*‐position relative to the carboxylation site whereas substitution in the *ortho*‐position was not tolerated owing to steric hindrance in the active site.

In the context of the wider UbiD family, the metal‐assisted binding and the associated oxidative maturation of prFMN are common to all biochemically and structurally characterized enzymes (the fungal Fdc1, *E. coli* UbiD, and AroY). The substrate binding specificity is distinct for each of these enzymes, but in all cases appears to be largely governed by residues derived from the oligomerization domain that are involved in binding to the non‐carboxylate substrate moiety. The carboxylate group, on the other hand, is bound near the conserved Glu‐Arg‐Glu/Asp triad of ionic residues, which is located near the prFMN N5=C1′ iminium moiety. A key difference is the relative position of the oligomerization and prFMN domains, and thus the relative position of the substrate‐binding and carboxylate‐binding motifs. These are considerably closer in the fungal Fdc1 structure than in bacterial UbiD and AroY. A putative domain motion might allow the UbiD/AroY enzymes to adopt a more Fdc1‐like conformation, but this has not been directly observed. While the quinoid‐based mechanism proposed for AroY suggests an alternative to 1,3‐dipolar cycloadditions in the case of aromatic substrates, it does not explain how the (de)carboxylation of non‐phenolic substrates^19^ is achieved. A better understanding of the UbiD enzyme family will require further studies of these and additional family members.^20^

## Conflict of interest

The authors declare no conflict of interest.

## Supporting information

As a service to our authors and readers, this journal provides supporting information supplied by the authors. Such materials are peer reviewed and may be re‐organized for online delivery, but are not copy‐edited or typeset. Technical support issues arising from supporting information (other than missing files) should be addressed to the authors.

Supplementary
