# Supplementary material for: Regioselective para‐Carboxylation of Catechols with a Prenylated Flavin Dependent Decarboxylase
Source: Angew Chem Int Ed Engl. 2017 Oct 2;56(44):13893–7. doi: 10.1002/anie.201708091 (PMC5656893; doi:10.1002/anie.201708091)
Supplement: Supplementary file 1 — Supplementary [file ANIE-56-13893-s001.pdf]

Angewandte Chemie
Eine Zeitschrift der Gesellschaft Deutscher Chemiker

Supporting Information

Regioselective *para*-Carboxylation of Catechols with a Prenylated Flavin Dependent Decarboxylase

Stefan E. Payer[+], Stephen A. Marshall[+], Natalie Bärland, Xiang Sheng, Tamara Reiter, Andela Dordic, Georg Steinkellner, Christiane Wuensch, Susann Kaltwasser, Karl Fisher, Stephen E. J. Rigby, Peter Macheroux, Janet Vonck, Karl Gruber, Kurt Faber, Fahmi Himo, David Leys, Tea Pavkov-Keller, and Silvia M. Glueck**

anie_201708091_sm_miscellaneous_information.pdf

Table of Contents

Table of Contents 1
Experimental Procedures 3
General information 3
Molecular Biology 3
Cloning, expression and purification of AroY 3
Site-directed mutagenesis 3
Isolation and cloning of the UbiX gene from Escherichia coli (E. coli) 5
Heterologous expression of other potential p-carboxylases 5
Characterization of AroY in conjugation with prFMN 5
Reconstitution with prFMN 5
Spectroscopic characterization 5
Photometric Activity assay 5
Binding of FMN 6
pH study with EcAroY 6
Deuterium exchange experiments 6
(De)carboxylation of with *Ec*AroY *E. coli* whole-cell preparations 6
Substrate scope experiments 6
Preparative-scale carboxylation of catechols **6–8** and isolation of 3,4-dihydroxybenzoic acid products 7
Activity of EcAroY mutants 7
Determination of conversion 7
Structural Biology 8
Protein crystallization and structure solution 8
Structure analysis and cavity calculations 10
Sample preparation and cryo-electron microscopy (EM) 10
Quantum chemical calculations 10
Construction of the active-site model 10
Density functional theory (DFT)-calculations 11
Results and Discussion 11
Molecular biology 11
Heterologous expression and purification of AroY (variants) 11
Sequencing result of ubiX amplification from E. coli 12
Preliminary Results 13
Enzyme screening 13
Characterization of AroY in conjugation with prFMN 14
Spectral properties of reconstituted AroY 14
Enzyme Kinetics 15
Binding of FMN 15
pH study with EcAroY 16
(De)carboxylation with AroY *E. coli* whole-cell preparations 16
Substrates not accepted by EcAroY 16
Activity of EcAroY variants 17

NMR for determining carboxylation regioselectivity 18
Deuterium exchange experiments 22
Structural Biology 24
Sequence alignment with other (de)carboxylases 24
Crystallization and structure analysis 25
Quantum Chemical Calculations 29
Cartesian coordinates 32
References 56
Author Contributions 56

Experimental Procedures

General information

All reagents and buffer components were acquired from commercial sources: "Tris Bufferan" (≥99%, Roth), sodium chloride (>99.8%, Roth), sodium dithionite (≥ 86%, Riedel deHaën), flavin mononucleotide sodium salt (76% riboflavin content, Sigma Aldrich), □,□-dimethylallylmonophosphate ammonium salt (DMAP; Sigma Aldrich), manganese chloride tetrahydrate (≥99%, Fluka), imidazole (Roth), sodium dihydrogenphosphate and disodiumphosphate (both Sigma Aldrich)
Substrates were all attained from commercial sources: 3,4-dihydroxybenzoic acid (**1**, >99%, Sigma Aldrich), gallic acid (**2**, 98%, Acros), 2,3,4-trihydroxybenzoic acid (**3**, 97%, Sigma Aldrich), catechol (**4**, ≥99%, Sigma Aldrich), pyrogallol (**5**, ≥98%, Fluka), 3-fluorocatechol (**6**, 99%, Alfa Aesar), 3-methoxycatechol (**7**, 99%, Sigma Aldrich), 4-methylcatechol (**8**, ≥95%, Sigma Aldrich). L-Ascorbic acid (≥ 99.5%, Sigma Aldrich) was used as antioxidant for catechol substrates.
Ampicillin sodium salt, kanamycin sulfate and Isopropylthiogalactoside (IPTG) were all purchased from Formedium. Bradford reagent for determining protein concentration was obtained from BIORAD Laboratories.
Anaerobic work was carried out within a Belle technology glovebox under nitrogen atmosphere. Buffers were rendered anaerobic by purging with N_2 for 2–3 hours. UV-Vis spectra were recorded on an Agilent Cary 60 UV-Vis photometer.
Samples from (de)carboxylation screenings were analyzed on an Agilent 1260 Infinity HPLC device equipped with a Phenomenex Luna reversed phase column (C18 [2], 100A, 250 × 4.6 mm × 5 µm) and a DAD detector at 25 °C using the following separation method: flow = 1 mL/min; mobile phase A: water + 0.1% v/v TFA, B: acetonitrile + 0.1 %v/v TFA; 0–2 min (100% A), 2–8 min (100–50% A), 8–11 min (50% A), 11–14 min (50–0% A), 14–16 min (0% A), 16–18 min (0–100% A), 18–21 min (100% A). Quantification of phenols and 4-hydroxybenzoic acid derivatives was performed at 270 nm after calibration (table S3).
Routine one- and two-dimensional ^{1}H and ^{13}C NMR experiments for determination of carboxylation regioselectivity were performed at 20 °C on either a Bruker Avance 300 or a Bruker BioSpin 700 NMR unit. Chemical shifts are given in ppm relative to the solvent (^{1}H: CD_3OD = 3.31 ppm, $(CH_3)_2CO$ = 2.05 ppm). H–D exchange experiments were measured on a Bruker 500 MHz NMR spectrometer.

Molecular Biology

Cloning, expression and purification of AroY

AroY genes were synthesized by GeneArt (ThermoScientific), codon optimized for expression in *E. coli* and amplified by PCR for insertion into pET28a using InFusion (Takara). Plasmids were transformed into BL21 (DE3) for expression. Cells were grown in terrific broth media at 37 °C at 180 rpm shaking until OD_{600nm} ~0.6, at which point the temperature was reduced to 18 °C and protein expression was induced with 0.2 mM IPTG. Cells were grown for ~18 hours after induction prior to harvesting by centrifugation at 6000 g.
Cells were resuspended in 50 mM Tris pH7.5, 300 mM NaCl (buffer A) with approximately 0.1 mg mL^{-1} DNase and RNase (Sigma) added. A protease inhibitor cocktail tablet (Sigma) was also added prior to cell lysis by constant cell disruptor at 20 kPsi. Lysate was centrifuged at 140 000 g for 1 hour; the supernatant was applied to gravity flow NiNTA resin (Qiagen) prior to washing with 4 column volumes of buffer A containing 10 mM imidazole, followed by the same volume of 40 mM imidazole in buffer A. Protein was eluted from the column using buffer A containing 250 mM imidazole, prior to being desalted into 20 mM Tris pH 7.5, 200 mM NaCl (buffer B). All further experiments were carried out in buffer B unless stated otherwise. Protein concentrations were estimated by the use of a predicted ε = 31400 M^{-1} cm^{-1}, as calculated by ExPasy Protparam. Both homologues have the same extinction coefficient.

Site-directed mutagenesis

Variants of *Ec*AroY were produced using a QuikChange II Site-Directed Mutagenesis Kit (Agilent) with the respective primers listed in Table S1 (ordered from Eurofins). Primers were designed and analyzed online using the QuikChange Primer Design

tool (Agilent) and the OligoAnalyzer 3.1 tool (eu.idtdna.com), respectively. All mutants were expressed as soluble proteins with the same cultivation procedure as for the wild-type enzyme and proper insertion of the mutation was verified by gene sequencing (LGC genomics).

Table S1. Primer sequences for site-directed mutagenesis.

EcAroY variant	Primer sequence[a]
R40A	5'–CA GAA CTG GCA GGC GTT TAT GCT CAT ATT GGT GC–3'<br>5'–GC ACC AAT ATG AGC ATA AAC GCC TGC CAG TTC TG–3'
R171A	5'–GC CTG ACC GAT GTT ACC ATT CATGCT CTG TGT GTT CAA G–3'<br>5'–C TTG AAC ACA CAG AGC ATG AAT GGT AAC ATC GGT CAG GC–3'
N397A	5'–GCA ACC TAT AGC GAA CTG AAA GCC ATT ATT CTG GTG GAT GAG G–3'<br>5'–C CTC ATC CAC CAG AAT AAT GGC TTT CAG TTC GCT ATA GGT TGC–3'
N298A	5'–GGT TAT TGT GGT GAA GCA GCT CCG AGC CTG CCG–3'<br>5'–CGG CAG GCT CGG AGC TGC TTC ACC ACA ATA ACC–3'
C221A	5'–GC AAT CTA TAT TGG GGC AGCT TTT GAA GCA CCG ACA ACC–3'<br>5'–GGT TGT CGG TGC TTC AAA AGC TGC CCC AAT ATA GAT TGC–3'
Y392F	5'–CTG ATT GCA CTG GCA ACC TTT AGC GAA CTG AAA AAC–3'<br>5'–GTT TTT CAG TTC GCT AAA GGT TGC CAG TGC AAT CAG–3'
E394A	5'–CTG GCA ACC TAT AGT GCC CTG AAA AAC ATT ATT CTG GTG G–3'<br>5'–C CAC CAG AAT AAT GTT TTT CAG GGC ACT ATA GGT TGC CAG–3'
R188A	5'–CTG GCA GCA GGT GCT CAT ATT GAA GTG TTT CG–3'<br>5'–CG AAA CAC TTC AAT ATG AGC ACC TGC TGC CAG–3'
H189A	5'–CTG GCA GCA GGT CGT GCT ATT GAA GTG TTT CGT AAA AAA G–3'<br>5'–C TTT TTT ACG AAA CAC TTC AAT AGC ACG ACC TGC TGC CAG–3'
H436T	5'–CA CTG CCA GGT ATT CGT GGT ACA CAG TTA GAT CCG AGC CAG AG–3'<br>5'–CT CTG GCT CGG ATC TAA CTG TGT ACC ACG AAT ACC TGG CAG TG–3'
H436K	5'–CA CTG CCA GGT ATT CGT GGT AAA CAG TTA GAT CCG AGC CAG AG–3'<br>5'–CT CTG GCT CGG ATC TAA CTG TTT ACC ACG AAT ACC TGG CAG TG–3'
H327A	5'–G GTT GGT CCG TGT GAA GAA GCT ACC ACC CTG GC–3'<br>5'–GC CAG GGT GGT AGC TTC TTC ACA CGG ACC AAC C–3'
E223A	5'–C TAT ATT GGC GTA TGT TTT GCA GCA CCG ACA ACC C–3'<br>5'–G GGT TGT CGG TGC TGC AAA ACA TAC GCC AAT ATA G–3'
E289V	5'–C GGT CAT GCA ATG CCG GTA TTT CCG GGT TAT TGT G–3'<br>5'–C ACA ATA ACC CGG AAA TAC CGG CAT TGC ATG ACC G–3'
K363A	5'–CAT ACC ACA GGC GGT GGT GCG TTT CTG GGT ATC CTG–3'<br>5'–CAG GAT ACC CAG AAA CGC ACC ACC GCC TGT GGT ATG–3'
K363V	5'–CAT ACC ACA GGC GGT GGT GTG TTT CTG GGT ATC CTG–3'<br>5'–CAG GAT ACC CAG AAA CAC ACC ACC GCC TGT GGT ATG–3'
F138A	5'–GAT GAA CTG AGC ATG GCG CTG GCA GCA GGT CG–3'<br>5'–CG ACC TGC TGC CAG CGC CAT GCT CAG TTC ATC–3'
F138V	5'–GAT GAA CTG AGC ATG GTG CTG GCA GCA GGT CGC–3'<br>5'–GCG ACC TGC TGC CAG CAC CAT GCT CAG TTC ATC–3'
L438A	5'–CGT GGT CAT CAG GCG GAC CCG AGC CAG AGT CC–3'<br>5'–GG ACT CTG GCT CGG GTC CGC CTG ATG ACC ACG–3'

[a] Upper and lower sequences refer to forward- and reverse primers, respectively. Underlined codons indicate site of mutation.

Isolation and cloning of the UbiX gene from Escherichia coli (E. coli)

The gene encoding for UbiX was amplified from *E. coli* Neb5α genomic DNA (Invitrogen) by polymerase chain reaction (PCR) using the primers in table S2 (Eurofins) containing the restriction site for NdeI (forward primer) for the introduction of a *C*-terminal His_6Tag after cloning into pET21a(+) vector. pET21a(+) and *ubiX* were ligated as follows: T4-Ligase buffer 10× (2 µL, Thermo Scientific), water (12 µL), T4 Ligase (1 µL, Thermo Scientific), pET21a(+) vector (1 µL) were mixed with the digested insert-DNA (4 µL) and kept at 21 °C for 30 min followed by 4 °C for 4.5 h. The ligated plasmids were subsequently transformed into *E. coli* Neb5α, plated on LB-agar plates containing ampicillin (100 µg/mL) as the selection antibiotic and incubated at 30 °C for 16 h. >40 colonies could be detected after incubation. Proper constructs were verified in nine out of ten clones by control restriction digest and sequencing of plasmids isolated from an overnight culture obtained from five separate colonies.
Two clones of the *ubiX*::pET21a(+) construct encoding for UbiX with a *C*-terminal His_6Tag were transformed into *E. coli* BL21(DE3) and plated on a LB-agar plate containing ampicillin (100 µg/mL) as selection antibiotic and incubated at 30 °C for 16 h.

Table S2. Primer sequences for amplification of the UbiX gene from *E. coli* genomic DNA.

	Primer sequence
Forward primer NdeI	5'–CA GAA CTG GCA GGC GTT TAT GCT CAT ATT GGT GC–3'
Reverse primer	5'–GC ACC AAT ATG AGC ATA AAC GCC TGC CAG TTC TG–3'

Heterologous expression of other potential p-carboxylases

The genes encoding for the 3,4-dihydroxybenzoic acid decarboxylase from *Commensalibacter intestine* (3,4-DHBD_*Ci*), the carboxylyase-related protein from *Desulfotomaculum gibsoniae* (carboxylase_*Dg*), the 4-hydroxybenzoic acid decarboxylase from *Enterobacter cloacae* (4-HDB_*Ec*) and *Clamydophilia pneumoniae* (4-HBD_*Cp*) were synthesized at geneart (Germany, Regensburg) and subcloned in a common pET28a(+) vector (invitrogen) using the restriction sites of NdeI and XhoI. For protein overexpression the plasmids were transformed into *E. coli* BL21(DE3) host cells. In order to verify successful heterologous expression and to assess the protein's solubility, the cells were disrupted using ultrasonication and the separated supernatant and remaining cell pellet were analyzed by SDS-PAGE. For 4-HBD_*Ec*, 4-HBD_*Cp* and carboxylase_*Dg* soluble expression was obtained when using IPTG (0.5mM) for induction and otherwise similar conditions as for *Ec*AroY. Soluble expression of 3,4-DHBD_*Ci* was achieved by expression in chaperone cells '1' (dnaK-dnaJ-grpE groES-groEL) and '3' (dnaK-dnaJ-grpE).

Characterization of AroY in conjugation with prFMN

Reconstitution with prFMN

AroY enzymes were reconstituted with prFMN produced *in vitro* as previously described.[1] AroY enzymes were desalted into buffer B for crystallization and further experiments.

Spectroscopic characterization

UV-Vis spectra were taken immediately after exposure to oxygen, and samples were frozen for EPR spectroscopy. EPR spectra were obtained at X-band (~9.4 GHZ) using a Bruker ELEXSYS E500/580 spectrometer equipped with a Bruker Super High Q resonator and an Oxford Instruments ESR900 helium flow cryostat. Results are shown in Figures S5 and S6.

Photometric Activity assay

Activity was measured by following the decrease in protocatechuic acid (**1**) by the decrease in absorbance at 250 nm in a Cary Win UV 60 spectrophotometer (Figure S7 a). Substrate was dissolved in 50 mM KPi and was adjusted to pH 7 (See Figure S9 for pH optimization). AroY was reconstituted and stored anaerobically prior to being oxidized and measurements

taken with 1 µM enzyme. A range of protocatechuic acid concentrations was used for measurements. Any measurements of activity taken with substrate over 200 µM were done in a 2 mm pathlength cuvette due to absorption outside the linear range. Measurements were taken between 2 and 5 minutes of exposure to oxygen, to ensure decay in activity had minimal effect. All measurements were taken in triplicate, and error bars are shown as SEM (Figure S7 b).
The dependence on oxygen for activity was measured by the addition of 1 µM enzyme to 150 µM protocatechuic acid in a 1 cm pathlength cuvette. Where protein was oxidized prior to addition to substrate, 20 µL protein was removed from the anaerobic environment and was briefly exposed to atmospheric oxygen. The protein was then returned to the anaerobic glovebox for the activity to be measured. Combinations of aerobic and anaerobic substrate and oxidized and reduced protein were made to ascertain the dependence of oxidation, but not prolonged oxygen exposure, for activity. The decay of activity in an aerobic environment was measured by addition of 1 µM enzyme to 150 µM protocatechiuc acid. Three independent timecourse measurements were taken for each homologue and averaged. every 10 minutes, starting 10 minutes after initial oxidation. The anaerobic decay was measured after oxidation of reconstituted *Ec*AroY. The protein was removed from the anaerobic glovebox and was exposed briefly to atmospheric oxygen prior to being reintroduced to anaerobic conditions. The activity was measured every 30 minutes and after a period of 14.5 hours by the addition of 1 µM enzyme to 150 µM protocatechuic acid. Activity and decay experiments were performed using the same batch of reconstituted protein.

Binding of FMN

Both AroY homologues were adjusted to 6 µM. An Edinburgh Instrument FLS920 spectrofluorometer was used to measure the emission spectra between 300–420 nm, after excitation at 295 nm. 3 mM $MnCl_2$ and KCl were added to 1 mL enzyme in a quartz cuvette. FMN was added in 1 µL titrations. The relative difference of the peak at 333 nm and 340 nm for *Ec*AroY and *Kp*AroY respectively were used (Figure S8).

pH study with EcAroY

Initial rates of decarboxylation of **1** (150 µM) dissolved in SPG buffer (100 mM, succinic acid/sodium dihydrogen phosphate/glycine 2:7:7 as described by Qiagen) at different pH. Decarboxylation was measured by the absorbance decrease at 250 nm, using 2 µM enzyme in aerobic conditions.

Deuterium exchange experiments

Catechol (**4**), pyrogallol (**5**), 3-methylcatechol (**8**) and 3-fluorocatechol (**6**) were dissolved in KPi buffer (200 mM, pH 7.2) in D_2O, to a concentration of 10 mM. Reconstituted *Ec*AroY was added to a final concentration of 5 µM, and was left incubating with substrate for 24 hours at room temperature under anaerobic conditions. Spectra were measured using Bruker 500 MHz NMR spectrometer. Controls without enzyme, but with buffer B added were used to determine substrate spectra (Figure S21).

(De)carboxylation of with *Ec*AroY *E. coli* whole-cell preparations

Substrate scope experiments

Lyophilized *E. coli* whole cells containing the heterologously expressed *Ec*AroY wild type enzyme (30 mg/mL), ascorbic acid (antioxidant, 10 mM) and potassium bicarbonate (300 mg/mL) at pH 8.5 were applied for the carboxylation of substrates **4**–**8** (10 mM, supplied as aliquot of a 100 mM solution in acetone). Carboxylation of **4** (10 mM, supplied as aliquot of a 600 mM solution in MeOH) with *Ec*AroY (30 mg/mL) and pressurized CO_2 (30 bar) was done in TRIS-HCl buffer (100 mM, pH 9.0) in a HEL pressure reactor. For the decarboxylation of **1**–**3** (10 mM), lyophilized *Ec*AroY (20 mg/mL) and ascorbic acid (10 mM) in potassium phosphate buffer (50 mM, pH 7.0) were used. It is to note that *E. coli* whole-cell preparations of *Ec*AroY did not contain an extra plasmid encoding for UbiX since the prFMN producing UbiX was available from the expression host. Product formation was measured with HPLC after 1 to 24 h and samples were all performed in duplicate (Table S7 summarizes non-substrates).

*Preparative-scale carboxylation of catechols **6–8** and isolation of 3,4-dihydroxybenzoic acid products*

Catechols bearing a –F, –Me or –OMe substituent *ortho* to one of the phenolic hydroxy groups (**6**–**8**) were carboxylated in a semi-preparative scale (20 mg).
Representative procedure for 3-fluorocatechol (**6**) as substrate: potassium bicarbonate (3 M, 4.68 g) was provided in a 25 mL crimp cap vial (Agilent); lyophilized *E. coli* whole cells containing the heterologously expressed *Ec*AroY (468 mg, 20 mg/mL) were rehydrated at 30 °C and 120 rpm shaking for 30 min in sodium phosphate buffer (100 mM, pH 5.5, 12.5 mL). **6** (20 mg, dissolved in 1.55 mL acetone) and ascorbic acid (27.5 mg, 1 eq, dissolved in 1.55 mL sodium phosphate buffer) were added to the cell suspension and this mixture was in turn transferred to the vial containing $KHCO_3$. The vial was sealed with a crimp cap and a septum (3 mm thickness) and was incubated in the darkness at 30 °C and 450 rpm shaking inside a benchtop incubator (VWR). After 41 h, CO_2 pressure was released and the reaction mixture was acidified (pH<4) by addition of HCl (6 M, 7–8 mL) (gas evolution!). The suspension was extracted with ethyl acetate (3 × 15 mL) with intermittent centrifugation for phase separation (4000 rpm). The dried (Na_2SO_4) and evaporated crude product was purified by column chromatography on silica gel 60 (Merck) using petrolether/ethylacetate 75:20 as solvent. When unconverted **6** (R_f = 0.56) was completely eluted, acetic acid (5% v/v) was added to the solvent to elute the product acid (R_f = 0.22). After evaporation, a red-brown solid residue (2.9 mg, 10.8% yield) was obtained. ^{1}H NMR (300 MHz, CD_3OD) δ [ppm] 7.28 (s, 1H), 7.23 (d, 1H, $J_{H–F}$ = 11.4 Hz). ^{13}C NMR (175 MHz, $(CD_3)_2CO$) δ [ppm] 165.9, 151.2 (d, C–F), 146.7 (d), 138.1 (d), 121.2 (d), 112.8, 108.8 (d). ^{19}F NMR (300 MHz, CD_3OD) δ [ppm] –138.8 (d, $J_{H–F}$ = 11.4 Hz). HR-MS (APCI–) *m/z*=171.009903 ($MH^–$) [calcd. 171.009903 ($MH^–$)].
2-Methyl-3,4-dihydroxybenzoic acid:
Grey-brown solid (5.8 mg, 21.4% yield). ^{1}H NMR (300 MHz, CD_3OD) δ [ppm] 7.32 (m, 2H), 2.21 (s, 3H). HR-MS (APCI–) *m/z*=167.034889 ($MH^–$) [calcd. 167.034982 ($MH^–$)].
2-Methoxy-3,4-dihydroxybenzoic acid:
Dark-brown oily residue (2.6 mg, 10% yield). ^{1}H NMR (300 MHz, CD_3OD) δ [ppm] 7.18 (s, 2 H), 3.87 (s, 3H); matches literature.[2] HR-MS (APCI–) *m/z*=183.030094 ($MH^–$) [calcd. 183.029897 ($MH^–$)].

Activity of EcAroY mutants

Decarboxylation of **1** and carboxylation of **4** using *Ec*AroY mutants (Table S1) was done by following the protocol for the substrate scope experiments. (Figure S10)

Determination of conversion

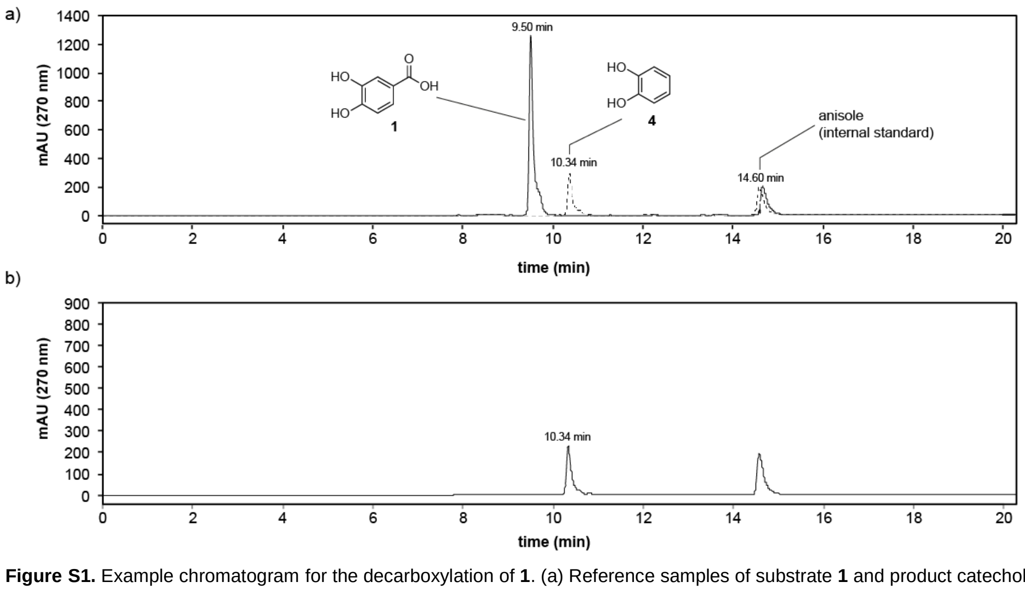

Figure S1. Example chromatogram for the decarboxylation of **1**. (a) Reference samples of substrate **1** and product catechol (**4**) (with anisole as external standard) (b) reaction sample of the aerobic decarboxylation of **1** with reconstituted *Ec*AroY^{prFMN}.

Table S3. Calibration data for accepted phenolic- and 4-hydroxybenzoic acid derivatives. For separation methods and system preferences refer to section 1: "General Information".

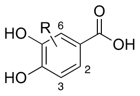

1–3

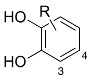

4–8

Compound	R	t_R (min)	k (mM^{-1})[a]	d[a]	R^2	Calibration range (mM)
**1**	H	8.9	1607	219	0.99857	0.5 – 10
**2**	3-OH	8.4	2605.6	11.4	1.00000	0.1 – 2
**3**	6-OH	9.5	3038.5	–9.31	0.99997	0.1 – 2
**4**	H	10.1	526.1	6.83	0.99994	0.5 – 10
**5**	3-OH	8.7	212.7	–3.83	0.99945	0.1 – 2
**6**	3-F	10.8	274.9	–0.70	0.999971	0.1 – 2
**7**	3-OMe	10.4	172.9	1.91	0.99961	0.1 – 1
**8**	3-Me	11.7	413.4	11.6	0.99997	0.1 – 2

[a] Calibration curve function: A (mAU at 270 nm) = k·c (mM) + d.

Structural Biology

Protein crystallization and structure solution

Screening for *Ec*AroY crystallization conditions was performed with an Oryx8 robot (Douglas Instruments) by sitting drop vapor-diffusion method in 96-well plates. Commercially available Index HT, XS Cryo, Salt RX (Hampton Research), JCSG+, Morpheus, PGA and MIDAS (Molecular Dimensions) screens were setup for initial screening. All crystallization plates were incubated at 289 K. Initial crystals of *apo-Ec*AroY were obtained in several crystallization conditions. The best diffracting native crystals of *apo-Ec*AroY were obtained in PGA screen #8 (0.2 M L-Arginine, 0.1 M sodium acetate pH 5.0, 8 % w/v γ-PGA). The diffraction quality crystals of *Ec*AroY SeMet-variant crystals were obtained in the optimized PGA condition #8 (0.15 M L-Arginine, 0.075 M sodium acetate pH 5.0, 6 % w/v γ-PGA). Crystallization drops of 1µL were pipetted with a 1:1 ratio of protein (14.7–5 mg mL^{-1} in 20 mM Tris/HCl pH 7.5, 0–200 mM NaCl) and screening solution. Yellow crystals of *Ec*AroY diffracting to 3.1 Å were grown in Morpheus screen condition #7 [0.06 M divalents (magnesium chloride hexahydrate; calcium chloride dehydrate), 0.1 M buffer system 2 (sodium HEPES; MOPS-acid, pH 7.5) and 50% v/v Precipitant Mix 3 (40% v/v glycerol; 20% w/v PEG 4000)] in the presence of FMN (2 mM) and $MgCl_2$ (10 mM). The structure of *Ec*AroY in complex with FMN was solved, and the soaking with catechol (**4**) and 3,4-DHBA (**1**) performed. Data to 3.5 Å could be collected revealing an additional electron density parallel to the isoalloxazine ring. Due to limited resolution and quality of this structure (and electron density), as well as the achievement to obtain *Ec*AroY crystals in complex with the real prFMN cofactor diffracting to 2.1 Å, we continued the analysis with the prFMN-*Ec*AroY structure.
After in vitro reconstitution, crystals of *holo-Ec*AroY were grown in condition A11 of the Morpheus (Molecular Dimensions). Sitting drops were made with 1:1 v/v protein precipitant ratio and a protein concentration of 7 mg mL^{-1}. Crystals of *Kp*AroY grew readily in sitting drops of Morpheus II (Molecular Dimension) condition H3, with a 1:1 v/v ratio of precipitant to protein and a protein concentration of 15 mg mL^{-1}. Diffraction quality crystals for *Kp*AroY were obtained in a $P2_1$ form (hexamer in the AU), while *holo-Ec*AroY crystallized in H 3 2 (a dimer in the AU).
Data were collected at the synchrotron beamlines P11 (DESY, PETRA III, Hamburg, Germany),[3] i04 (Diamond, UK), XRD1 (Elettra, Trieste, Italy) and ID23-1, ID23-2, ID29, ID30A-3, ID30B (ESRF, Grenoble, France).[4] Data were processed with the XDS[5] or with xia2 utilizing XDS. The structure of apo EcAroY was solved by SeMet-SAD using the program AutoSol in Phenix.[6] The initial model building was performed with AutoBuild[7] and manually within Coot.[8] For all other structures, molecular replacement was performed with Phaser[9] using single subunits of *Ec*AroY or UbiD (PDB-entry: 5M1C), for *Kp*AroY, as search templates. All models were refined using the Phenix suite of programs[6] and model rebuilding was performed in Coot.[8] Randomly chosen 5% of the reflections were not used in the refinement, but were set aside for R_{free} calculations.[10] Structure validation was performed using MolProbity.[11] Data collection and refinement statistics are

summarized in table S4. The X-ray structures and atomic coordinates have been deposited in the Protein Data Bank under the PDB-codes 5NY5, 5O3N, 5O3M. The cryo-EM map has been deposited in the EMDB with the accession code EMD-3718.

Table S4. Data collection and refinement statistics.

	*apo-Ec*AroY (5NY5)	*holo-Ec*AroY (5O3N)	*Kp*AroY (5O3M)
**Wavelength**	0.9796	0.9795	0.9200
**Resolution range**	48.06 – 2.50 (2.59 – 2.50)	30.07 - 2.05 (2.09 – 2.05)	38.08-2.23 (2.27-2.23)
**Space group**	H 3 2	H 3 2	P 2_1
**Unit cell (Å, °)**	209.9, 209.9, 162.2 90, 90, 120	208.3, 208.3, 157.9 90, 90, 120	81.9, 209.7, 116.0 90, 107.6, 90
**Total reflections**	960015 (92028)	418290 (19227)	621329 (62265)
**Unique reflections**	47197 (4613)	81410 (7073)	180400 (17981)
**Multiplicity**	20.3 (19.9)	5.1 (4.7)	3.4 (3.5)
**Completeness (%)**	100 (99)	99.4 (99.9)	99.8 (99.9)
**Mean I/σ(I)**	22.3 (3.3)	15.2 (1.2)	8.2 (1.3)
**Wilson B-factor**	55.04	42.39	36.17
**R_{-merge}**	0.118 (0.779)	0.061 (1.221)	0.115 (0.8952)
**R_{-meas}**	0.121 (0.799)	0.068 (1.327)	0.136 (1.061)
**CC1/2**	0.999 (0.893)	0.999 (0.588)	0.993 (0.534)
**CC***	1 (0.971)	1 (0.860)	0.998 (0.85)
**Reflections used in refinement / for R_{free}**	47195 (4613) 2360 (230)	81399 (8113) 4126 (400)	180311 (17975) 8846 (894)
**R_{-work}**	0.203 (0.310)	0.1733 (0.2853)	0.1627 (0.2673)
**R_{-free}**	0.234 (0.364)	0.1962 (0.2988)	0.2082 (0.3319)
**$CC_{(work)}$**	0.952 (0.794)	0.970 (0.813)	0.972 (0.824)
**$CC_{(free)}$**	0.938 (0.742)	0.962 (0.808)	0.953 (0.729)
**Number of non-H atoms**	7691	7934	23577
**macromolecules**	7432	7469	22261
**ligands**	24	112	70
**Protein residues**	977	976	2942
**$RMS_{(bonds)}$**	0.003	0.004	0.008
**$RMS_{(angles)}$**	0.57	0.74	0.90
**Ramachandran favored (%)**	96.6	97.33	96.21
**Ramachandran allowed (%)**	3.4	2.67	3.65
**Rotamer outliers (%)**	1.05	0.5	0.98
**Clashscore**	4.83	6.07	6.36
**Average B-factor**	56.30	58.19	50.03

**macromolecules**	56.73	58.45	50.44
**ligands**	57.01	66.28	46.27
**solvent**	42.63	50.08	42.76
**Number of TLS groups**	10	10	12

Statistics for the highest-resolution shell are shown in parentheses.

Structure analysis and cavity calculations

Sequence alignment was performed with T-coffee[12] and graphically rendered using ESPript 3.0.[13] The Dali server[14] and PDBeFold service at European Bioinformatics Institute (http://www.ebi.ac.uk/msd-srv/ssm)[15] were used to identify similar protein structures in the PDB. Protein interfaces were analyzed with the PDBePISA webserver (http://www.ebi.ac.uk/pdbe/prot_int/pistart.html).[16] Superposition of structures was performed with SSM Superposition,[15] as implemented in the program Coot. All structure-related pictures were generated using PyMOL (DeLano, W. L. (2002). *PyMOL*, http://www.pymol.org).

Sample preparation and cryo-electron microscopy (EM)

3 µL of a 0.15 mg/mL apo-*Ec*AroY sample was applied to freshly glow discharged Quantifoil R2/2 holey carbon grids (Quantifoil Micro Tools, Jena, Germany) that had been pretreated in chloroform for 2 hrs. The grids were blotted for 11 s in an FEI Vitrobot plunge-freezer at 10 ºC and 70 % humidity and plunge-frozen in liquid ethane. Cryo-EM images were collected on a JEOL 3200 FSC operating at 300 kV on a Gatan K2 direct electron detector as described previously[17] at a nominal magnification of 30,000× with a calibrated specimen pixel size of 1.12 Å, in a defocus range of 1–3 µm. The images were recorded using movie mode with 40 0.2 s frames using an electron dose of 1.35 e^-/Å^2/frame, resulting in a total exposure of 54 e^-/Å^2. The 40 frames of each 8 s movie were aligned using MotionCor2.[18] The first frame was discarded and the others averaged for processing. 43966 particles were picked using RELION 1.4,[19] and the contrast transfer function of every image was determined using gctf.[20] 2D and 3D classification was performed in RELION 1.4. 41157 particles were selected after 2D classification, and 22650 particles were selected from the best class after the subsequent 3D classification without applied symmetry. This data set of 22650 particles was refined using the gold standard refinement procedure of RELION 1.4, initially without applied symmetry and later with the clearly visible D3 symmetry applied. The particle polishing procedure in RELION 1.4 was applied to correct particle ensemble movements and to apply a resolution-dependent frame weighting.[21] 20 frames with an accumulated dose of 27 e^-/Å^2 were used for this procedure. The post-processing procedure implemented in RELION was applied to the final maps for B-factor sharpening and resolution validation.[22] The final map had a resolution of 4.6 Å. The X-ray structure was docked into the EM map in Chimera.[23]

Quantum chemical calculations

Construction of the active-site model

A model of the active site was designed on the basis of an earlier lower-resolution crystal structure of *Ec*AroY in complex with FMN and catechol, which showed no significant difference to the later determined 2.1 Å structure of *Ec*AroY with prFMN. The FMN was modified to the prFMN and the catechol was replaced by the protocatechuic acid substrate. As shown in Figure S2, the model consists of the substrate, the prFMN cofactor, residues that are suggested to be the general acid/base groups in the reaction (Glu289 and Lys363), residues that potentially form hydrogen bonds with the substrate and/or cofactor (His170, Arg171, Phe183, Leu184, Ala185, Arg188, His327 and His436), and other residues that contribute to the active site pocket (Ile169, Glu223, Glu277, Met287, Phe290, Tyr392, Glu394 and Leu438). The amino acids and the prFMN were truncated as shown in the figures of optimized structures, and hydrogen atoms were added manually. To avoid unrealistic movements of the groups during the geometry optimizations, certain atoms were kept fixed to their crystallographic positions as indicated in the figures of optimized structures. The model consists of 283 atoms and has a total charge of –2.

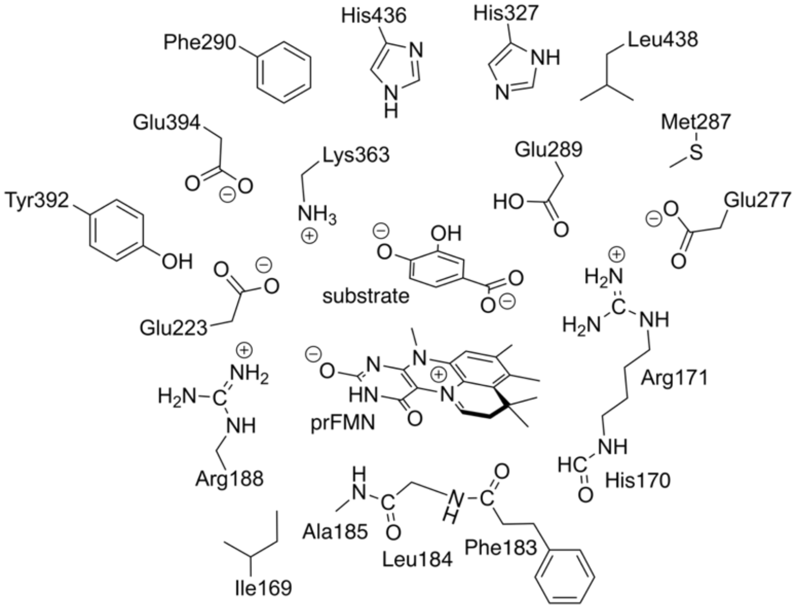

Figure S2. Schematic illustration of the active site model used in this study.

Density functional theory (DFT)-calculations

All the calculations presented here were carried out using the Gaussian 09 program[24] with the B3LYP hybrid density functional method.[25] Geometries were optimized with the 6-31G(d,p) basis set and more accurate energies were obtained by single-point calculations on the optimized structures with the larger basis set 6-311+G(2d,2p). The SMD solvation model was used to consider the effects of the rest of enzyme that was not included in the model.[26] Single-point energies at the same level of theory as the geometry optimization were calculated using $\varepsilon = 4$. Frequency calculations were preformed with the 6-31G(d,p) basis set to obtain zero-point energies (ZPE). For all the calculations, dispersion corrections were included using the DFT-D3(BJ) method.[27] The energies presented in this paper are thus the large basis set energies (which include dispersion) corrected for ZPE and solvation effects. Similarly to previous studies on other decarboxylases,[28] the entropy gain associated with the release of CO_2 gas was estimated to be equal to the translational entropy of the free molecule, calculated to be 11.1 kcal/mol at room temperature. This value is added to the corresponding step.

Results and Discussion

Molecular biology

Heterologous expression and purification of AroY (variants)

a)

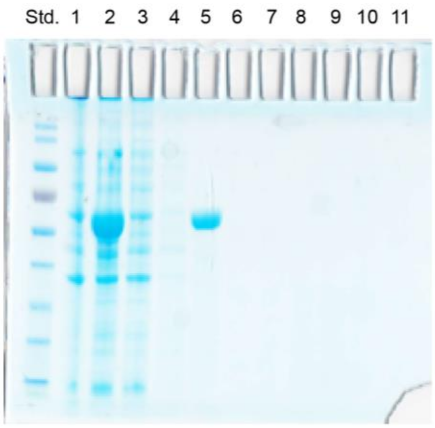

Lane	Sample
S	protein standard (PAGE Ruler, Thermo Scientific)
1	Pellet after cell disruption
2	Cell-free extract
3	HisTrap-colum flow-through
4	Pooled wash-fractions
5	pooled eluted protein practions
6 – 10	Empty

b)

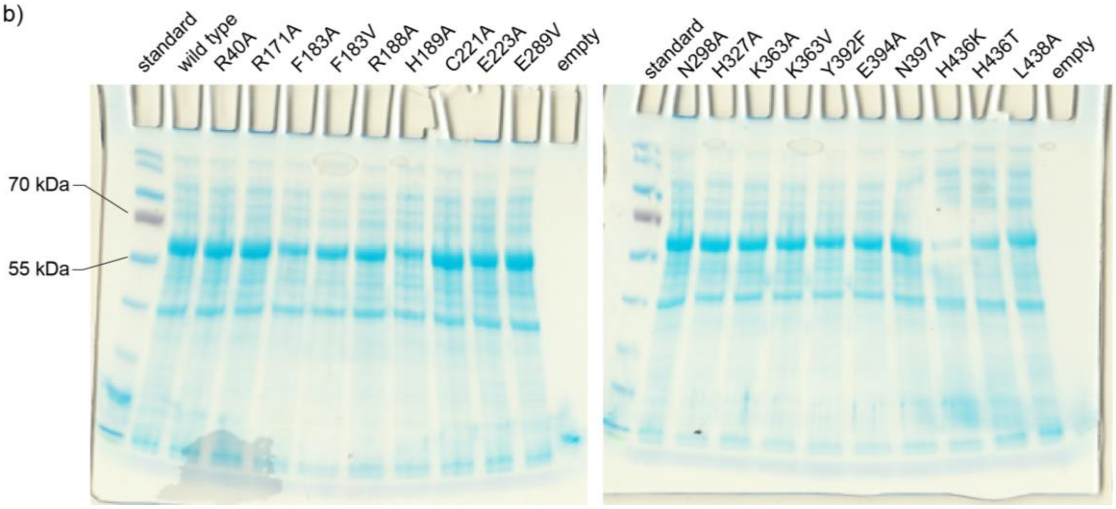

Figure S3. SDS-PAGE of a) Ni-affinity purification of His_6-tagged *Ec*AroY (wt), b) heterologous expression of *Ec*AroY variants; a band corresponding to the desired protein mass could be observed for all mutants.

Sequencing result of ubiX amplification from E. coli

5'–ATGAAACGACTCATTGTAGGCATCAGCGGTGCCAGCGGCGCGATTTATGGCGTGCGCTTATTACAGGTTCTGCGCGATGTCA
CAGATATCGAAACGCATCTGGTGATGAGCCAGGCAGCGCGCCAGACCTTATCCCTCGAAACGGATTTTTCTCTGCGCGAAGTGCAGGCATTAGCC
GATGTCACGCACGATGCGCGCGATATTGCCGCCAGCATCTCTTCCGGTTCTTTCCAGACGCTGGGGATGGTGATTTTACCCTGTTCAATCAAAACC
CTTTCCGGCATTGTCCATAGCTATACTGATGGCTTACTGACCCGTGCGGCAGATGTGGTGCTGAAAGAGCGTCGCCCGTTGGTGCTCTGCGTGCG
TGAAACACCATTGCACTTAGGCCATCTGCGTTTAATGACTCAGGCGGCAGAAATCGGTGCGGTGATTATGCCTCCCGTTCCGGCGTTTTATCATCG
CCCGCAATCCCTTGATGATGTGATAAATCAGACGGTTAATCGTGTTCTTGACCAGTTTGCGATAACCCTTCCTGAAGATCTCTTTGCCCGCTGGCAG
GGCGCA–3'

Figure S4. *E. coli* strain DA24692 UbiX gene.

Preliminary Results

Enzyme screening

Table S5. Carboxylation activity test of *p*-carboxylase candidates.

	Carboxylation (%)	
Biocatalyst	Lyophilized cell-free extract (30 mg/mL)	Lyophilized whole-cells (30 mg/mL)
*Ec*AroY	13	10
4-HBD_*Ec*[b]	< 1	< 1
4-HBD_*Cp*[b]	< 1	< 1
3,4-DHBD_*Ci*[c]	–[d]	< 1
decarboxylyase_*Dg*	2	3

Standard carboxylation conditions using 30 mg/mL of the biocatalyst with 10 mM substrate concentration (**4**) and 3 M $KHCO_3$ as CO_2-source were applied. Conversions were deduced from HPLC-peak areas after calibration. [a] Catechol was used as substrate for *Ec*AroY and *Ci* as well as for decarboxylyase_*Dg*; phenol was used for 4-HBDs from *Ec* and *Cp*. [b] Even after cultivation and testing under semianaerobe conditions (nitrogen atmosphere in cultivation flask) no activity could be detected. [c] Both preparations resulting from expression with chaperones 1 and 3 proved inactive. [d] Not determined.

Table S6. Dearboxylation activity test of *p*-carboxylase candidates.

	Decarboxylation (%)	
Biocatalyst	Lyophilized cell-free extract (30 mg/mL)	Lyophilized whole-cells (30 mg/mL)
*Ec*AroY	> 99	97
4-HBD_*Ec*[b]	< 1	< 1
4-HBD_*Cp*[b]	< 1	< 1
3,4-DHBD_*Ci*[c]	–[d]	11[e]
decarboxylyase_*Dg*	–	> 99[e]

Standard decarboxylation conditions using the denoted amounts of biocatalyst with 10 mM substrate concentration (**1**) were applied. Conversions were deduced from HPLC-peak areas after calibration. [a] **1** was used as substrate for *Ec*AroY and *Ci* as well as for decarboxylyase_*Dg*; 4-hydroxybenzoic acid was used for 4-HBDs from *Ec* and *Cp*. [b] Even after cultivation and testing under semianaerobe conditions no activity could be detected. [c] Both preparations resulting from expression with chaperones 1 and 3 proved inactive. [d] Not determined. [e] 30 mg of biocatalyst was used.

Characterization of AroY in conjugation with prFMN

Spectral properties of reconstituted AroY

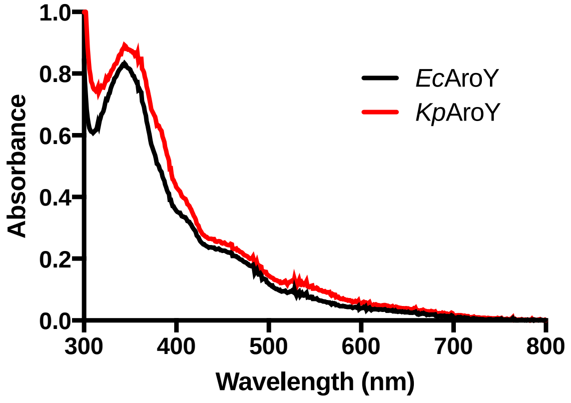

Figure S5. UV-Vis spectra of oxidized, reconstituted enzyme normalised to OD 280nm. These spectra were taken from protein used for activity assays, with only small hints of prFMN^{semiquinone} species.

a)

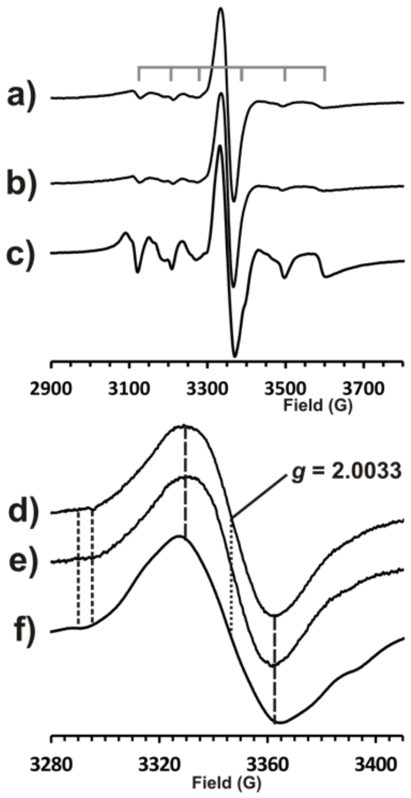

b)

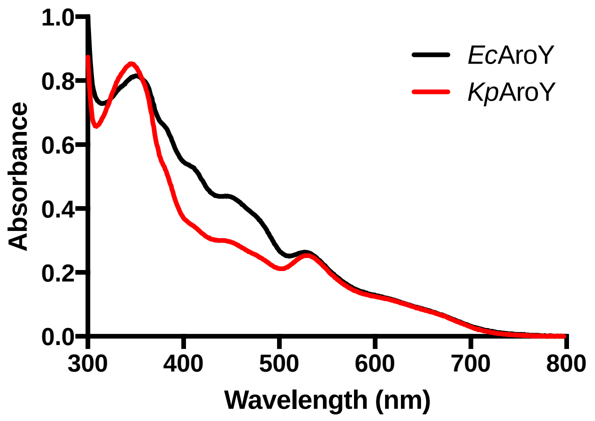

Figure S6. a) X-band EPR spectra of the reconstituted and oxygen activated *Ec*AroY a), d) and *Kp*AroY b), e) together with spectra of stalled *E.coli* UbiD enzyme[1] c), f) for comparison. AroY shows the characteristic 6 line spectrum of Mn^{2+} together with the Mn^{3+}-coupled prFMN radical signal. The slightly narrower line width and reduced splitting of the weak features at the high and low field extremes of d), e) and f) when compared to the UbiD radical arise from small differences in the orientation of prFMN relative to Mn^{3+}. Experimental parameters: microwave power 0.5 mW for a), b) and c), 10 µW for d), e) and f); modulation amplitude 7 G for a), b) and c), 1 G for d), e) and f); all spectra recorded at 20 K. b) UV-Vis spectrum of samples used for EPR which show hints of semiquinone at 530 nm, spectra were normalised to OD 280 nm.

Enzyme Kinetics

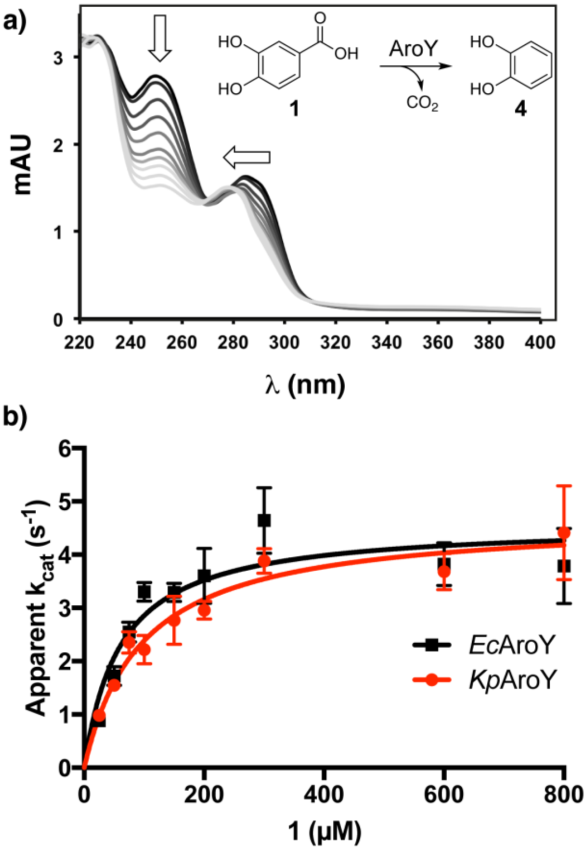

Figure S7. a) Spectral changes upon AroY catalyzed decarboxylation of **1**. b) Michaelis-Menten kinetics for both homologues with **1** as substrate. *Kp*AroY v_{max}^{app} = 4.7 ± 0.4 s^{-1}, K_m^{app} = 96 ± 23 µM; *Ec*AroY v_{max}^{app} = 4.6 ± 0.4 s^{-1}, K_m^{app} = 61 ± 17 µM.

Binding of FMN

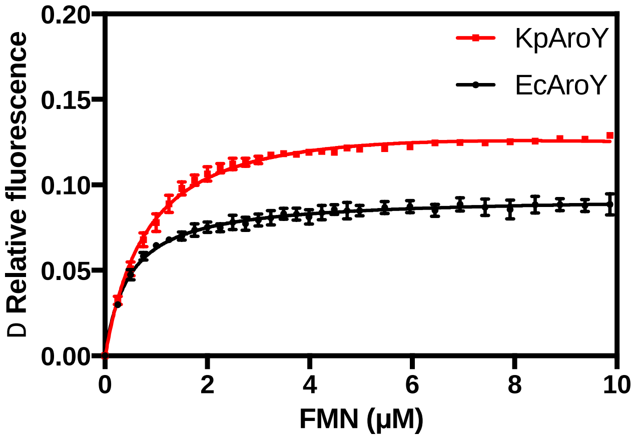

Figure S8. FMN binding in the presence of Mn^{2+} measured by tryptophan fluorescence quenching. Dissociation constants156± 68 nM and 93 ± 37 nM for *Kp*AroY and *Ec*AroY respectively.

pH study with EcAroY

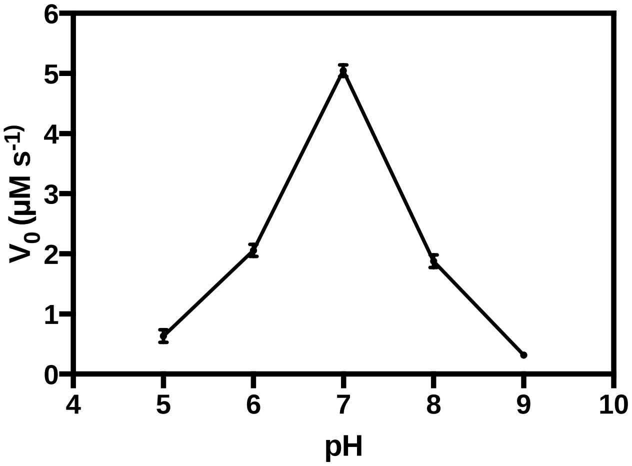

Figure S9. pH profile of decarboxylation of **1** using *Ec*AroY.

(De)carboxylation with AroY *E. coli* whole-cell preparations

Substrates not accepted by EcAroY

Table S7. Compounds **9** through **26** were not converted by *Ec*AroY under standard carboxylation conditions.

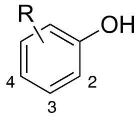

9–25

Compound	R	Carboxylation (%)	Compound	R	Carboxylation (%)
**9**	H	< 1	**18**	2-Cl	< 1
**10**	3-OH	< 1	**19**	2-Br	< 1
**11**	2-NH_2	< 1	**20**	2-NO_2	< 1
**12**	2-OMe	< 1	**21**	3-OH-5-nC_5H_{11}	< 1
**13**	2-Me	< 1	**22**	2-geranyl	< 1
**14**	3-Me	< 1	**23**	2-farnesyl	< 1
**15**	2-Et	< 1	**24**	2-OH-4-Me	< 1
**16**	2-*n*Pr	< 1	**25**	2-OH-3-CHO	< 1
**17**	4-vinyl	< 1	**26**	2-OH-4-CHO	< 1

Standard carboxylation conditions using 30 mg/mL of the biocatalyst with 10 mM substrate concentration (**4**) and 3 M $KHCO_3$ as CO_2-source were applied. Biotransformations were analyzed with HPLC.

Activity of EcAroY variants

To probe the role of amino acid residues in catalysis and binding of the substrate or the prFMN cofactor, a set of variants was generated based on structural information and a sequence alignment with other carboxylases from the UbiD superfamily (Figures S10 and S22).

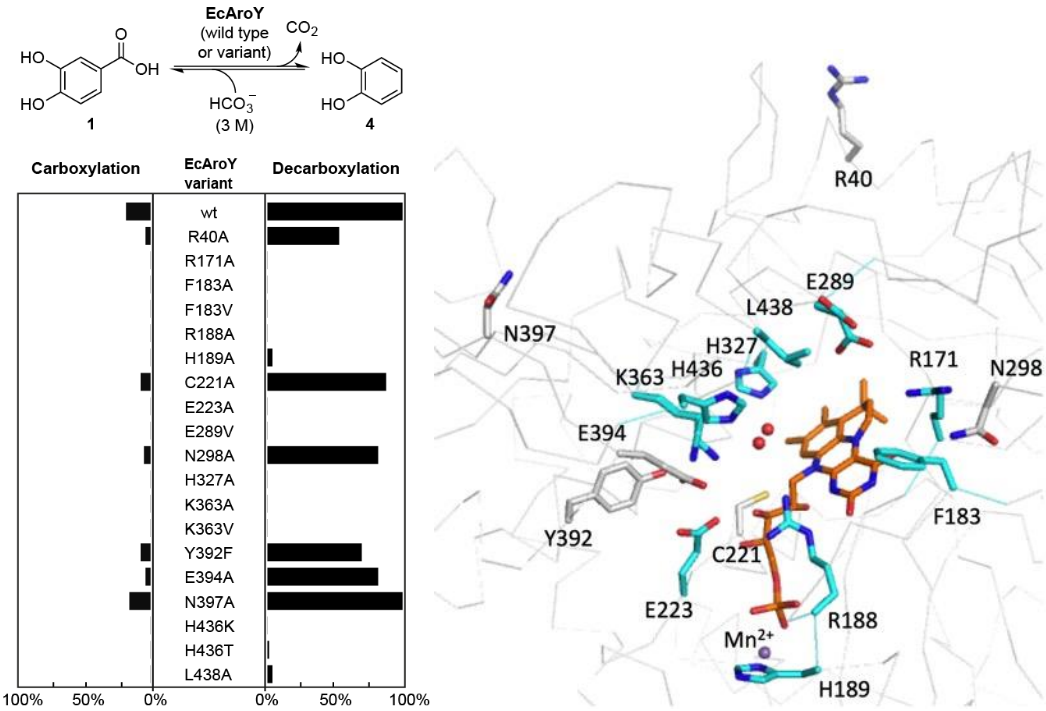

Figure S10. Carboxylation- and decarboxylation performance of *Ec*AroY variants. Exchanged residues and prFMN cofactor (orange) are shown in sticks representation. Residues whose mutation resulted in complete loss of activity are highlighted in cyan, others are colored in grey. Residues E289 and K363 are shown in two different conformations.

NMR for determining carboxylation regioselectivity

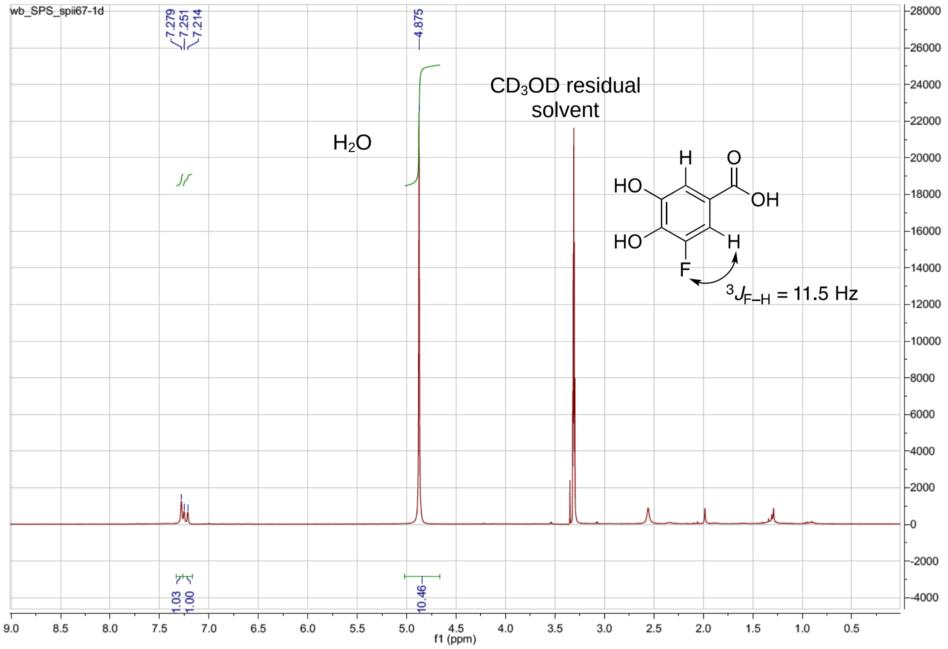

Figure S11. ^{1}H NMR of 2-fluoro-3,4-dihydroxybenzoic acid (carboxylation product of **6**) (300 MHz, CD_3OD).

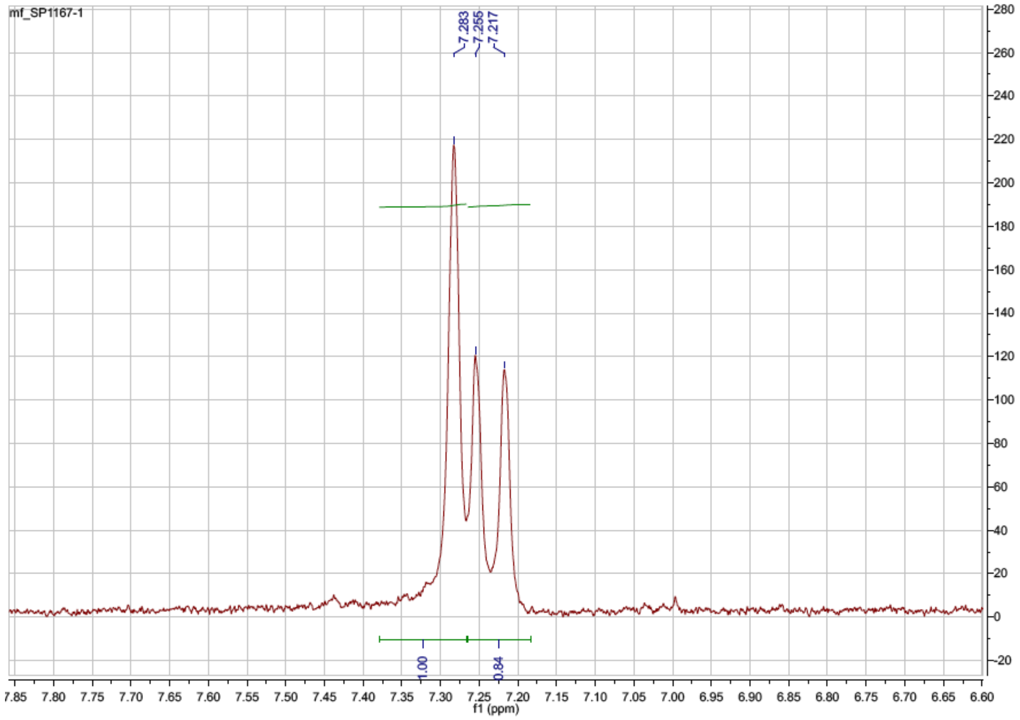

Figure S12. ^{1}H NMR of 2-fluoro-3,4-dihydroxybenzoic acid (zoom 6.6–7.8 ppm). F–H coupling $^3J_{F\text{–}H}$=11.5 Hz.

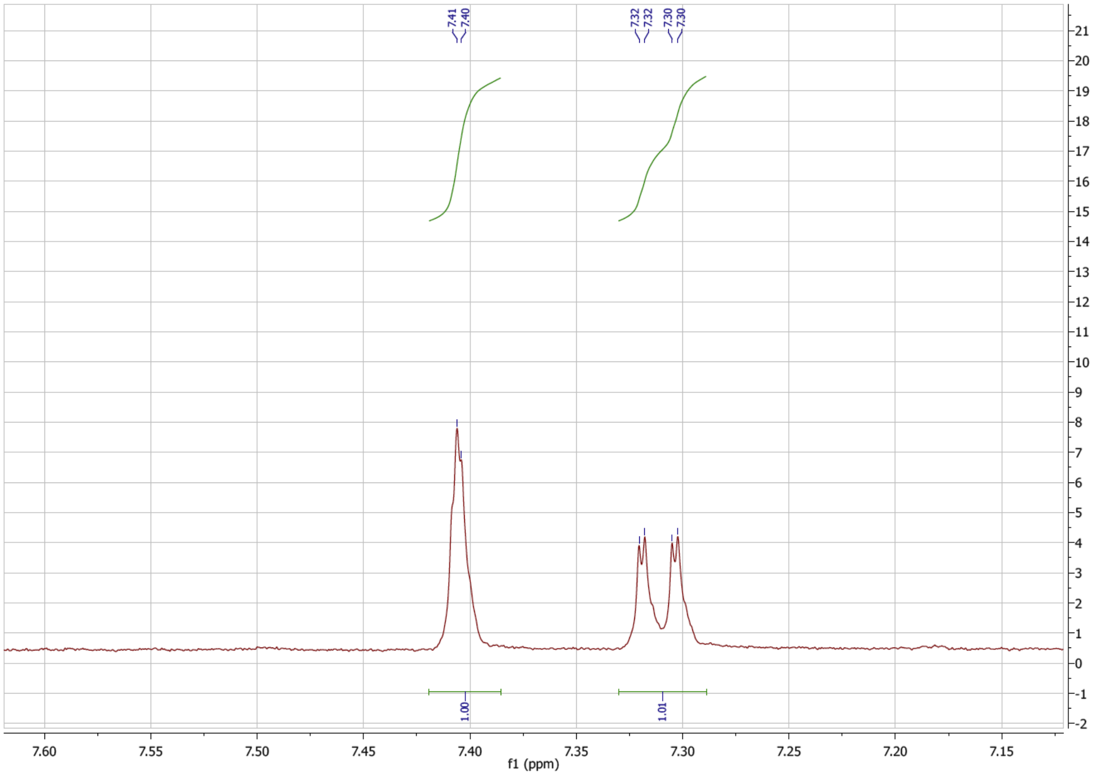

Figure S13. ^{1}H NMR of 2-fluoro-3,4-dihydroxybenzoic acid (zoom 7.0–7.6 ppm) (700 MHz, $(CD_3)_2CO$).

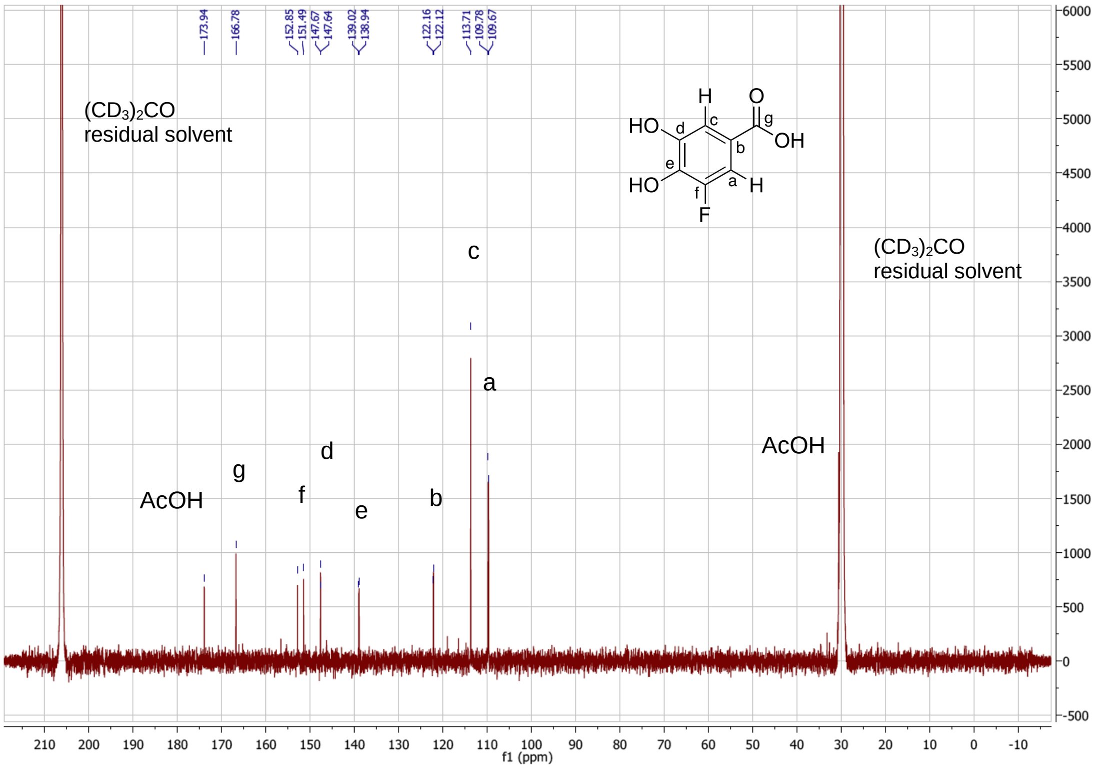

Figure S14. ^{13}C NMR of 2-fluoro-3,4-dihydroxybenzoic acid (175 MHz, $(CD_3)_2CO$).

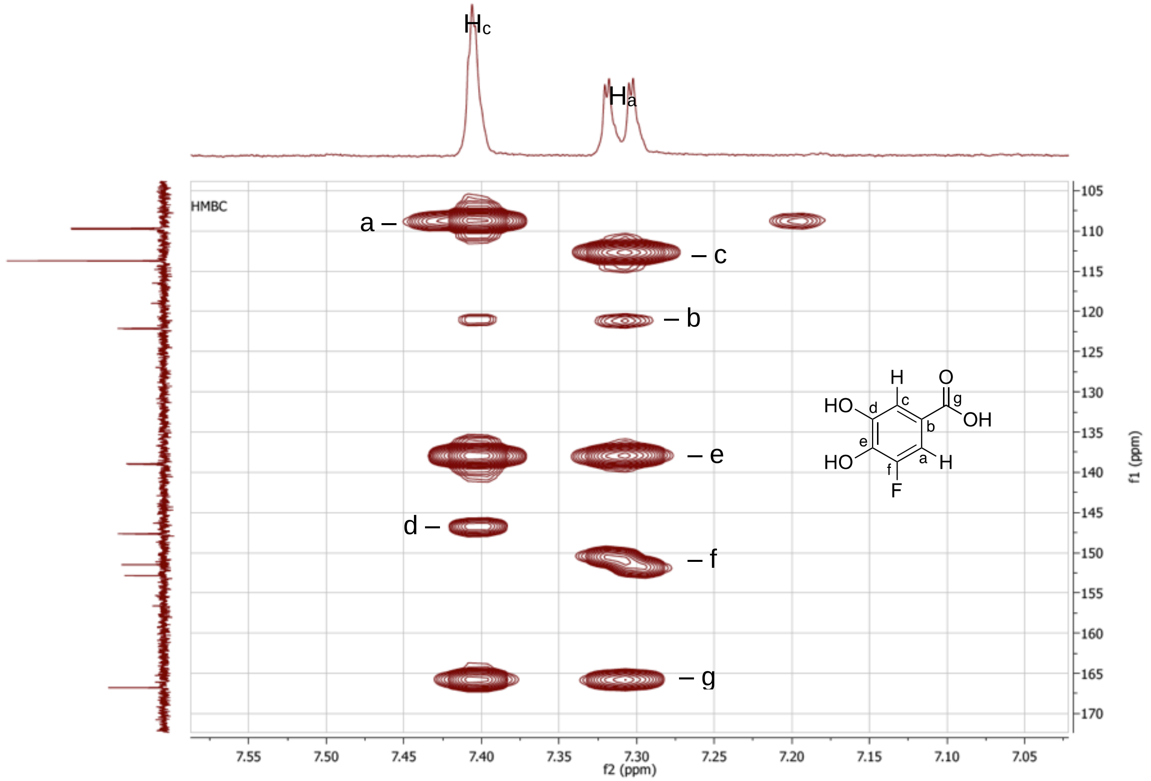

Figure S15. HMBC of 2-fluoro-3,4-dihydroxybenzoic acid (700 MHz, $(CD_3)_2CO$).

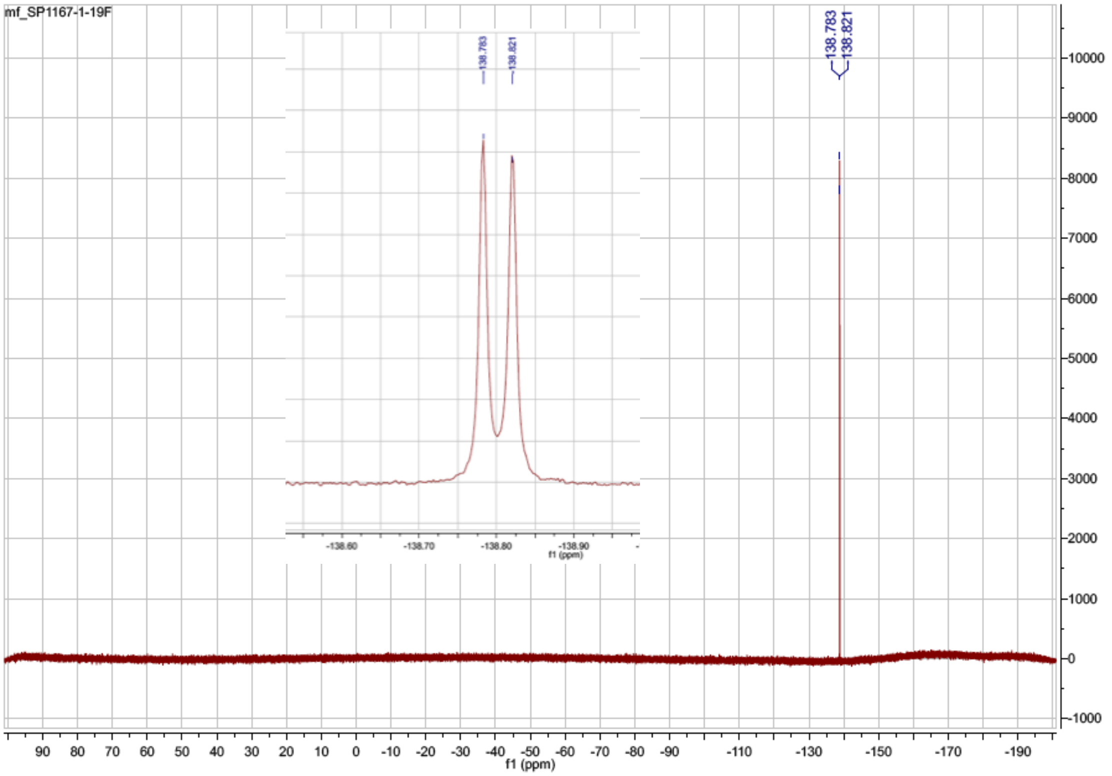

Figure S16. Full scale ^{19}F NMR (300 MHz, CD_3OD) spectrum of 2-fluoro-3,4-dihydroxybenzoic acid with insert showing a zoom between –138.4 and –139.4 ppm. F–H coupling $^3J_{F-H}$=10.97 Hz.

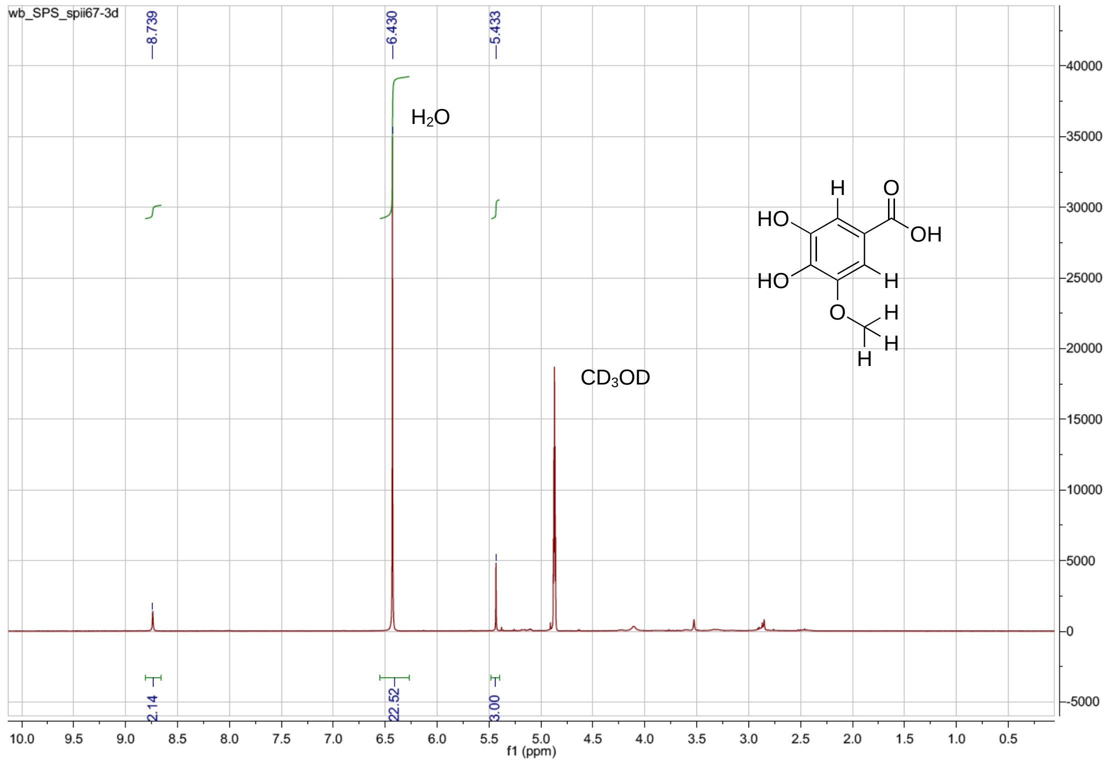

Figure S17. Full scale ^1H NMR (300 MHz, CD_3OD) spectrum of 2-methoxy-3,4-dihydroxybenzoic acid (carboxylation product of **7**). In agreement with literature: ^1H NMR (500 MHz, CD_3OD) □ (ppm): 7.17 (s, 2H), 3.86 (s, 3H).[2]

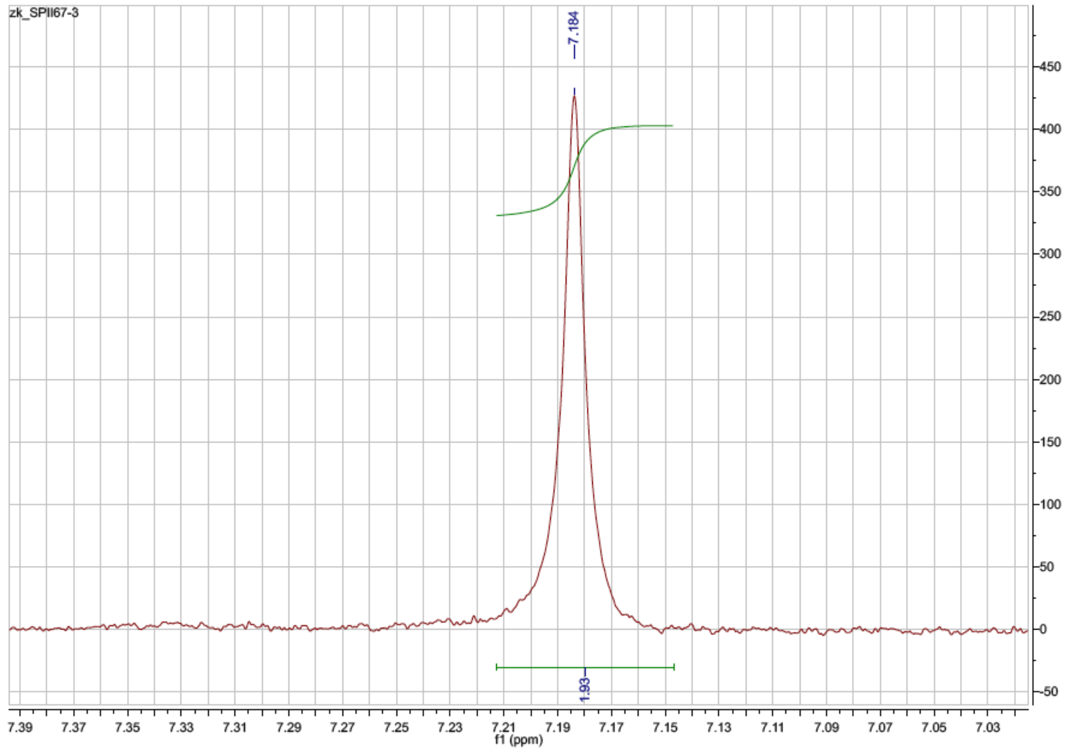

Figure S18. Zoom (7.0–7.4 ppm) of ^1H NMR spectrum of 2-methoxy-3,4-dihydroxybenzoic acid.

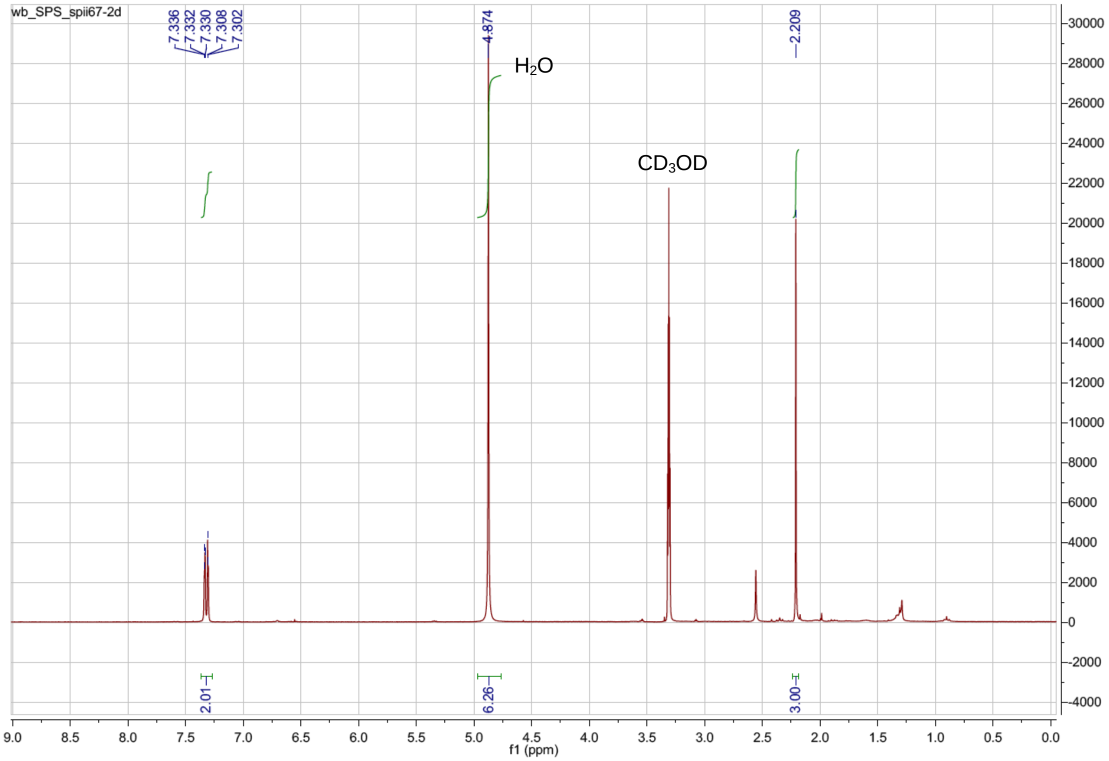

Figure S19. Full scale ^{1}H NMR (300 MHz, CD_3OD) spectrum of 2-methyl-3,4-dihydroxybenzoic acid (carboxylation product of **8**).

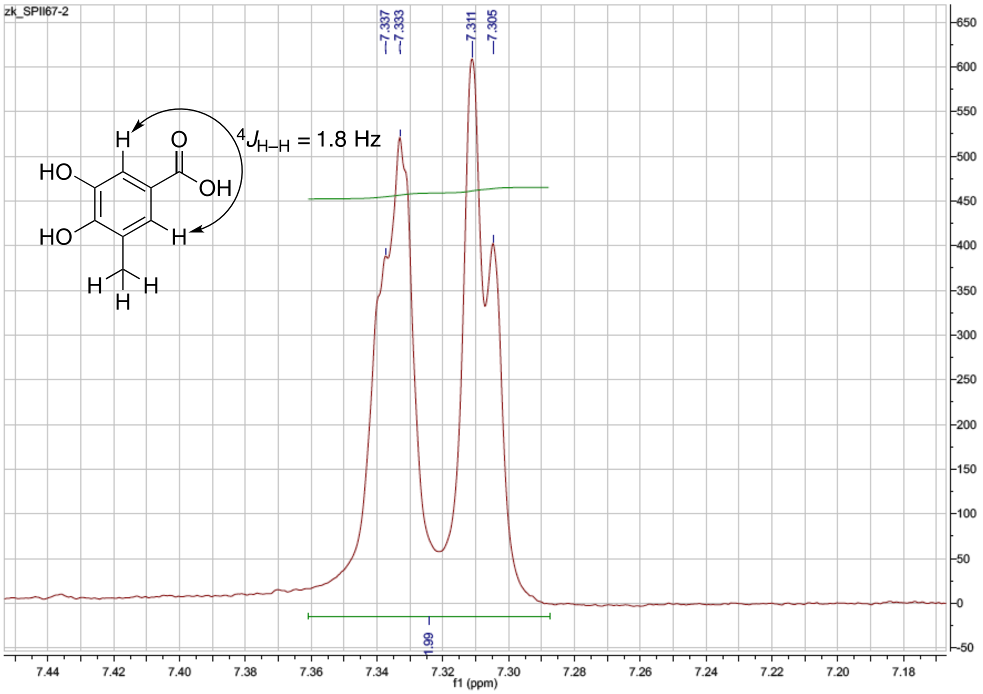

Figure S20. ^{1}H NMR of 2-methyl-3,4-dihydroxybenzoic acid (zoom 7.16–7.47 ppm): characteristic meta coupling of $^{4}J_{H\text{–}H}$ = 1.8 Hz.

Deuterium exchange experiments

Experiments show the decrease in peaks associated with protons *para* to central hydroxyl groups, as they are replaced by deuterium. Shifts in peaks also correspond to coupling effects as shown above.

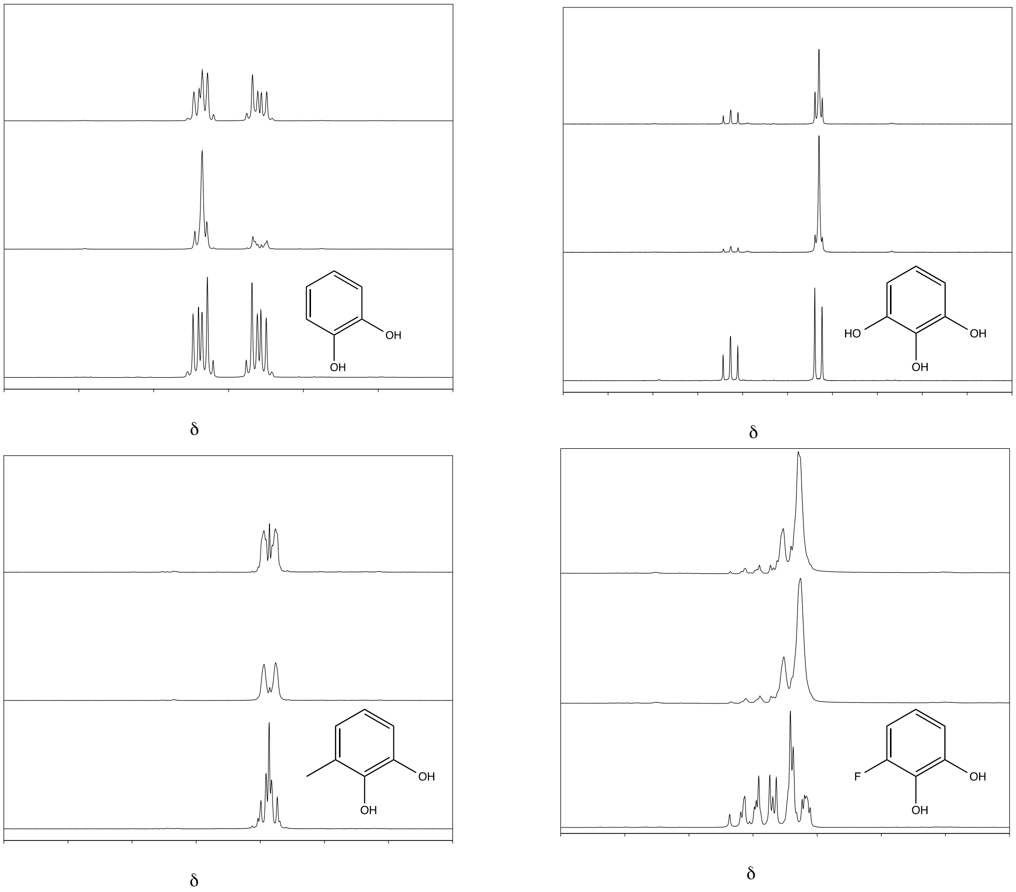

Figure S21. Deuterium exchange experiments with *Ec*AroY (E) and *Kp*AroY (K).

Structural Biology

Sequence alignment with other (de)carboxylases

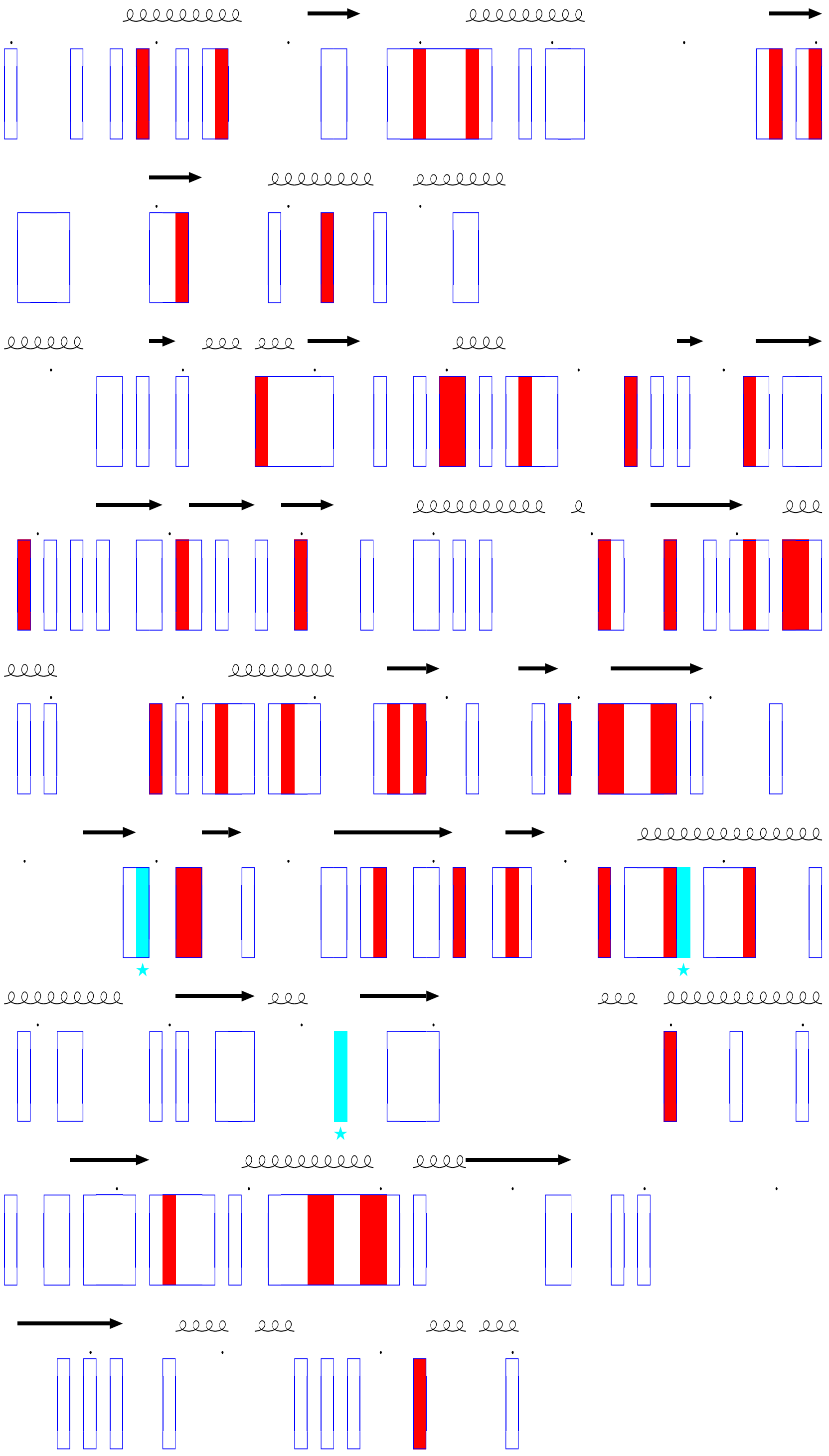

Figure S22. Sequence comparison of *Ec*AroY, *Kp*AroY, Fdc1, UbiD and PA0254. (De)carboxylases from *Pseudomonas aeruginosa PAO1* (PDB: 4IWS),[29] *E. coli* (PDB: 2IDB, 5M1C, 5M1B, 5M1D, 5M1E)[1] and *Aspergillus niger* (PDBs: 4ZAA, 4ZA5, 4ZA7, 4ZA8, 4ZA9)[30] exhibit the same fold and belong to the UbiD-family. Identical residues are shown in red boxes. Secondary structure elements of AroY are shown above the sequence alignment. Important amino acid residues in the active sites of AroY enzymes are marked with an asterisk and shown in cyan boxes. This figure was prepared using T-coffee[12] and ESPript 3.[13]

Crystallization and structure analysis

The apo-form of *Kp*AroY crystallized readily, however crystals of the reconstituted *holo* enzyme could not be obtained. In contrast, *holo-Ec*AroY crystals diffracted to 2.1 Å and clear density for cofactor was observed (Figure S24). When comparing *Ec*AroY *apo* and *holo* structures only a minor alteration of the prFMN-binding domain is observed (RMSD of 0.74 Å). Both enzymes crystallize as hexameric assemblies, arranged as a trimer of dimers (Figure 2 and S23). The same quaternary structure is observed in solution by cryo-EM for *Ec*AroY (Figure 2a), as well as for the X-ray structure of the related UbiD enzyme.[1]

Despite various attempts at soaking or co-crystallizing *Ec*AroY with compounds **1**–**6** under different conditions, no interpretable electron density corresponding to a bound substrate could be observed.

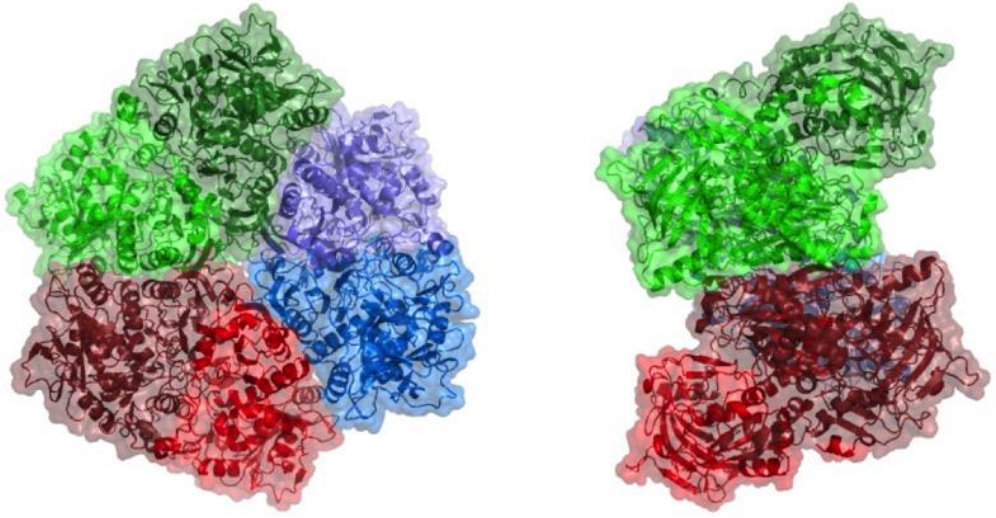

Figure S23. Hexameric conformations of *Kp*AroY as observed in the crystal, colours denote dimer pairs. The right image shows a 90° rotation about the Y axis relative to the left image.

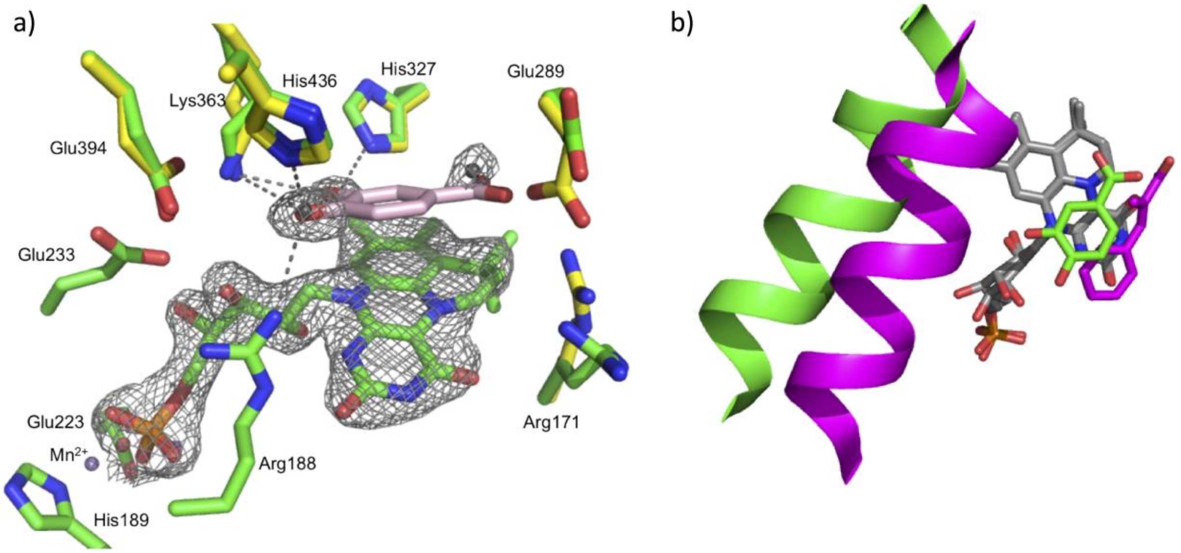

Figure S24. AroY structure and active site analysis a) Active site overlay of *holo-Ec*AroY (green) with *apo-Kp*AroY (yellow). Residue numbering is identical for both homologues. Water molecules (grey) in the vicinity of the cofactor that were used to model the binding of protocatechuic acid (pink) with the two hydroxyl groups replacing the water molecules interacting with the His-Lys-Arg cluster. Hydrogen-bonding interactions are shown by dashed lines (grey). Difference density around the water molecules and the prFMN cofactor is contoured at 3σ. b) An overlay of the Fdc1-methyl cinnamic acid complex (4ZA7, in magenta) with the *Ec*AroY protocatechuic acid model (in green) showing the *C*-terminal α-helix of the prFMN-binding domain. The prFMN coordinates were used for superimposition (in grey).

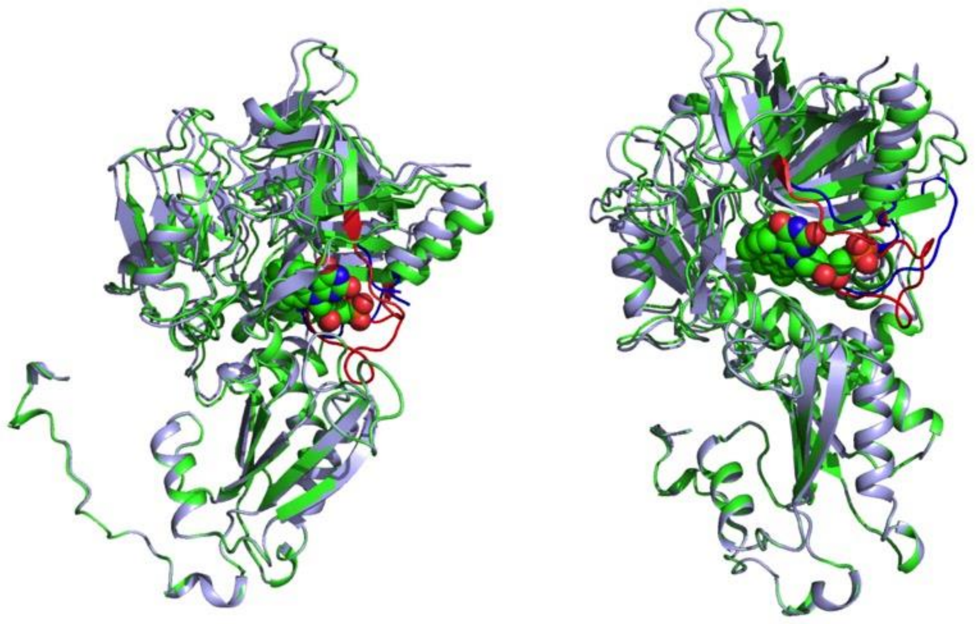

Figure S25. *Apo-Ec*AroY (light blue) overlaid on *holo-Ec*AroY (green). Only small domain motions are in evidence, with RMSD = 0.74Å. Metal and cofactor binding loops are shown in blue (*apo*) and red (*holo*), and can be seen to be closer to prFMN in *holo* structures. prFMN cofactor is shown in spheres. Overlays reveal two mobile loops, comprising residues 182–189 and 220–233, respectively, that alter conformation in response to the presence of prFMN and Mn^{2+}. In the *apo* structures, these loops are slightly disordered, while the *holo* structure reveals a network of polar contacts established with the manganese ion and the phosphate group of prFMN.

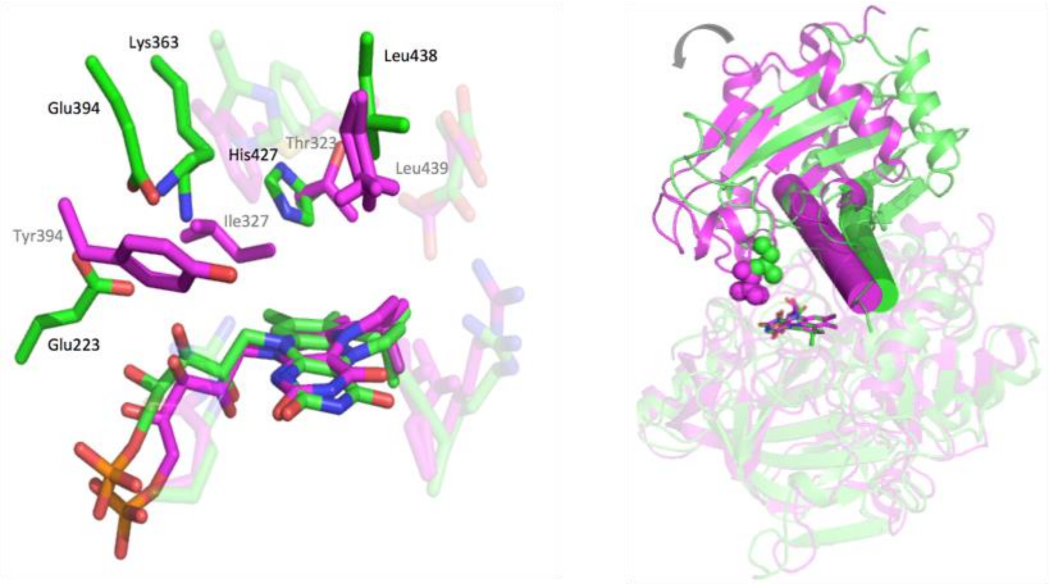

Figure S26. Fdc1 (4ZA4) overlaid with *Ec*AroY (both *holo*). *Ec*AroY (green) shows more open state than Fdc1 (magenta). Left: Active site residues of Fdc1 labelled in grey, *Ec*AroY in black. Residues that align well are translucent and unlabelled. Position of Leu438 in AroY is higher than Leu439 of Fdc1, also evidenced in right panel as spheres. Right: Grey arrow shows proposed domain motion. For clarity, *C*-terminal helix domains are removed.

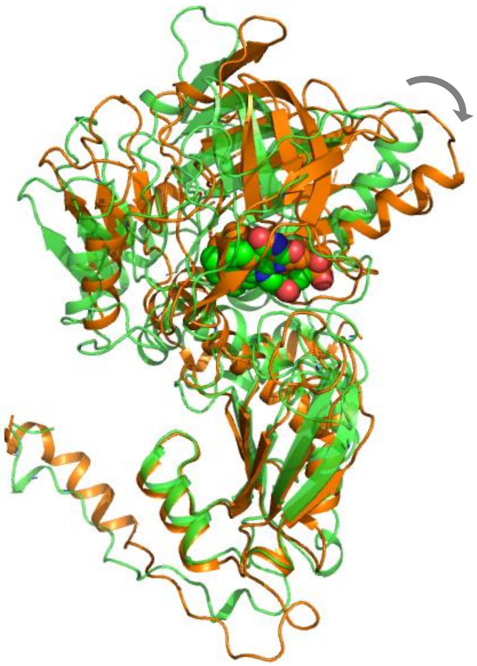

Figure S27. UbiD (5M1E in orange) overlaid with *Ec*AroY (green) (both *holo*). UbiD appears to be more closed than AroY, with proposed motion shown by grey arrow. prFMN shown in spheres coloured according to model.

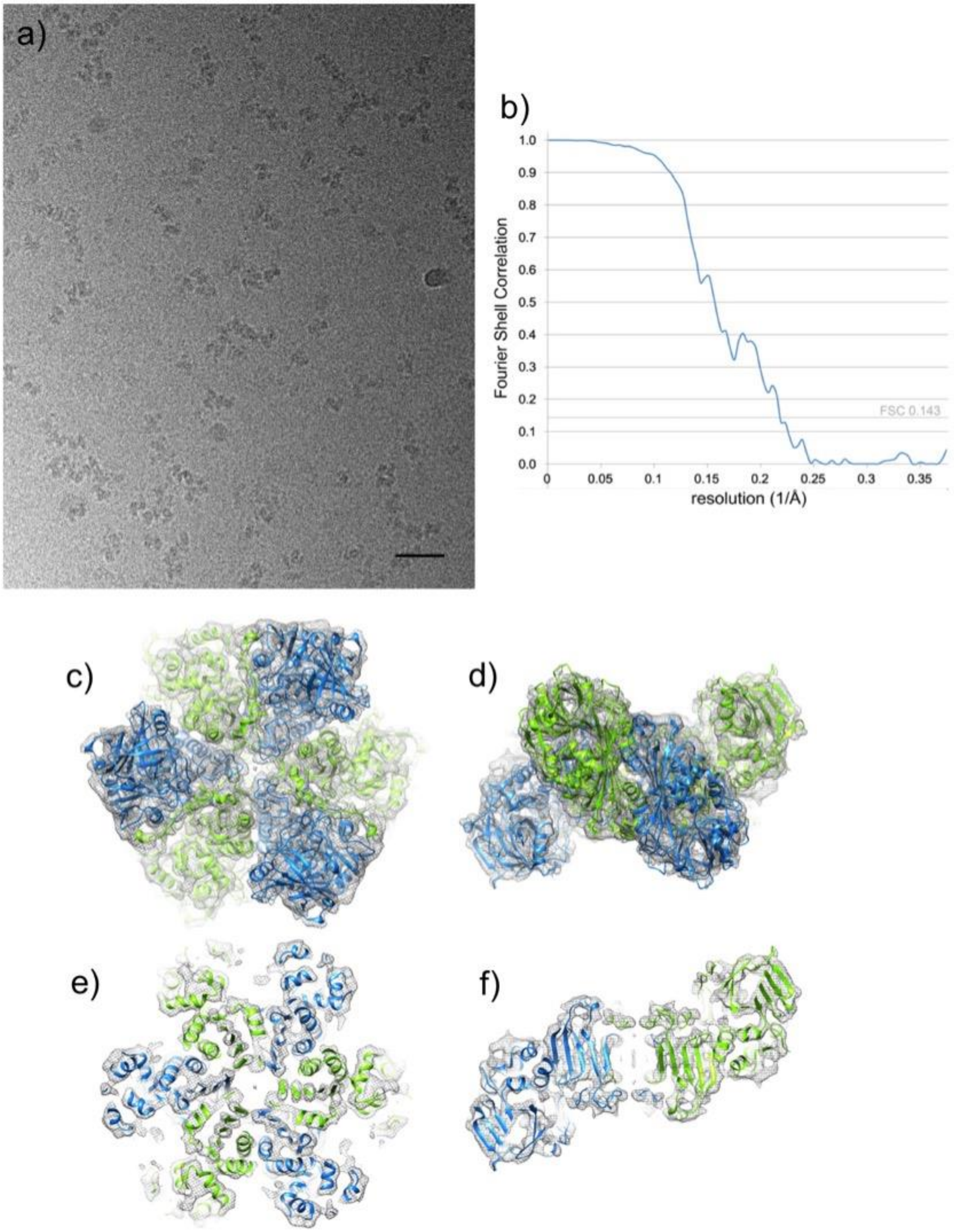

Figure S28. *Ec*AroY Cryo-EM data. a) Representative motion-corrected cryo-electron micrograph; scale bar: 10 nm. b) Fourier Shell Correlation plot of the final map, indicating a map resolution of 4.6 Å. The EM map with the structure docked in c) top view and d) side view. e) And f) show slices of the map with the docked model in the same orientations as c) and d), respectively.

Quantum Chemical Calculations

To shed more light on the reaction mechanism of the prFMN-dependent decarboxylation of protocatechuic acid (**1**), we resorted to density functional theory (DFT) calculations, employing a large model of the active site consisting of 283 atoms. In the optimized structure of the **E:S** complex, substrate **1** is bound such that the carboxylate moiety forms hydrogen bonds with Arg171 and Glu289, while the hydroxyl groups are hydrogen-bonded to Arg188, His327, Lys363 and His436 (Figure 2b in the main text). This is consistent with the binding mode discussed on the basis of the crystal structure and mutational studies. Interestingly, the proton of the *para*-hydroxyl group moves spontaneously to the Lys363 residue. Apparently, the interactions with the two glutamate residues (Glu223 and Glu394) make the side chain of Lys363 more basic.

Starting from the **E:S** complex, different mechanistic proposals were examined. First, we considered the viability of a 1,3-dipolar cycloaddition intermediate, following the mechanism proposed for the prFMN-dependent Fdc1.[30–33] This mechanism starts with the formation of two C–C bonds between the substrate and the cofactor. However, despite numerous attempts, no such cycloaddition intermediate could be identified in the calculations. The C–C bond between the C4 of prFMN and the Cβ of the substrate always breaks during optimization and the calculations converge to a quinoid-intermediate (**Int1** in Figure 2c in the main text). Forcing the formation of the cycloadduct, by constraining the C4–Cβ bond distance to 1.5 Å, resulted in a very high energy, ca. 37 kcal/mol higher than **E:S** (Figure S29). The high energy appears due to the loss of the aromaticity of the substrate ring, and to the high level of strain induced in this adduct. Thus, these results suggest in the case of AroY a 1,3-dipolar cycloaddition mechanism is less likely.

Instead, we found that the mechanism shown in Figure 2c in the main text, which has chemical similarity to that proposed for the phenolic acid decarboxylases (PAD),[28a] has significantly lower energy barriers as compared to the 1,3-cycloaddition mechanism. The quinoid intermediate **Int1** is calculated to be only 7.6 kcal/mol higher than the **E:S**, and the barrier for its formation (**TS1**) is only 11.6 kcal/mol (Figure S30). From **Int1**, C–C bond cleavage takes place to generate CO_2 (**Int1**→**Int2**), with an accumulated barrier of 19.9 kcal/mol, constituting the rate-limiting barrier of the reaction. Next, a proton transfer from Glu289 to the decarboxylated intermediate takes place (**Int2**→**Int3**), followed by C–C bond cleavage to generate the catechol product (**Int3**→**Int4**). In the related Fdc1, the CO_2 molecule is then released from the active site, on the basis of kinetic isotope effects experiments and QM/MM calculations.[31] The optimized structures of all intermediates and transition states along the reaction pathway are given in Figure S31 and S32, and the Cartesian coordinates are given below.

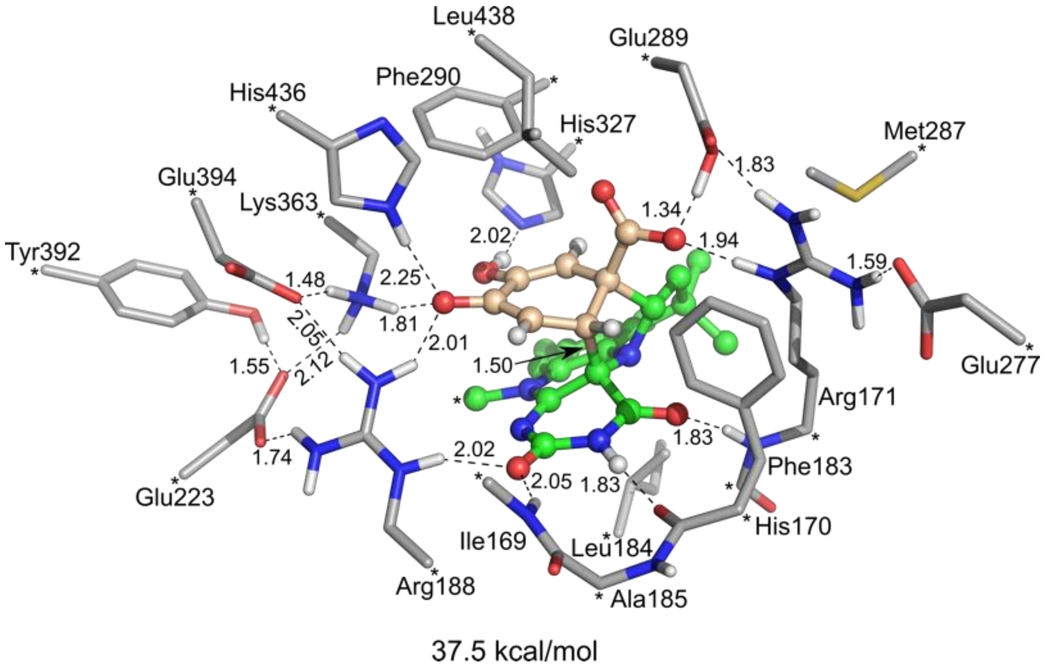

Figure S29. Optimized structure of the cycloadduct intermediate by constraining the indicated C–C bond distance to 1.50 Å as shown in the figure. The calculated energy relative to the **E:S** complex is also given. For clarity, only polar hydrogen atoms and the hydrogen atoms on the substrate are shown.

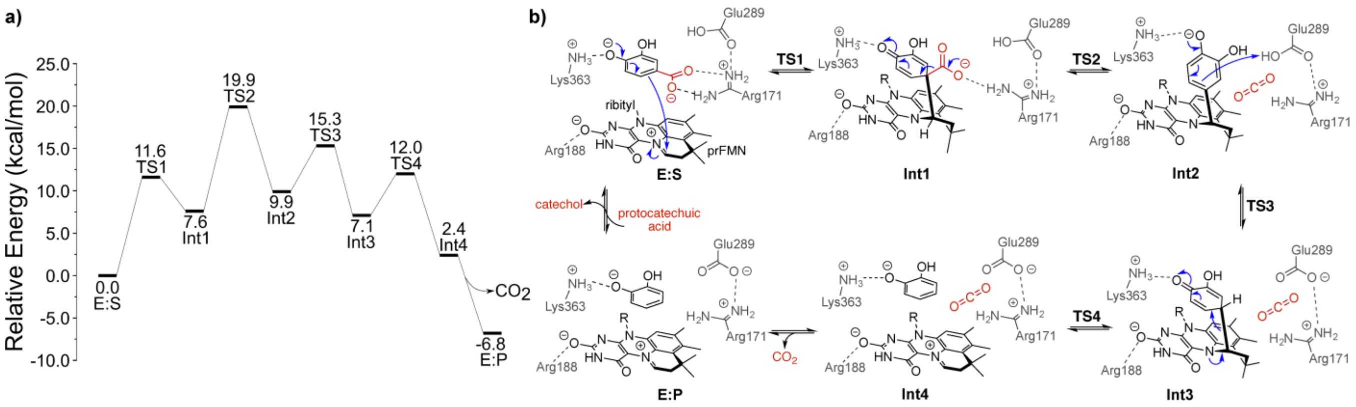

Figure S30. a) Calculated energy profile for the suggested mechanism. b) The mechanism for decarboxylation is initiated by a nucleophilic attack of the substrate at the C1' iminium (E:S–Int1). After loss of CO_2 (Int1–Int2), protonation of the monocovalent adduct by Glu 289 occurs (Int2–Int3). The covalent cofactor-substrate bond is broken again and liberates the product catechol and CO_2 (Int3–Int4).

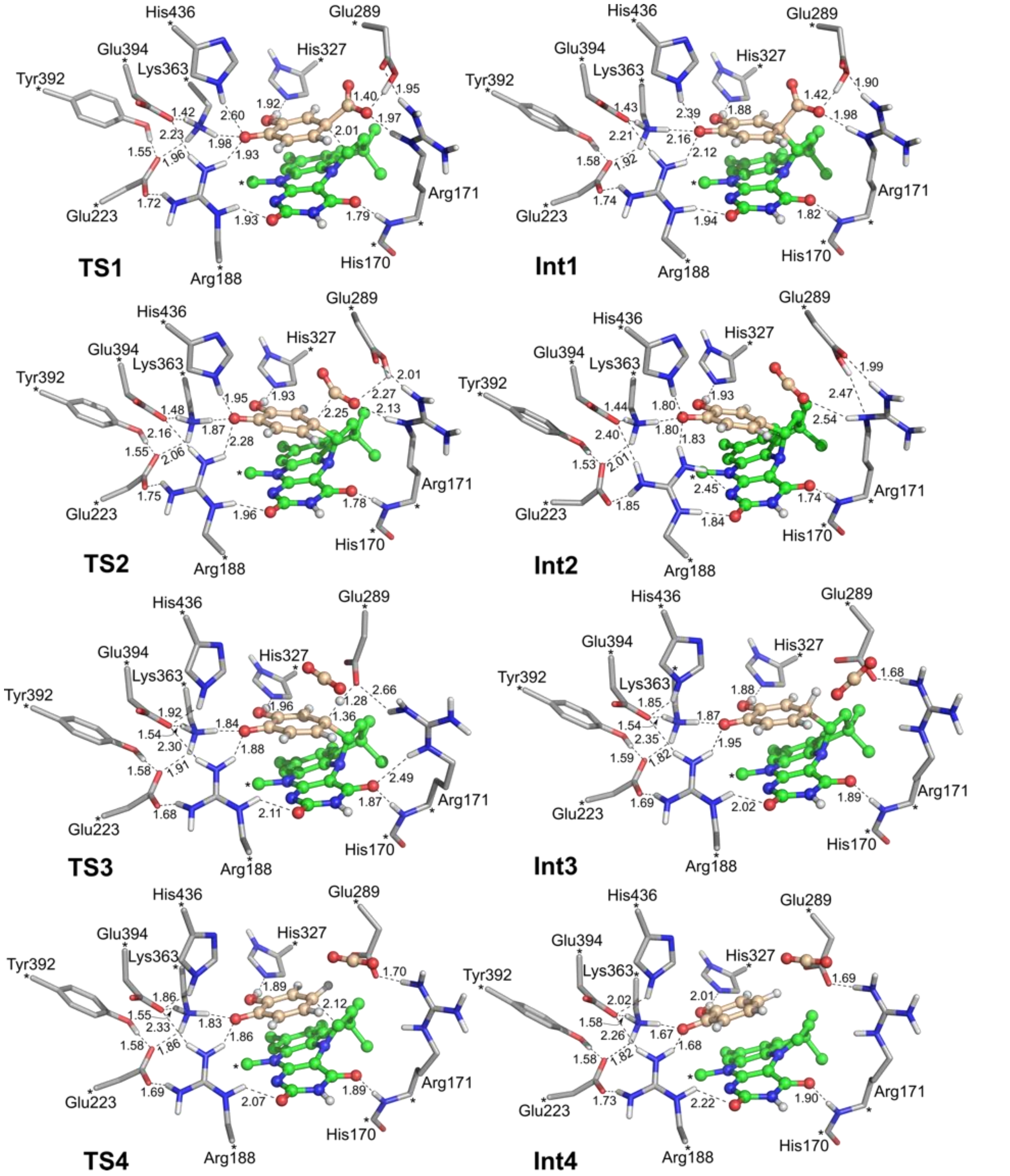

Figure S31. Optimized structures of the transition states and intermediates along the reaction before the release of CO_2 for the suggested mechanism. For clarity, only selected groups are shown here. Only polar hydrogen atoms and the hydrogen atoms on the substrate are shown.

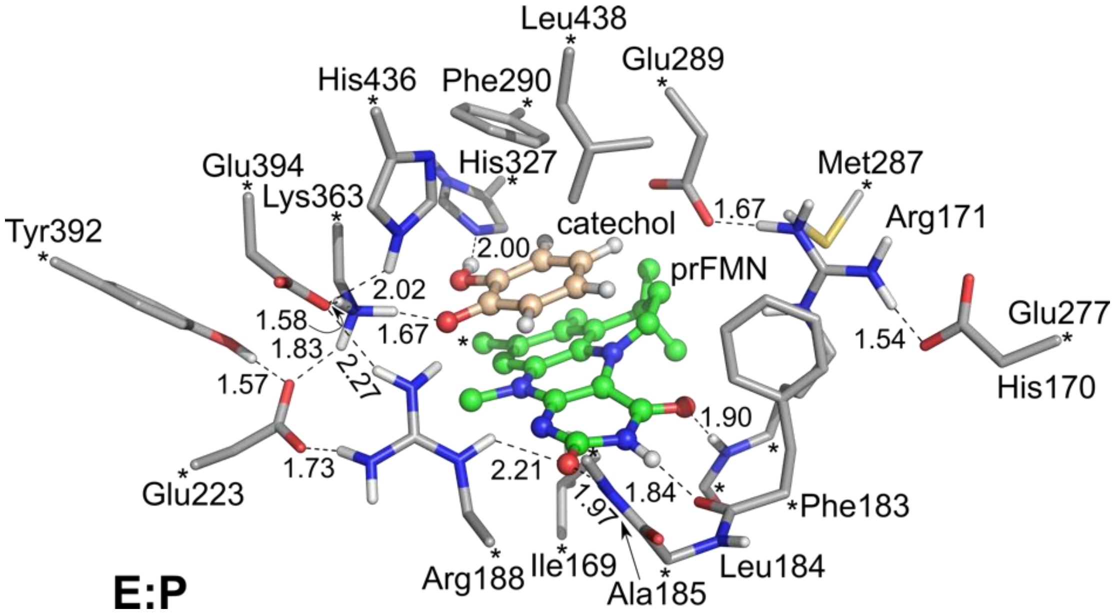

Figure S32. Optimized structure of the enzyme-product complex after the release of CO_2. For clarity, only polar hydrogen atoms and the hydrogen atoms on the substrate are shown.

Cartesian coordinates

E:S (0.0 kcal/mol)

C 0.72276500 0.42403400 -7.89138800
C 0.58635400 1.74301900 -7.12799300
C 0.73605200 2.93416600 -8.08920800
C 1.59957000 1.79537500 -5.98252900
C 0.51625500 4.30145600 -7.43960700
C 4.62028500 0.17497000 -5.30975500
O 5.08609700 0.71003000 -6.31311500
N 5.08254600 0.32014900 -4.05577400
C 6.31355300 1.05266900 -3.85881700
C 6.51253000 1.52894900 -2.41511300
C 6.52192700 0.43818200 -1.33243500
C 6.72994900 1.03934200 0.07833200
N 6.04186900 0.32250100 1.13649000
C 6.54951900 -0.67703000 1.87682500
N 7.78295500 -1.14926600 1.69890200
N 5.74310500 -1.19792200 2.82501300
C 6.41990800 -4.79700100 -1.30498200
C 5.06612300 -5.22531200 -1.84848000
O 4.29157900 -4.41468500 -2.36195400
C 6.28340900 -3.54295400 -0.41898700
C 5.58838400 -3.87621300 0.87834000
C 6.31344000 -4.43984300 1.93866400
C 4.21545500 -3.66581800 1.03601100
C 5.67377100 -4.79501200 3.12600200
C 3.57240700 -4.02232600 2.22177700
C 4.29685500 -4.59333500 3.26664100
N 4.78275200 -6.55474000 -1.78938200
C 3.46256700 -7.05604500 -2.14314700
C 2.74294700 -7.47375000 -0.86829100
O 3.16420300 -8.43112900 -0.21492200
N 1.68586000 -6.72203300 -0.50737600
C 1.03291900 -6.99186000 0.75628500
C 13.07775100 -2.39677300 2.93064100
C 12.39625100 -1.58694300 1.81752300
C 10.83961700 -1.49666800 1.85892200
O 10.21913700 -2.27951900 2.62451300
O 10.34604800 -0.63390600 1.06683200
C 8.23556800 6.40398000 1.21292900
C 6.02748700 4.71692700 1.51413000
S 6.76758100 5.84022300 0.27740200
C -1.86249100 6.60090800 2.57736300
C -2.56911600 5.36696800 3.09304700
C -3.45377500 5.44555600 4.17653900
C -2.37785800 4.12082800 2.48476200
C -4.12030700 4.31106600 4.64054700
C -3.06297900 2.98878200 2.92785400
C -3.93022900 3.07547000 4.01814300
C -13.95199700 -0.89835800 0.76877000
C -12.71074500 -0.55738000 -0.02950700
C -11.57070700 -1.37139700 -0.00228400
C -12.64433700 0.60907900 -0.80257500
C -10.41210900 -1.04591200 -0.70691900
C -11.49787700 0.95119700 -1.51430900
C -10.36131100 0.12756000 -1.47810300
O -9.27741600 0.50522100 -2.18859800
C -0.59610600 0.64809500 8.24748700
C -0.43410000 1.39615500 6.91993600
C -0.10798000 0.50311300 5.70976800
C -0.15606100 1.31672500 4.41184600
C 1.24929600 -0.20241600 5.83258900
N -2.59303900 -4.91494700 -1.40576700
N -3.81522200 -3.30262400 -0.17214100
N -4.93480100 -4.93631000 -1.29719000
N -3.27047700 6.15243700 -0.81342800
N -1.49303800 4.83676900 -0.70523000
N -5.67439400 -0.25473000 -0.17317300
N -3.66953000 -0.25553900 5.44129000
N -4.13353700 -1.25566000 3.51019100
N -0.88004300 0.03494500 -2.58370400
N 1.73330800 0.91505400 -2.00726000
N -0.29198300 -2.16318500 -2.11194600
N 1.98366800 -2.75210400 -1.93639900
C -9.51030400 -3.83053200 -3.37082100
C -8.71206500 -2.55284400 -3.62782600
C -7.54008300 -2.39420100 -2.64381900
C -8.71399200 -0.96273400 3.62879100
C -8.41656500 -1.49808700 2.21398900
C -7.22408600 -2.48382100 2.17744300
C -1.24452800 -6.77127500 -2.47127800
C -2.58250100 -6.02433100 -2.37147500
C -3.75247700 -4.36588400 -0.97405400
C 2.30930100 4.52476900 5.06848800
C 3.48600700 3.54845300 5.05152600
C 3.82121600 2.94947800 3.69355300
C -2.28429200 8.48621800 -1.03721500
C -2.17390400 6.99652400 -0.92849400
C -2.80847200 4.87179200 -0.67783900
C -1.09086200 6.15059000 -0.85887000
C -6.50921800 1.64643600 1.22609700
C -6.39189700 1.04478800 -0.16716700
C -5.42067900 -1.28791400 6.94568500
C -4.73994100 -1.12083200 5.61722500
C -3.33482600 -0.36957900 4.16383400
C -5.03805200 -1.75006100 4.42839200
C -2.28080700 -0.37042600 -2.73229800
C -0.52351900 1.36054700 -2.80247000
C -1.45794000 2.26555100 -3.32119200
C -1.11064100 3.56949600 -3.63132200
C -2.17608000 4.48682800 -4.17901000
C 0.20195700 4.03792700 -3.40272400
C 0.53621000 5.42489900 -3.90570700
C 1.12460200 3.20306700 -2.74772900
C 0.76214300 1.85413200 -2.50722100
C 0.09098500 -0.89632900 -2.26013200
C 1.42834700 -0.46808900 -2.14906300
C 2.45594300 -1.45605200 -2.03844000
C 0.64352200 -3.10625300 -1.85137700
C -1.77757500 -0.52295900 0.75323500
C -1.10836900 0.72381200 0.50354400
C 0.24601200 0.88408400 0.76845100
C -0.96890400 -1.54385500 1.31888000
C 0.38106200 -1.37652900 1.57643400
C 1.02696000 -0.16510300 1.29693700
C 2.47991800 -0.03785200 1.61049200
C 2.81932300 1.32966800 -1.41540600
C 3.01264800 2.76858300 -1.17417200
C 2.52660900 3.68009700 -2.31422200
C 2.54536700 5.08993400 -1.68235500
C 3.51253900 3.59577500 -3.49768500
O -7.35518700 -1.23070100 -2.14313700
O -6.83987200 -3.40431200 -2.43149700
O -7.29058600 -3.47714700 2.91301300
O -6.23346700 -2.19726200 1.39420300
O 2.75669300 2.81251800 2.91035300
O 4.95326600 2.60774700 3.38912000
O 3.69000400 -1.24973900 -2.03732600

O	0.34440700	-4.29135000	-1.57053200
O	-3.03336300	-0.73839000	0.47139300
O	-1.88220700	1.74252400	-0.03175900
O	3.12340500	1.00725600	1.17074100
O	3.02917100	-0.94575700	2.27273300
H	-0.44405300	-6.09970000	-2.78141700
H	-0.96400900	-7.20451100	-1.50844200
H	-2.87342200	-5.65845700	-3.36716700
H	-3.33859900	-6.75296500	-2.05818000
H	-1.69821000	-4.44504600	-1.24046000
H	-3.13250100	-2.54094100	-0.06345300
H	-4.71174000	-3.10325700	0.28588100
H	-4.92305400	-5.66799500	-1.98711300
H	-5.71872100	-4.27525800	-1.43818300
H	1.39528100	4.04289500	4.71856000
H	2.13426900	4.89881300	6.08277800
H	4.40243500	4.00396500	5.43787300
H	3.26185900	2.69524400	5.70608500
H	-1.28780100	8.91548400	-1.16270000
H	-2.72659400	8.92784300	-0.13591600
H	-0.04600400	6.42005600	-0.89200200
H	-3.45472300	4.01735200	-0.53864700
H	-5.52771700	1.88897000	1.64547300
H	-7.00588900	0.95780400	1.91260100
H	-5.86418200	1.72829300	-0.84199800
H	-7.37938400	0.86773800	-0.59595000
H	-6.03505500	-0.96447100	0.58487500
H	-5.91762300	-0.74455600	-1.05419900
H	-5.83499600	-0.33730300	7.30318800
H	-6.23896100	-2.00952000	6.86847200
H	-5.77364000	-2.49354200	4.16064200
H	-2.52289300	0.14347400	3.67266400
H	-2.33793600	-1.42753700	-2.49400800
H	-2.89262400	0.18664600	-2.02078300
H	-2.47405500	1.94011400	-3.48949200
H	-1.97956500	4.77587800	-5.21824800
H	-3.15308500	3.99894600	-4.15128100
H	-2.23894800	5.40832300	-3.59096000
H	1.58768000	5.53107100	-4.16231300
H	-0.02956500	5.63703100	-4.81414700
H	0.27809700	6.20400200	-3.18120200
H	-4.64379300	-0.17750300	-0.02596700
H	-1.32040000	2.51369200	-0.21620600
H	0.70783000	1.85155900	0.59046500
H	-1.45613100	-2.48874900	1.54393300
H	0.96659700	-2.17678600	2.01342000
H	-4.23787000	6.43557800	-0.78514300
H	-4.11570400	-1.45405500	2.51441500
H	2.70071000	-3.48076000	-1.92513800
H	-2.89316300	8.79458700	-1.89543800
H	2.50050000	5.38640200	4.41951300
H	-1.34985400	-7.57942500	-3.20098700
H	-4.72034300	-1.64096800	7.71234500
H	-7.09707600	2.56859000	1.18349100
H	-2.61685100	-0.20770400	-3.76095700
H	3.51079600	0.58270600	-1.07637700
H	2.46776300	3.00166100	-0.24963800
H	4.06487600	2.96080900	-0.95488800
H	2.43549000	5.90143500	-2.39762100
H	1.76016300	5.17405000	-0.92622800
H	3.50792400	5.23402900	-1.18017800
H	3.17650500	4.19053700	-4.35035900
H	4.49521600	3.96992400	-3.19216900
H	3.63490800	2.56499100	-3.83771100

H	-8.27139600	-2.59042700	-4.63366200
H	-9.35344600	-1.66888900	-3.59500100
H	-10.02839800	-3.77474000	-2.40775000
H	-8.83411700	-4.68809500	-3.33961300
H	-8.27244700	-0.67582800	1.50759600
H	-9.29292000	-2.05448500	1.86246700
H	-9.60789800	-0.32992600	3.63255700
H	-7.87094700	-0.37935400	4.01524000
H	2.96571700	2.15034700	2.15523800
H	-8.86712500	-1.80647500	4.30672200
H	-10.26546100	-3.99751400	-4.14716400
H	-9.53599900	-1.68186800	-0.65153700
H	-11.45985800	1.85497200	-2.11469400
H	-13.51148200	1.26546800	-0.85047600
H	-11.57983600	-2.28318600	0.59067900
H	-14.07953200	-1.98162400	0.86439500
H	-3.60870100	6.40289400	4.67065400
H	-4.78328800	4.38812300	5.49807800
H	-4.41288400	2.18237400	4.40039600
H	-2.90371100	2.04353200	2.42090500
H	-1.68110600	4.03067700	1.66116300
H	-1.37090500	1.92314600	6.70258400
H	0.34559900	2.16776900	7.01777500
H	-0.89694500	-0.25827900	5.65807200
H	0.71621900	1.97180700	4.31550400
H	-0.14805200	0.66171900	3.53770200
H	-1.05320200	1.94037800	4.36701700
H	2.04820000	0.53113400	6.00653700
H	1.27303900	-0.92176900	6.65843100
H	1.50076700	-0.73456500	4.90913900
H	1.42475200	-5.86742300	-1.00213000
H	5.32690000	-7.14144100	-1.17193500
H	6.24955700	-5.21974100	3.94364000
H	3.79843800	-4.85752200	4.19452200
H	2.51441500	-3.81743000	2.33645100
H	3.65728500	-3.17595000	0.24703500
H	7.27557200	-3.13186200	-0.21268100
H	5.71289900	-2.79378000	-0.97441000
H	12.77791600	-0.55917600	1.79293300
H	12.65317700	-2.01368300	0.83752100
H	5.77863700	5.25173700	2.43300500
H	5.10525600	4.31513100	1.09189600
H	6.68431300	3.87976500	1.75251400
H	5.06420300	0.58510700	1.31052500
H	4.72533400	-1.04557500	2.70275700
H	5.99858400	-2.11490300	3.16222300
H	8.58671300	-0.69310100	1.21528200
H	8.20750000	-1.79171400	2.37059600
H	6.33392700	2.06067800	0.09601700
H	7.79582700	1.11780500	0.31392200
H	5.57504800	-0.10709100	-1.33098500
H	7.30235100	-0.30307700	-1.54109200
H	7.45791000	2.08562200	-2.38106200
H	5.72854300	2.25490900	-2.17283300
H	4.66970700	-0.24956500	-3.31389100
H	0.54584200	5.10695500	-8.18128700
H	-0.45838900	4.34080200	-6.93969700
H	1.27937400	4.51624900	-6.68711900
H	1.46565300	2.68648600	-5.37024400
H	1.48155300	0.93180700	-5.31978000
H	2.62535700	1.78484800	-6.36689600
H	1.73787200	2.89873700	-8.53965500
H	0.02357100	2.80988200	-8.91618800
H	-0.42309800	1.78994300	-6.69125500

H	-14.85318100	-0.49437200	0.29522000
H	-13.90767800	-0.48851600	1.78653700
H	1.68268200	-6.73677400	1.60153600
H	0.32248000	0.12690100	8.53926900
H	1.73000600	0.32801600	-8.31389300
H	6.30022400	1.90772000	-4.53965600
H	12.90716000	-1.93613000	3.90939300
H	12.65383000	-3.40378800	2.97713000
H	-2.51014800	7.17582900	1.90379400
H	-0.96648300	6.33785400	2.01173700
H	8.78401500	7.09715400	0.57155800
H	7.93896000	6.92204300	2.12919500
H	2.95174300	-6.26217600	-2.68839300
H	6.90033200	-5.61214900	-0.75354000
H	0.00259600	0.35509600	-8.71526100
H	0.56044200	-0.43332500	-7.22984100
H	0.77915100	-8.05216700	0.83295000
H	0.12482300	-6.38920700	0.81317800
H	3.55947600	-7.93671900	-2.78612800
H	7.05007700	-4.56814900	-2.17351000
H	14.16042500	-2.47140500	2.76890000
H	-1.39488600	-0.09687100	8.16257600
H	-0.86110700	1.33325200	9.06127600
H	8.88391800	5.56042500	1.46368700
H	-1.57114900	7.26643400	3.39700200
H	7.17105900	0.43134300	-4.15879900
H	3.72529800	-0.47573500	-5.35146900
H	-8.54956200	-0.20223700	-2.17885200
H	7.38884300	-4.56770800	1.84047800

TS1 (11.6 kcal/mol)

C	0.59693700	0.07311500	7.93165400
C	0.50597600	-1.35591600	7.39230700
C	0.57321600	-2.37001900	8.54759800
C	1.59755200	-1.59251900	6.34741900
C	0.34142700	-3.82326100	8.12841400
C	4.49614500	0.25975700	5.34725500
O	5.01740100	-0.14337100	6.38639000
N	4.95462900	0.05220800	4.10436200
C	6.19903200	-0.65707300	3.93204900
C	6.33430700	-1.31210500	2.55173300
C	6.28787800	-0.38289800	1.32675300
C	6.42032600	-1.22081700	0.04591000
N	5.98344500	-0.57695600	-1.19055200
C	6.81577000	-0.12538000	-2.14832600
N	8.05108100	0.27762200	-1.85809300
N	6.39155900	-0.14900600	-3.42309600
C	6.25838200	5.09381400	1.16126900
C	4.85739500	5.49693400	1.59189100
O	4.04686400	4.69171300	2.04966100
C	6.37587000	3.60047800	0.78308000
C	5.89707200	3.30814300	-0.62181300
C	6.79282700	3.40409000	-1.69550800
C	4.56495600	2.95942700	-0.87933600
C	6.35318500	3.19534700	-3.00310900
C	4.12826700	2.74362900	-2.18681900
C	5.01778300	2.87405400	-3.25448700
N	4.58794700	6.83132200	1.54513600
C	3.28014400	7.35653800	1.91069600
C	2.51820000	7.75597500	0.65440200
O	2.80424100	8.79694000	0.05904300
N	1.57382200	6.88684000	0.23966600
C	0.85560300	7.16258100	-0.98721500
C	12.94337700	2.59525400	-2.97351400
C	11.72203100	3.16510600	-2.23010500
C	10.48550500	2.22685500	-2.07889200
O	10.17480300	1.51484900	-3.08394000
O	9.86664300	2.29759900	-0.98292100
C	8.17558700	-6.17715600	-0.93329900
C	5.75721700	-4.80340600	-1.12261600
S	6.79487900	-5.62830800	0.13342700
C	-1.91829600	-6.51368300	-2.30159300
C	-2.58291600	-5.19497800	-2.60329200
C	-3.92871600	-5.13874200	-2.98184200
C	-1.86417600	-3.99663400	-2.48670900
C	-4.55285700	-3.91572000	-3.23967200
C	-2.48893900	-2.77756500	-2.73840000
C	-3.83186100	-2.72816800	-3.11987600
C	-14.07549400	0.94157100	-0.78958200
C	-12.68851400	0.62777600	-0.28021900
C	-11.72200300	1.63142600	-0.13974200
C	-12.32373100	-0.67193500	0.09093100
C	-10.45042900	1.36284500	0.35858400
C	-11.05779500	-0.96037600	0.59338400
C	-10.10118300	0.05568400	0.74262800
O	-8.89203500	-0.26882600	1.23953700
C	-0.69519900	-0.76640900	-8.18910400
C	-0.04832300	-1.33154500	-6.91617900
C	0.40273300	-0.25906900	-5.90475700
C	0.67448000	-0.86997700	-4.52311400
C	1.64534700	0.50442000	-6.38364200
N	-2.36571500	4.92595700	1.23216900
N	-3.59913400	3.41018200	-0.04796700
N	-4.71197900	4.99729800	1.18189500
N	-3.66099200	-6.30715000	0.46710400
N	-2.46781200	-4.53114900	1.01342200
N	-5.33995800	0.37409400	-0.10788500
N	-3.53543700	0.06025200	-5.89542500
N	-3.53836600	1.04444500	-3.90147400
N	-1.02126600	0.20624100	2.68126400
N	1.50435100	-0.82919300	2.16139200
N	-0.35360100	2.35731500	2.06449300
N	1.94198900	2.80331900	1.79018800
C	-9.66595900	4.06552800	3.24264500
C	-8.83616300	2.77510600	3.15985800
C	-7.63285900	2.82377800	2.18784900
C	-8.83392000	0.94485600	-3.64371000
C	-8.23940300	1.51954000	-2.34190200
C	-6.96716600	2.36082600	-2.57115900
C	-1.42476900	7.04297700	2.24359300
C	-2.13825000	5.69459300	2.46366700
C	-3.54919200	4.43833300	0.80959300
C	2.23904700	-4.49564100	-4.86365100
C	3.58903400	-3.90606700	-5.32320500
C	4.07558900	-2.80931200	-4.38900300
C	-2.32914700	-8.26604100	1.38052000
C	-2.56204000	-6.79821300	1.16139200
C	-3.55116900	-4.94897600	0.39428700
C	-1.84298300	-5.67100500	1.49245000
C	-6.61018700	-1.55315100	-1.14239500
C	-5.40880500	-1.10295800	-0.32050000
C	-5.53844200	1.17451500	-6.96647200
C	-4.57774000	0.97425400	-5.83272300
C	-2.93661400	0.13189800	-4.72006600
C	-4.58982900	1.59720000	-4.60612800
C	-2.40551300	0.64751900	2.74282300
C	-0.69998100	-1.08915400	3.11085900

C	-1.63666700	-1.88971700	3.76369400
C	-1.32556200	-3.18554800	4.16911800
C	-2.40049400	-3.98748900	4.86575100
C	-0.04967600	-3.72827900	3.90805900
C	0.29209700	-5.06410300	4.53809900
C	0.87878500	-2.97793200	3.15357500
C	0.55975700	-1.64628700	2.82229500
C	-0.01483800	1.08615900	2.31150900
C	1.28466800	0.57780900	2.19398400
C	2.34953900	1.48999300	1.99300600
C	0.62800100	3.22160900	1.70959600
C	-1.36074900	0.22727400	-0.48413300
C	-1.28095900	-1.17060800	-0.06045400
C	-0.12492900	-1.88153800	-0.20141800
C	-0.11514100	0.82966000	-0.88722600
C	1.06519700	0.13682200	-0.90289500
C	1.13587200	-1.26665400	-0.55781300
C	2.08788000	-2.12064900	-1.39766600
C	2.23140600	-1.37370200	1.12817000
C	2.57900800	-2.83849600	1.30498300
C	2.25851300	-3.47449800	2.68478100
C	2.35379900	-4.99168000	2.40855500
C	3.32829500	-3.09036300	3.72421900
O	-7.32191100	1.73093300	1.60062400
O	-7.02239800	3.91109800	2.08836900
O	-7.00355400	3.22223700	-3.46011100
O	-5.92922900	2.12366000	-1.82750900
O	4.29604600	-3.23664700	-3.16588800
O	4.22844100	-1.63946200	-4.74834300
O	3.57606400	1.22192600	1.94797300
O	0.38568800	4.39836700	1.32712400
O	-2.46303000	0.86519100	-0.50273900
O	-2.43122800	-1.71313400	0.42484500
O	3.27323500	-1.63272100	-1.59137600
O	1.69475800	-3.23246100	-1.77906400
H	-0.44521800	6.88449600	1.79241300
H	-2.00883500	7.69910900	1.59017200
H	-1.52212000	5.06625300	3.11395300
H	-3.09476400	5.84333800	2.97252500
H	-1.49857200	4.47664100	0.92077800
H	-2.84724400	2.72594400	-0.16299900
H	-4.45721900	3.22607400	-0.57184000
H	-4.67524600	5.83510900	1.73822100
H	-5.57385400	4.42203000	1.31090100
H	1.43128200	-3.77338400	-5.00474000
H	1.99708500	-5.39388800	-5.44145200
H	4.34991700	-4.69714000	-5.31560200
H	3.52837500	-3.50039900	-6.33636800
H	-1.41666600	-8.40711500	1.96389900
H	-2.20497000	-8.79886600	0.43021600
H	-0.91748800	-5.60389600	2.04145200
H	-4.25311300	-4.32875100	-0.14271400
H	-6.61333300	-1.07182600	-2.12453400
H	-7.54895200	-1.32406300	-0.63027500
H	-4.47554900	-1.38193000	-0.80788500
H	-5.40484900	-1.58661000	0.66123500
H	-5.68786900	1.02144500	-0.96689600
H	-5.91848900	0.72866000	0.67132300
H	-6.02104100	0.22994700	-7.24418300
H	-6.31619900	1.88664100	-6.67760200
H	-5.22765300	2.36412700	-4.19207500
H	-2.07881500	-0.44425100	-4.41353300
H	-2.47484100	1.59502800	2.21773900
H	-3.03422700	-0.09258700	2.24528500
H	-2.62876400	-1.50564900	3.95808200
H	-2.16274000	-4.18442600	5.91836700
H	-3.35045600	-3.44804000	4.84136600
H	-2.55803800	-4.95548300	4.37813400
H	1.30721300	-5.06630700	4.93881800
H	-0.37546800	-5.26864500	5.37485200
H	0.21256700	-5.91417700	3.85201600
H	-4.34709200	0.61219800	0.04167700
H	-2.27212700	-2.64501200	0.70635200
H	-0.13481600	-2.94623700	0.00195600
H	-0.15170300	1.87769200	-1.16832100
H	1.98485600	0.61564400	-1.22534200
H	-4.36359400	-6.85918200	0.00074100
H	-3.30941900	1.23601800	-2.93336000
H	2.68184100	3.50239700	1.71759400
H	-3.15527800	-8.73608700	1.92696700
H	2.27561300	-4.73637400	-3.79981100
H	-1.28714300	7.54329700	3.20787100
H	-5.03465600	1.55421500	-7.86410400
H	-6.54947100	-2.63472300	-1.29819300
H	-2.73439700	0.78845100	3.77966200
H	3.03109100	-0.73095600	0.78615700
H	2.07401100	-3.43221300	0.54005200
H	3.64897500	-2.96166800	1.11116400
H	2.44707500	-5.59166300	3.31230900
H	1.49479700	-5.34775600	1.83160700
H	3.25049300	-5.17909800	1.80856000
H	3.10195800	-3.52611400	4.70069500
H	4.30550900	-3.47202700	3.41088900
H	3.40615600	-2.00908700	3.84563900
H	-8.40585900	2.55640900	4.14764400
H	-9.46911500	1.92026600	2.90903600
H	-10.19672800	4.24598000	2.30187000
H	-9.00908000	4.91976600	3.42355900
H	-8.06379400	0.73345200	-1.60482700
H	-8.97664900	2.19481900	-1.89309300
H	-9.79363000	0.45407900	-3.45160700
H	-8.15532600	0.21314300	-4.09713100
H	3.94248100	-2.51283200	-2.45684300
H	-8.97594900	1.75506400	-4.36290000
H	-10.40979300	4.00546800	4.04432800
H	-9.70996600	2.14983900	0.42933500
H	-10.78441100	-1.97158000	0.87840500
H	-13.04699200	-1.47816600	-0.01655700
H	-11.96307900	2.64991400	-0.43661400
H	-14.72919600	1.32348200	0.00661000
H	-4.49321700	-6.06469700	-3.07886600
H	-5.59585400	-3.88937800	-3.54190100
H	-4.30205700	-1.77501500	-3.33405800
H	-1.91998000	-1.86332600	-2.62109900
H	-0.81669000	-4.00535600	-2.19360000
H	-0.77939900	-1.98696800	-6.42631200
H	0.81377500	-1.96623000	-7.17094000
H	-0.42499700	0.45628700	-5.80853100
H	1.58298800	-1.47675500	-4.53554100
H	0.82024000	-0.09446500	-3.76456600
H	-0.14696800	-1.51219500	-4.18853900
H	2.51110600	-0.16609300	-6.43033400
H	1.49684100	0.95500800	-7.37085300
H	1.89776800	1.31092500	-5.68580200
H	1.41105000	5.99069000	0.70547400
H	5.19242900	7.43598800	1.00788000
H	7.06906500	3.25838900	-3.81860900
H	4.67538100	2.69732900	-4.27026900

H	3.09405800	2.46707900	-2.37235200
H	3.88374000	2.82381000	-0.04913900
H	7.42831700	3.31015700	0.86347500
H	5.79972200	3.01712300	1.50696500
H	11.99958000	3.50416300	-1.22702200
H	11.36515400	4.05355100	-2.77150800
H	5.45686300	-5.49595700	-1.91059100
H	4.85294300	-4.44220900	-0.63002000
H	6.27026000	-3.95134000	-1.57015100
H	5.00065600	-0.70118200	-1.43046800
H	5.52836900	-0.61899400	-3.71290000
H	6.82499800	0.48671300	-4.07246000
H	8.25252500	0.72026200	-0.96676400
H	8.74623600	0.63938100	-2.55748400
H	5.80050800	-2.11779800	0.15180000
H	7.45194500	-1.56038700	-0.09062200
H	5.34296600	0.16308500	1.29363200
H	7.08785900	0.36701300	1.37697700
H	7.27844100	-1.87334600	2.54585600
H	5.54061800	-2.05812300	2.44122400
H	4.45595800	0.46539000	3.30715800
H	0.32984300	-4.49265500	8.99566600
H	-0.61857500	-3.92992400	7.61066200
H	1.12037100	-4.17714100	7.44775500
H	1.52716300	-2.59028000	5.91533800
H	1.49945900	-0.88533500	5.51857500
H	2.59753800	-1.46432600	6.77738700
H	1.55257800	-2.28049300	9.03858300
H	-0.17446300	-2.09252900	9.30351200
H	-0.46815400	-1.47856900	6.89534200
H	-14.55812100	0.05056900	-1.20457500
H	-14.05026300	1.70363300	-1.57598000
H	1.48704500	7.00883000	-1.87005200
H	-0.02940500	-0.07042600	-8.71183900
H	1.56338500	0.23837400	8.42272800
H	6.25744300	-1.41956700	4.71371500
H	13.39830900	1.77191300	-2.41091500
H	12.62996500	2.19622800	-3.94250000
H	-2.64256400	-7.33493000	-2.28631500
H	-1.41226400	-6.48399700	-1.33207700
H	8.89640000	-6.69300700	-0.29521200
H	7.82340100	-6.86695500	-1.70548400
H	2.77341200	6.57709500	2.48083100
H	6.60378800	5.72969100	0.33810300
H	-0.19366500	0.28295900	8.66189100
H	0.51068100	0.80494700	7.12175500
H	0.51577700	8.20089300	-0.99462100
H	-0.00488900	6.49382000	-1.04414800
H	3.40159500	8.24957200	2.53043900
H	6.91163900	5.30643900	2.01853800
H	13.71168200	3.36115500	-3.13345800
H	-1.61928500	-0.24087100	-7.92768100
H	-0.95070200	-1.56918600	-8.89027000
H	8.66639400	-5.32164100	-1.40514600
H	-1.15776100	-6.75613700	-3.05328100
H	7.04919700	0.02274900	4.09862200
H	3.54951000	0.83527600	5.35129400
H	-8.32101800	0.55595000	1.40738400
H	7.84691600	3.58151100	-1.49881800

Int1 (7.6 kcal/mol)

C	0.39722600	0.17707500	7.94928900
C	0.33688400	-1.24078700	7.37549500
C	0.36743200	-2.27787900	8.51181000
C	1.46759100	-1.45062600	6.36679300
C	0.17834000	-3.72540900	8.05345000
C	4.37373600	0.38880100	5.48738200
O	4.87960900	0.02183200	6.54756400
N	4.86785300	0.16628300	4.26061000
C	6.13219500	-0.51977900	4.13654600
C	6.30097100	-1.22585400	2.78808400
C	6.22074100	-0.35323700	1.52515600
C	6.27126300	-1.26874300	0.29525100
N	5.98259900	-0.65081900	-0.99367300
C	6.93730600	-0.26745200	-1.86238800
N	8.16691300	0.04740500	-1.45468800
N	6.66270900	-0.26213300	-3.17337600
C	6.20575800	5.20320100	1.30875800
C	4.78328200	5.59401300	1.68582800
O	3.95995500	4.78179700	2.10425700
C	6.36983200	3.68879400	1.05470500
C	6.03286200	3.29473900	-0.36529500
C	7.03863500	3.23867400	-1.33969900
C	4.71177800	3.01247900	-0.73777400
C	6.72534200	2.94702400	-2.66757400
C	4.39908300	2.71503800	-2.06420100
C	5.40229600	2.69611800	-3.03483600
N	4.50811700	6.92846700	1.63830600
C	3.17654800	7.43408600	1.94004000
C	2.46093200	7.79295100	0.64460400
O	2.80492600	8.79103700	0.00637700
N	1.49554500	6.94292000	0.24271200
C	0.84777400	7.17916400	-1.03077300
C	13.04989800	2.75107400	-2.58651200
C	11.76413500	2.49305900	-3.38454500
C	10.58284800	1.92146200	-2.55433100
O	9.74262000	1.22218800	-3.22609700
O	10.51940100	2.19185600	-1.33786800
C	8.33253400	-6.06220800	-0.60601100
C	5.92919700	-4.67893800	-0.92826600
S	6.92158400	-5.46116400	0.39004000
C	-1.70781500	-6.54482400	-2.28870500
C	-2.36139000	-5.24451300	-2.68186400
C	-3.66433100	-5.22042000	-3.19128600
C	-1.67259500	-4.03260700	-2.53211500
C	-4.27216200	-4.01503700	-3.55040100
C	-2.28271300	-2.83147300	-2.88354000
C	-3.57983400	-2.81352900	-3.40048300
C	-14.00125600	0.76507400	-1.23942300
C	-12.74336100	0.50614300	-0.43616500
C	-11.64166300	1.36939800	-0.49123000
C	-12.62463000	-0.63031400	0.37521400
C	-10.47120800	1.12190500	0.22639500
C	-11.46556300	-0.89536100	1.09852800
C	-10.36851300	-0.02069500	1.03746100
O	-9.27433100	-0.32109700	1.76890200
C	-0.37229800	-0.84170500	-8.19450600
C	0.25690000	-1.31373800	-6.87680600
C	0.65854300	-0.17033200	-5.92449000
C	0.92119500	-0.69425100	-4.50740700
C	1.88926700	0.59771200	-6.42625400
N	-2.63007500	5.07356400	1.06792300
N	-3.57709300	3.29801600	-0.15802300
N	-4.94375700	4.87281800	0.77585000
N	-3.58583600	-6.40619700	0.34955800
N	-2.52735300	-4.55662800	0.92286300
N	-5.56289600	0.31197300	-0.05706000

N	-3.24666800	0.08694000	-5.98505400
N	-3.45761200	0.99009100	-3.96419500
N	-1.06791400	0.23375900	2.63327800
N	1.52230400	-0.75336600	2.27007600
N	-0.45473800	2.37586900	1.96359200
N	1.82828600	2.89615700	1.77162500
C	-9.76211300	3.98706700	2.89704700
C	-8.85640400	2.77230600	3.10768200
C	-7.71845300	2.66492100	2.07490900
C	-8.67231400	0.80839500	-3.92653400
C	-8.26909900	1.45355400	-2.58324800
C	-6.97241900	2.28899400	-2.68161600
C	-1.53216100	7.06218000	2.12751000
C	-2.79340400	6.20479200	1.98929700
C	-3.69730700	4.40891800	0.57997200
C	2.50234600	-4.49828500	-4.73932300
C	3.77552600	-3.77767000	-5.20991900
C	4.22361100	-2.72474100	-4.21267000
C	-2.21273900	-8.26529800	1.39676600
C	-2.51156700	-6.81888800	1.12851600
C	-3.54258800	-5.04675000	0.24417800
C	-1.87708000	-5.64698500	1.47566000
C	-6.49719400	-1.63491000	-1.33023500
C	-6.28685100	-0.98799500	0.03126300
C	-5.27638100	1.04782400	-7.14585100
C	-4.37591100	0.89236000	-5.95568900
C	-2.72430200	0.17248400	-4.77423100
C	-4.51896900	1.46326800	-4.71103000
C	-2.44633100	0.65975200	2.66253200
C	-0.74296100	-1.04985700	3.09678200
C	-1.69786700	-1.86039500	3.70718300
C	-1.39157700	-3.15763100	4.11817600
C	-2.48387600	-3.96877000	4.77749300
C	-0.10598300	-3.68678200	3.88814700
C	0.22885800	-5.02748300	4.51402900
C	0.84312900	-2.91530500	3.17721200
C	0.54136000	-1.57478900	2.86346200
C	-0.07507100	1.12351100	2.26003100
C	1.23565700	0.64474300	2.20168700
C	2.27528600	1.59624700	2.03770400
C	0.50352900	3.27698500	1.65821100
C	-1.42410600	0.13503900	-0.59698100
C	-1.33121300	-1.24187400	-0.08196400
C	-0.13462900	-1.86856900	0.01843200
C	-0.16750100	0.78837800	-0.91214900
C	1.03211400	0.20333400	-0.67611600
C	1.17669600	-1.20458300	-0.22227500
C	2.01195100	-1.95893200	-1.34433700
C	2.13299400	-1.31482700	1.07566400
C	2.54778700	-2.77691200	1.32753400
C	2.22792300	-3.39751800	2.71930800
C	2.35832600	-4.91679400	2.47195900
C	3.28051600	-2.98251200	3.76398600
O	-7.42335800	1.48901500	1.66237500
O	-7.14149000	3.72214200	1.74548000
O	-6.92559900	3.15301100	-3.56652800
O	-6.00931800	2.03192100	-1.85163600
O	4.31497700	-3.17754000	-2.98047000
O	4.45661800	-1.55983700	-4.53961100
O	3.51049000	1.37575300	2.06814700
O	0.22223000	4.45552200	1.29077200
O	-2.53472000	0.70418200	-0.76158600
O	-2.52245900	-1.79881800	0.25937800
O	3.23925500	-1.57523600	-1.43319500
O	1.46469900	-2.85144000	-1.99180500
H	-0.69017500	6.45769500	2.47066200
H	-1.25377500	7.50631600	1.16873800
H	-3.10826100	5.84293100	2.97881400
H	-3.59183300	6.85096800	1.60750300
H	-1.68257700	4.66399700	1.01878400
H	-2.72890100	2.74747000	-0.23524200
H	-4.37559800	2.98783500	-0.71680500
H	-5.06210700	5.63817300	1.41699600
H	-5.74010900	4.20900300	0.83248900
H	1.68151500	-3.79217100	-4.59764200
H	2.19462900	-5.25171000	-5.47238300
H	4.59316200	-4.50301800	-5.32092100
H	3.63366300	-3.28672400	-6.17603400
H	-1.33554700	-8.34238400	2.04287500
H	-1.99482300	-8.80821400	0.46922500
H	-0.99870800	-5.51221800	2.08592700
H	-4.23473600	-4.47767100	-0.35822500
H	-5.54314400	-1.90700700	-1.79012700
H	-7.02828900	-0.96835200	-2.01159600
H	-5.71621500	-1.64858200	0.69122300
H	-7.24576600	-0.78304600	0.50708700
H	-5.88460900	0.96244600	-0.91650400
H	-5.84228700	0.88395700	0.75854700
H	-5.67015100	0.07777500	-7.47203000
H	-6.12111000	1.69699800	-6.89932700
H	-5.23560800	2.16994700	-4.31843500
H	-1.83168000	-0.32869400	-4.43520000
H	-2.51703500	1.61348900	2.14937800
H	-3.06665800	-0.07881600	2.14770200
H	-2.70212000	-1.48925100	3.86079000
H	-2.28036800	-4.16908500	5.83686500
H	-3.43492500	-3.43266900	4.72574100
H	-2.62492800	-4.93613500	4.28299600
H	1.21850600	-5.01200800	4.97501200
H	-0.48214100	-5.26321400	5.30562800
H	0.21490600	-5.86825600	3.81210500
H	-4.54110000	0.19663000	-0.12743800
H	-2.37956900	-2.71523800	0.60161600
H	-0.12127500	-2.90909000	0.32164400
H	-0.23232200	1.80382600	-1.29019100
H	1.95573600	0.73314200	-0.89043600
H	-4.22723100	-7.00844900	-0.14204600
H	-3.29672100	1.16093600	-2.97782000
H	2.55006800	3.61485600	1.73099400
H	-3.04615100	-8.77182500	1.89777700
H	2.67490200	-4.97859100	-3.77521500
H	-1.72047200	7.86475400	2.84612900
H	-4.74327000	1.48207000	-8.00038300
H	-7.09055000	-2.54729800	-1.21356500
H	-2.80803100	0.78586400	3.69139900
H	3.00608200	-0.71120900	0.83992000
H	2.07929400	-3.41445100	0.57347400
H	3.62466300	-2.87337900	1.16478300
H	2.45286600	-5.49821600	3.38797100
H	1.51511600	-5.30411500	1.89108700
H	3.26786400	-5.09503200	1.88806600
H	3.04219400	-3.40463100	4.74416700
H	4.26510800	-3.36329500	3.47386600
H	3.34001100	-1.89857100	3.86375000
H	-8.36645600	2.84502800	4.08886200
H	-9.43455100	1.84531000	3.11802800
H	-10.33685700	3.88307000	1.97082800
H	-9.15659100	4.89303100	2.81652900

H	-8.20112700	0.70486000	-1.78957300
H	-9.06194300	2.14755200	-2.27931300
H	-9.64218800	0.30670700	-3.84631800
H	-7.92668900	0.07296600	-4.24947300
H	3.93115500	-2.45196100	-2.30338000
H	-8.72611400	1.58384700	-4.69438800
H	-10.47330900	4.10304500	3.72227800
H	-9.62782500	1.79846100	0.15314700
H	-11.38746900	-1.77514200	1.72971600
H	-13.46124100	-1.32320100	0.44520700
H	-11.69200000	2.25966200	-1.11420100
H	-14.13952600	1.83453900	-1.42801300
H	-4.20643300	-6.15678400	-3.31369300
H	-5.28218500	-4.01343300	-3.95077400
H	-4.03570500	-1.87485900	-3.69484600
H	-1.73540100	-1.90780700	-2.74166300
H	-0.65866400	-4.00863400	-2.14169600
H	-0.46976000	-1.95537500	-6.36291900
H	1.13929500	-1.94159500	-7.07216300
H	-0.19031500	0.52556300	-5.88657100
H	1.83544700	-1.29088400	-4.47255200
H	1.05053700	0.12656800	-3.79387200
H	0.10767200	-1.32779500	-4.14113100
H	2.77277500	-0.05134900	-6.41759600
H	1.75046000	0.98475600	-7.44143100
H	2.10770500	1.45079000	-5.77392700
H	1.28174600	6.07283300	0.73651100
H	5.11612100	7.53617600	1.10792600
H	7.53227700	2.86340300	-3.38931200
H	5.15595200	2.44838000	-4.06325500
H	3.37283300	2.48649400	-2.33921900
H	3.94132200	2.99547100	0.02267300
H	7.40926000	3.41765000	1.26522700
H	5.72713100	3.15131800	1.75801600
H	11.41083900	3.43724200	-3.82388000
H	11.94534600	1.81784900	-4.22752500
H	5.66165800	-5.39641200	-1.70611300
H	5.00739900	-4.31227300	-0.47468600
H	6.45063900	-3.83289300	-1.37811100
H	5.02173000	-0.72044800	-1.31869200
H	5.79242400	-0.64352900	-3.55441800
H	7.29400300	0.24685800	-3.77387600
H	8.25889600	0.40335400	-0.51478700
H	8.84122500	0.50050900	-2.16240600
H	5.52161200	-2.05460900	0.42240500
H	7.24464700	-1.76800800	0.23036500
H	5.29212500	0.22199900	1.49839800
H	7.04465500	0.37274100	1.50410400
H	7.26577000	-1.75038600	2.80471000
H	5.53728700	-2.00386800	2.70827000
H	4.37504300	0.55171800	3.44574300
H	0.12994300	-4.41165500	8.90627700
H	-0.75022100	-3.83403600	7.48219600
H	0.99734500	-4.05544500	7.40897500
H	1.41405300	-2.43678300	5.90649400
H	1.40108700	-0.72368900	5.55226600
H	2.45018500	-1.33408200	6.83822400
H	1.32192700	-2.18413500	9.04915500
H	-0.41811900	-2.02695700	9.23819900
H	-0.61707900	-1.35891800	6.84001800
H	-14.89100100	0.39846600	-0.71613700
H	-13.97143500	0.26600600	-2.21712400
H	1.54571500	7.05501000	-1.86642700
H	0.29267400	-0.16953600	-8.74838800
H	1.34451100	0.33834100	8.47777600
H	6.19516200	-1.24637000	4.95096700
H	12.81594800	3.34886200	-1.70182200
H	13.48771600	1.81040500	-2.23431100
H	-2.42628300	-7.37129100	-2.28228400
H	-1.25742800	-6.47371300	-1.29443000
H	9.02702700	-6.55520700	0.07768400
H	8.00171000	-6.78205400	-1.36006100
H	2.66170000	6.65855200	2.50795600
H	6.54073900	5.78251000	0.44070200
H	-0.42035600	0.36472700	8.65562800
H	0.33381500	0.92598900	7.15302600
H	0.45497600	8.19893100	-1.07542000
H	0.02937200	6.46552100	-1.13910000
H	3.25806400	8.34179200	2.54519100
H	6.83732300	5.51040300	2.15351200
H	13.80375700	3.27300600	-3.18816000
H	-1.30889200	-0.31523400	-7.98350900
H	-0.60220300	-1.69110100	-8.84809000
H	8.84603300	-5.23045100	-1.09587400
H	-0.90512600	-6.81119000	-2.98698600
H	6.96308500	0.18760500	4.28737200
H	3.41507200	0.94195600	5.45550200
H	-8.57601200	0.41503200	1.73572700
H	8.08171700	3.37461000	-1.06302800

TS2 (19.9 kcal/mol)

C	-0.48560900	-0.29908100	-7.95286100
C	-0.46967100	-1.67762800	-7.28363500
C	-0.45534400	-2.78674200	-8.35001700
C	-1.65043600	-1.81326400	-6.31933900
C	-0.29192100	-4.20203300	-7.79141400
C	-4.43633600	0.05809800	-5.46651900
O	-4.96948700	-0.34432900	-6.50083000
N	-4.90801300	-0.11398000	-4.22279200
C	-6.18169100	-0.76970200	-4.04815000
C	-6.33819800	-1.40454600	-2.66317700
C	-6.21181200	-0.48115300	-1.43729600
C	-6.19959400	-1.37597500	-0.18988000
N	-6.09787700	-0.74522900	1.12514000
C	-7.17567700	-0.34469400	1.83887300
N	-8.36873500	-0.16459100	1.28046600
N	-7.06820400	-0.21780600	3.16856100
C	-6.22073500	5.10460600	-1.54925700
C	-4.81398500	5.47284700	-2.00046600
O	-4.02137600	4.64154800	-2.43782100
C	-6.40818600	3.58798900	-1.31495400
C	-6.05881000	3.15695600	0.09222100
C	-7.04274700	3.14626200	1.09196800
C	-4.74884200	2.79076700	0.42747000
C	-6.71231900	2.82619500	2.40807800
C	-4.42108500	2.45637500	1.74289200
C	-5.39756400	2.49004700	2.73971200
N	-4.52544600	6.80531100	-1.99252100
C	-3.19606300	7.29144000	-2.33705000
C	-2.47470100	7.71207100	-1.06388100
O	-2.83715400	8.72636500	-0.46076500
N	-1.49148300	6.89727000	-0.63667500
C	-0.83708700	7.20191800	0.61948800
C	-13.02674600	2.88790800	2.54863800
C	-11.74511700	2.51815400	3.30883800
C	-10.64951700	1.85717900	2.43506300
O	-9.85938600	1.05740600	3.06259800

O	-10.59390200	2.13938600	1.22386400
C	-8.33831100	-6.03070200	1.02338900
C	-5.92524700	-4.66515300	1.20474400
S	-6.93256800	-5.54152700	-0.03672300
C	1.71829800	-6.43251100	2.62804500
C	2.39593600	-5.13261000	2.98175600
C	3.67479200	-5.11511100	3.54900300
C	1.75410500	-3.91092200	2.73974600
C	4.29838400	-3.90719300	3.87178900
C	2.37294400	-2.70666700	3.06150000
C	3.64655600	-2.69719000	3.63424500
C	14.00721400	0.78727800	1.04004200
C	12.72081300	0.48912200	0.29818400
C	11.64661000	1.38924200	0.29713500
C	12.54488200	-0.71507300	-0.39528400
C	10.45090000	1.11308400	-0.36413100
C	11.35833800	-1.01024000	-1.06133100
C	10.28991100	-0.09865000	-1.05988500
O	9.17147400	-0.42565500	-1.73686100
C	0.44907700	-0.40133300	8.21405300
C	-0.46081700	-0.47428100	6.98138600
C	-0.59464000	0.84937000	6.20493000
C	-1.26558700	0.62288600	4.84336300
C	-1.35900700	1.90929200	7.00762300
N	2.60670600	4.97727200	-1.35629800
N	3.57772100	3.28695200	-0.01910000
N	4.92365000	4.79205000	-1.08729000
N	3.61390900	-6.50684300	0.02023100
N	2.64167500	-4.64622100	-0.66325700
N	5.46906000	0.29770200	0.06649200
N	3.26188900	0.34895600	6.04179900
N	3.40523600	1.00228900	3.91571800
N	1.02098400	0.11316900	-2.84167700
N	-1.57292200	-0.85812000	-2.37684900
N	0.41798600	2.28414200	-2.26368400
N	-1.86248800	2.80571600	-2.06371900
C	9.72878400	3.77587000	-3.22906800
C	8.81926100	2.54772400	-3.30731300
C	7.68669100	2.54542100	-2.25956400
C	8.70624000	0.99115300	3.77468500
C	8.29410800	1.60960300	2.42519700
C	6.99186000	2.43912400	2.51385600
C	1.51011000	6.90228700	-2.55122100
C	2.76659000	6.04460500	-2.35383400
C	3.68159800	4.34197700	-0.83682900
C	-2.46460100	-4.24384800	5.00140400
C	-3.11703900	-2.91835300	5.39678500
C	-4.05520700	-2.30582600	4.38240300
C	2.18363600	-8.36006400	-0.95880900
C	2.53687000	-6.91463200	-0.75753100
C	3.62455600	-5.14228200	0.05682900
C	1.95744300	-5.73613600	-1.17403500
C	6.50221500	-1.59219200	1.34359300
C	6.14913700	-1.02369800	-0.02401800
C	5.34382700	1.41803300	7.00974300
C	4.39681800	1.12996300	5.87742500
C	2.69792000	0.29916700	4.84450800
C	4.49615200	1.54427300	4.56577700
C	2.41264900	0.47842900	-2.73135400
C	0.69545600	-1.20329500	-3.20094800
C	1.65401000	-2.05875900	-3.74123800
C	1.35822900	-3.39445800	-4.02243400
C	2.44728000	-4.25486700	-4.62227500
C	0.08732100	-3.91475100	-3.71188900
C	-0.24562800	-5.31545200	-4.18966900
C	-0.85808200	-3.08350800	-3.06305200
C	-0.58658900	-1.71022600	-2.92055500
C	0.03413600	1.01863800	-2.49664200
C	-1.28126900	0.54441900	-2.43778200
C	-2.30974000	1.49374900	-2.25699100
C	-0.53672500	3.19257000	-1.97983500
C	1.65621400	0.14805000	0.75882600
C	1.54861100	-1.19993300	0.22189100
C	0.32669300	-1.74760700	-0.09292600
C	0.42394700	0.83872500	0.94287200
C	-0.78393700	0.31860700	0.51408500
C	-0.89503100	-1.00412700	0.00083400
C	-2.04994600	-1.55184200	1.84920500
C	-2.00013300	-1.30935300	-1.01394200
C	-2.39422700	-2.79631300	-1.08964900
C	-2.18782800	-3.54471600	-2.44667200
C	-2.26083400	-5.03560300	-2.05262400
C	-3.33557000	-3.24394500	-3.42700800
O	7.37587000	1.41687800	-1.74420300
O	7.12830800	3.63798400	-2.02215400
O	6.91442100	3.27405100	3.42729700
O	6.07039700	2.20424400	1.63878300
O	-4.29497400	-3.09275900	3.32059400
O	-4.55542700	-1.19873000	4.50378200
O	-3.54896400	1.26673800	-2.19743500
O	-0.25781900	4.38558100	-1.65349700
O	2.79835200	0.67435300	1.03746700
O	2.72949300	-1.84789400	0.00333700
O	-3.17792000	-1.21979700	1.58284900
O	-1.27810300	-2.06553100	2.59974100
H	0.66502400	6.28136500	-2.85401200
H	1.23569200	7.41307400	-1.62484200
H	3.07508500	5.61566600	-3.31845600
H	3.57039900	6.70922300	-2.01838000
H	1.66560300	4.55746600	-1.30328300
H	2.72490800	2.76836400	0.14971000
H	4.38584900	3.01505800	0.54961700
H	5.02935700	5.48325200	-1.80988700
H	5.72685900	4.13316000	-1.09353000
H	-1.87065700	-4.11152200	4.09500700
H	-1.80117900	-4.58353900	5.80200100
H	-3.68104200	-3.00480200	6.33382800
H	-2.35421900	-2.15612500	5.58030000
H	1.31463300	-8.43445900	-1.61633800
H	1.92941600	-8.84732200	-0.00968500
H	1.09892300	-5.59539000	-1.81104100
H	4.33072400	-4.57176800	0.64152500
H	5.60187300	-1.76812800	1.93894100
H	7.15863700	-0.91853300	1.89769600
H	5.48053900	-1.70158600	-0.56216900
H	7.05338000	-0.88216500	-0.61610400
H	5.88978300	0.99484200	0.81310700
H	5.64127000	0.80941700	-0.80856200
H	5.75158900	0.49086400	7.43053400
H	6.17612500	2.03132400	6.65368100
H	5.19959700	2.20189700	4.07584100
H	1.79276700	-0.23549300	4.59803700
H	2.47051500	1.44937000	-2.24812700
H	2.94365300	-0.26397600	-2.12701600
H	2.65393800	-1.69335300	-3.93569500
H	2.23024800	-4.55315800	-5.65590300
H	3.39466000	-3.71007100	-4.63468000
H	2.60403800	-5.17246800	-4.04465500

H	-1.26304600	-5.36553900	-4.58191300
H	0.41968400	-5.60250200	-5.00449700
H	-0.15935700	-6.09150700	-3.42020700
H	4.45283200	0.22222600	0.29029300
H	2.55317600	-2.74500200	-0.36146700
H	0.31230000	-2.75203000	-0.50420400
H	0.47434400	1.83792500	1.36611500
H	-1.68121800	0.92952500	0.56194700
H	4.22580800	-7.10921400	0.54791800
H	3.20732500	1.04574200	2.90961800
H	-2.58369700	3.52316800	-2.02311600
H	3.00343500	-8.92423900	-1.41916700
H	-3.21064000	-5.02161100	4.81594500
H	1.70903500	7.65274800	-3.32167100
H	4.84380600	1.95106600	7.82776500
H	7.02195700	-2.54776600	1.21943500
H	2.89302300	0.55163900	-3.71563700
H	-2.86997300	-0.70291300	-0.76685400
H	-1.83315700	-3.34212800	-0.32551200
H	-3.45059500	-2.90542400	-0.82122800
H	-2.41294400	-5.70198500	-2.90115300
H	-1.36591900	-5.35448300	-1.50886800
H	-3.11823400	-5.17452800	-1.38410300
H	-3.16887200	-3.75081400	-4.38248300
H	-4.28268000	-3.61502100	-3.02019900
H	-3.42398100	-2.17435000	-3.61752000
H	8.32182900	2.52131300	-4.28726800
H	9.39627600	1.62334600	-3.22944700
H	10.31137800	3.76506100	-2.30183300
H	9.12477700	4.68640300	-3.23468100
H	8.22082100	0.84855600	1.64358300
H	9.08283100	2.29991700	2.10067000
H	9.68021200	0.49509500	3.70309600
H	7.96707800	0.25495500	4.11114400
H	-4.70088200	-2.53144200	2.63551100
H	8.75303700	1.77923800	4.53016100
H	10.43313000	3.80609300	-4.06795200
H	9.63052800	1.82033900	-0.33506100
H	11.23673500	-1.94379200	-1.60203900
H	13.35793800	-1.43864700	-0.41822900
H	11.74037900	2.33303500	0.83009600
H	14.26305500	1.85098600	0.98636700
H	4.18582000	-6.05684400	3.74266000
H	5.29159500	-3.91044700	4.31215600
H	4.11622800	-1.75581500	3.89644800
H	1.85771400	-1.77842100	2.84923700
H	0.76884400	-3.88991500	2.28334500
H	-0.06049600	-1.23719600	6.30025500
H	-1.46646200	-0.81581700	7.27176100
H	0.42120100	1.22388700	6.02698000
H	-2.29660700	0.26592900	4.96158200
H	-1.30541200	1.55182100	4.26346100
H	-0.72526000	-0.11499400	4.24273800
H	-2.38290000	1.57250300	7.21600400
H	-0.87492500	2.12810400	7.96457200
H	-1.42723100	2.84893000	6.44877800
H	-1.26248900	6.01244900	-1.10009700
H	-5.10333100	7.42908700	-1.44669600
H	-7.49938700	2.79583400	3.15522800
H	-5.13963700	2.22316400	3.76062500
H	-3.40482200	2.16013500	1.98802300
H	-4.00456100	2.72751600	-0.35570700
H	-7.45587500	3.34014200	-1.51401000
H	-5.78581800	3.05072300	-2.03624500
H	-11.30045500	3.42621900	3.74085800
H	-11.95734700	1.85374900	4.15304600
H	-5.64931800	-5.32175100	2.03258600
H	-5.01192400	-4.33211400	0.70783900
H	-6.45862100	-3.79325000	1.59150700
H	-5.17485100	-0.49131200	1.46023000
H	-6.19377800	-0.43181300	3.64047900
H	-7.80120500	0.30962000	3.63029700
H	-8.38027400	0.11105100	0.30975600
H	-9.09139000	0.31718000	1.92246100
H	-5.33940400	-2.04622600	-0.26682300
H	-7.09314300	-2.01029400	-0.17712900
H	-5.29133300	0.10788400	-1.46996300
H	-7.04411200	0.23402100	-1.40595500
H	-7.31594800	-1.90418400	-2.63347900
H	-5.59188200	-2.19621900	-2.56584600
H	-4.40097900	0.32113300	-3.43816500
H	-0.21784100	-4.94289100	-8.59551800
H	0.61452000	-4.27592700	-7.18138600
H	-1.13500300	-4.48614500	-7.15583500
H	-1.64110100	-2.77505400	-5.80520500
H	-1.60347400	-1.04715300	-5.54089300
H	-2.60787300	-1.70153700	-6.84074100
H	-1.38509700	-2.72745000	-8.93416900
H	0.36221100	-2.58568900	-9.05647000
H	0.45535200	-1.76611600	-6.69493900
H	14.84577900	0.21933500	0.62342000
H	13.93524400	0.52800600	2.10479200
H	-1.52867100	7.11243700	1.46474300
H	0.08679800	0.33143400	8.94354700
H	-1.40318200	-0.16521800	-8.53854100
H	-6.27084800	-1.53715200	-4.82172700
H	-12.76952000	3.47554000	1.66394100
H	-13.54804200	1.98933900	2.20067200
H	2.41855900	-7.27356900	2.66080000
H	1.28331800	-6.38926800	1.62531800
H	-9.04315300	-6.57759400	0.39366100
H	-8.00832400	-6.68181500	1.83769200
H	-2.68840400	6.48666300	-2.86966700
H	-6.49217600	5.67487000	-0.65326000
H	0.36903400	-0.16600800	-8.62691600
H	-0.45326300	0.49964800	-7.20469200
H	-0.45352300	8.22620600	0.61190900
H	-0.01080200	6.50257200	0.75670800
H	-3.28106600	8.16761000	-2.98676500
H	-6.89407800	5.44233000	-2.34853200
H	-13.71866200	3.46151000	3.17645300
H	1.46373500	-0.12270700	7.90951100
H	0.49889100	-1.37207300	8.72074000
H	-8.84052300	-5.15180800	1.43650800
H	0.90281900	-6.65870100	3.32654100
H	-7.00458000	-0.05769600	-4.22229000
H	-3.47464700	0.60469000	-5.47464600
H	8.48670900	0.33143400	-1.74502300
H	-8.08172300	3.34963700	0.84327600

Int2 (9.9 kcal/mol)

C	0.54259500	1.07568400	7.86236700
C	0.57965500	-0.38584700	7.39974100
C	0.65907500	-1.32699600	8.61424100
C	1.73634800	-0.60571800	6.42068400
C	0.56973400	-2.81501600	8.26867300
C	4.47534900	1.08116100	5.32234400

O	5.05090900	0.93502200	6.40063800
N	4.92144200	0.65528100	4.13001800
C	6.23035300	0.05825400	4.05070100
C	6.39547900	-0.87388700	2.84278000
C	6.21933900	-0.26013800	1.43827800
C	6.20558100	-1.41200100	0.41857900
N	6.18046600	-1.10155600	-1.02195900
C	7.33713700	-0.77284500	-1.69636100
N	8.47336800	-0.47660100	-1.09112900
N	7.32497100	-0.84453300	-3.03110300
C	6.14670500	5.42856600	0.59975400
C	4.74280900	5.84407000	1.02021500
O	3.98174300	5.08976700	1.62238400
C	6.39394300	3.90551300	0.70581000
C	6.08205500	3.14984700	-0.56726600
C	7.09505900	2.88213600	-1.50007100
C	4.77842900	2.71813200	-0.84422800
C	6.80123500	2.23641900	-2.70031400
C	4.48650200	2.05526700	-2.03799900
C	5.49482400	1.82434500	-2.97632200
N	4.42321300	7.14550500	0.77413700
C	3.08412100	7.66145200	1.03095600
C	2.36172000	7.84449100	-0.29673500
O	2.71277900	8.73973400	-1.07042200
N	1.38814600	6.95447800	-0.56899700
C	0.71493200	7.03335500	-1.85101200
C	12.97653600	2.67792500	-3.11717000
C	11.80347100	1.84742700	-3.65854000
C	10.76818500	1.40847600	-2.59494200
O	10.03902700	0.39616600	-2.93961100
O	10.68971200	2.02735100	-1.52323700
C	8.46566100	-5.94063100	-0.08039200
C	5.93317100	-4.85239000	-0.33468000
S	7.07738300	-5.39764100	0.97442100
C	-1.58823800	-6.78847600	-1.52087600
C	-2.42159000	-5.66529400	-2.08739200
C	-3.61516500	-5.92646200	-2.76982000
C	-2.01367400	-4.33301300	-1.93995700
C	-4.37616200	-4.88486700	-3.30698300
C	-2.77324300	-3.29295000	-2.46898400
C	-3.95305400	-3.56348400	-3.16551700
C	-14.00572300	0.37154100	-1.07854000
C	-12.72501100	0.43826300	-0.26962100
C	-11.69298500	1.33123900	-0.58980400
C	-12.50972500	-0.42016200	0.81700800
C	-10.49872300	1.37167900	0.12987700
C	-11.32457200	-0.39679200	1.54681000
C	-10.29443400	0.50090800	1.21558800
O	-9.16639700	0.47797100	1.94611600
C	-0.45766600	-1.76079800	-8.05014300
C	0.37808300	-1.59156300	-6.77496200
C	0.39832900	-0.15773300	-6.21353300
C	1.01452700	-0.12769200	-4.80866600
C	1.13836400	0.81276600	-7.14186600
N	-2.44796600	4.95537000	0.80466800
N	-2.48103500	3.05398000	-0.48293400
N	-4.47971300	4.19178500	-0.14486600
N	-3.55061900	-6.56701800	1.03413400
N	-2.81301200	-4.53400800	1.47747000
N	-5.76782500	0.36433800	-0.16350000
N	-3.36785800	-0.79497000	-5.91842000
N	-3.59625900	0.19065900	-3.92854900
N	-0.99266200	0.58827000	2.81135200
N	1.55727500	-0.50645100	2.41598700

N	-0.34646200	2.64391400	1.92161000
N	1.94546700	3.07647800	1.63531000
C	-9.76768200	4.11262300	2.59601600
C	-8.42686600	3.54753100	3.10410900
C	-7.36010900	3.34556200	2.00193700
C	-8.72120300	0.20876000	-3.84733600
C	-8.36966600	1.10839600	-2.65666800
C	-7.13353700	1.98859400	-2.94569500
C	-1.61275000	7.22894300	1.34206800
C	-2.84824500	6.29965000	1.21664200
C	-3.15647600	4.06687900	0.08439700
C	2.54226400	-4.95490200	-4.25890700
C	3.19002400	-3.67941300	-4.80783200
C	4.27905100	-3.02260500	-3.98442000
C	-2.00149000	-8.09379700	2.34228100
C	-2.48400100	-6.74062500	1.90792500
C	-3.69716300	-5.23063200	0.79646500
C	-2.05181000	-5.45936800	2.17039800
C	-6.45792800	-1.88954400	-1.03340900
C	-6.48546600	-0.90314300	0.13431400
C	-5.38128400	0.14610400	-7.13290600
C	-4.48836700	0.02245000	-5.93378400
C	-2.86581200	-0.66306400	-4.69757900
C	-4.63790200	0.64112200	-4.71077300
C	-2.39172300	0.91130200	2.60588400
C	-0.66856000	-0.64650000	3.39730900
C	-1.61172200	-1.37753000	4.11611800
C	-1.31936000	-2.65613600	4.60040200
C	-2.38828800	-3.37569900	5.39214100
C	-0.07731000	-3.25640000	4.31312600
C	0.25552000	-4.57448300	4.98810700
C	0.84682800	-2.56184100	3.49377500
C	0.59312100	-1.22126300	3.16050200
C	0.00465400	1.40949600	2.31948200
C	1.30956500	0.90003000	2.30262500
C	2.35484200	1.77945800	1.95643400
C	0.63230000	3.48701600	1.53086000
C	-1.99592000	-0.09059900	-0.78645400
C	-1.88098400	-1.26360300	0.03292600
C	-0.64319200	-1.70760800	0.48005500
C	-0.77753400	0.51995500	-1.16210500
C	0.44947100	0.12335700	-0.62681700
C	0.54460300	-0.98709800	0.21901300
C	2.01378300	-1.85540500	-2.07678400
C	1.77783400	-1.17847800	1.08233800
C	2.16721500	-2.64555300	1.35418800
C	2.11049100	-3.14479600	2.84027600
C	2.15110000	-4.68131800	2.71770700
C	3.35391900	-2.68480600	3.62210300
O	-7.39104200	2.22646700	1.37114600
O	-6.54722800	4.27494400	1.81071100
O	-7.13557500	2.63973200	-4.00118700
O	-6.17388900	1.96208800	-2.08433100
O	4.37840400	-3.48207900	-2.72316700
O	4.98559100	-2.12170500	-4.40566100
O	3.58355800	1.50394100	1.86546500
O	0.37714700	4.64155600	1.07566000
O	-3.16426200	0.38978800	-1.14617200
O	-3.05043000	-1.87355400	0.39904200
O	2.93517700	-1.15143400	-1.89386900
O	1.17947500	-2.62421500	-2.35746400
H	-0.86810400	6.78648400	2.00800200
H	-1.13835800	7.38637200	0.37130600
H	-3.37415700	6.26019100	2.17983100

H	-3.56159300	6.69248900	0.48357500
H	-1.46054000	4.71896700	0.98456800
H	-1.51474100	2.94038600	-0.20557500
H	-2.94818400	2.17732400	-0.77315000
H	-5.14073300	4.41080100	0.62168100
H	-4.88488700	3.53110500	-0.81034300
H	2.06870600	-4.76762100	-3.29457000
H	1.77170000	-5.29798100	-4.95411600
H	3.61752400	-3.83653800	-5.80335700
H	2.42624600	-2.90407400	-4.94362500
H	-1.16222800	-7.97662000	3.03154200
H	-1.65446900	-8.69042900	1.48982700
H	-1.24758100	-5.13597300	2.81155600
H	-4.42292800	-4.82935400	0.10468600
H	-5.42663800	-2.16216600	-1.27166900
H	-6.91984300	-1.46151300	-1.92744300
H	-6.00845900	-1.35519900	1.00988100
H	-7.50765800	-0.64019300	0.41580200
H	-6.09127600	0.93536200	-1.07159000
H	-5.96794000	1.05420300	0.57739900
H	-5.79686100	-0.82715200	-7.42256200
H	-6.21145900	0.82275200	-6.91199300
H	-5.34532900	1.38118500	-4.36887900
H	-1.98805800	-1.16987500	-4.32488400
H	-2.44830200	1.76693900	1.93984200
H	-2.90537700	0.05841000	2.15399100
H	-2.59731200	-0.96450000	4.28923900
H	-2.11484600	-3.51027600	6.44635600
H	-3.32069200	-2.80619900	5.36971500
H	-2.60051000	-4.36894000	4.98161500
H	1.28664300	-4.58295400	5.34755400
H	-0.38136800	-4.72308700	5.86037600
H	0.13038900	-5.45719200	4.35047300
H	-4.74260200	0.21835500	-0.30774400
H	-2.85176300	-2.68993400	0.90969500
H	-0.62220100	-2.57942100	1.12557600
H	-0.82939100	1.37067500	-1.83551400
H	1.33809200	0.71406400	-0.83251400
H	-4.06455600	-7.30058200	0.57205900
H	-3.45144200	0.38997100	-2.92306500
H	2.67735500	3.76903600	1.48861000
H	-2.78318300	-8.66464600	2.85759800
H	3.27646200	-5.75661600	-4.13433500
H	-1.92530300	8.19708300	1.74408600
H	-4.83992500	0.53567500	-8.00440200
H	-7.00740200	-2.79695800	-0.76302600
H	-2.88759100	1.17717200	3.54765800
H	2.61662600	-0.64153700	0.63604600
H	1.51611900	-3.29452400	0.76092500
H	3.18839200	-2.82650700	0.99427100
H	2.37012000	-5.18240200	3.66033600
H	1.21559100	-5.08028900	2.31349600
H	2.95239800	-4.95237700	2.02027200
H	3.30151500	-3.02539000	4.66156500
H	4.25667200	-3.12045500	3.17753600
H	3.44079800	-1.59808200	3.61969300
H	-8.00144400	4.23434300	3.84253800
H	-8.60975000	2.58392100	3.59130700
H	-10.24611600	3.42610300	1.89259400
H	-9.61269200	5.06959800	2.08638400
H	-8.23452000	0.51958900	-1.74480800
H	-9.20982100	1.78558200	-2.46359200
H	-9.64078800	-0.35925100	-3.66659300
H	-7.91228400	-0.50182000	-4.05479800

H	5.00472400	-2.90105200	-2.24591200
H	-8.84666600	0.82726500	-4.73953100
H	-10.45941200	4.27663100	3.42969300
H	-9.70657800	2.06065400	-0.14203100
H	-11.17184300	-1.06521500	2.38862700
H	-13.28987700	-1.12539800	1.09912100
H	-11.81787400	2.01201300	-1.42918900
H	-14.26892700	1.35136200	-1.49085900
H	-3.94777500	-6.95640100	-2.88793500
H	-5.29700300	-5.10635900	-3.83944500
H	-4.52591300	-2.74753800	-3.59260800
H	-2.45478600	-2.26986200	-2.32001900
H	-1.10712200	-4.09792400	-1.39010700
H	-0.02880500	-2.25989600	-6.00440900
H	1.41527000	-1.91677600	-6.95376700
H	-0.64507400	0.17070500	-6.12913200
H	2.05707200	-0.47559900	-4.82657900
H	1.01751500	0.88649700	-4.39580500
H	0.44935400	-0.75756600	-4.11550000
H	2.18728500	0.51135000	-7.26311300
H	0.68398400	0.85467000	-8.13635900
H	1.13038500	1.82827200	-6.73194000
H	1.18642700	6.16625500	0.05311200
H	4.98397900	7.67038000	0.11725200
H	7.60736800	2.02448100	-3.39515800
H	5.26657800	1.30676100	-3.90381100
H	3.47332100	1.71495800	-2.23191700
H	4.00487400	2.86424700	-0.10312400
H	7.44602200	3.74669600	0.96231400
H	5.78240300	3.51647800	1.52502300
H	11.25386000	2.42759000	-4.41345800
H	12.15669700	0.94887400	-4.17628500
H	5.66283800	-5.67565800	-1.00001200
H	5.02806600	-4.48260300	0.15074100
H	6.38116600	-4.04337200	-0.91618800
H	5.35550500	-0.61162600	-1.36112900
H	6.50996100	-1.18636300	-3.53252600
H	8.11740800	-0.43164600	-3.51491400
H	8.41635500	-0.13068900	-0.14515200
H	9.25724600	-0.08061500	-1.76176500
H	5.31408300	-2.01590300	0.61121500
H	7.06453100	-2.07267400	0.57820200
H	5.28288100	0.29866600	1.36260200
H	7.02566200	0.45518900	1.23004200
H	7.39434200	-1.32664600	2.90736200
H	5.68305000	-1.69492100	2.95048400
H	4.39575400	0.91083800	3.27983700
H	0.55373400	-3.43545200	9.17186200
H	-0.34091300	-3.02560500	7.69879700
H	1.41659200	-3.13997800	7.65794400
H	1.75595900	-1.62749100	6.03746000
H	1.63507900	0.04758500	5.54980200
H	2.70056500	-0.38944600	6.89431500
H	1.59704500	-1.12997700	9.15325100
H	-0.15241700	-1.07254300	9.31027700
H	-0.35744400	-0.60309200	6.86677200
H	-14.84598700	0.02886900	-0.46501400
H	-13.91845100	-0.32124600	-1.92629400
H	1.39945800	6.81124800	-2.67728700
H	-0.08553700	-1.13476400	-8.86807200
H	1.47304200	1.34102700	8.37883000
H	6.39828300	-0.50235700	4.97403500
H	12.59634200	3.52855100	-2.54629500
H	13.59433600	2.08332000	-2.43610900

H	-2.17069800	-7.71027400	-1.41894300
H	-1.18717300	-6.52641900	-0.53777700
H	9.25506500	-6.30155200	0.58208200
H	8.15925900	-6.75357400	-0.74451400
H	2.58621300	6.94854800	1.68918500
H	6.35518600	5.78857000	-0.41442000
H	-0.29119700	1.26069100	8.54998400
H	0.43328000	1.75390800	7.00990900
H	0.31628300	8.03895200	-2.01045200
H	-0.10284800	6.31080900	-1.85956000
H	3.15135700	8.63606800	1.52319500
H	6.83692000	5.96339600	1.26538700
H	13.61525900	3.04434800	-3.92883300
H	-1.49820600	-1.48871600	-7.84220200
H	-0.43884300	-2.80058500	-8.39591500
H	8.85482000	-5.10823200	-0.67304100
H	-0.73364500	-7.01572600	-2.17118600
H	7.00793100	0.83917600	4.01827100
H	3.49229100	1.58119500	5.24327900
H	-8.49914700	1.21641300	1.68798900
H	8.12801200	3.14473400	-1.28420600

TS3 (15.3 kcal/mol)

C	1.03866800	2.19642900	-7.60618600
C	0.88216900	3.28023000	-6.53286300
C	1.04264700	4.67593600	-7.16002700
C	1.87396000	3.04751800	-5.39005900
C	0.83927000	5.83542100	-6.18244300
C	4.87158800	1.43317500	-5.02895300
O	5.35107500	2.20694800	-5.85590200
N	5.37745200	1.19988200	-3.80598600
C	6.51081700	1.99061200	-3.37205700
C	6.83169900	1.73546800	-1.90118700
C	7.48156500	0.36866100	-1.67496900
C	7.73537100	0.02467700	-0.21289500
N	6.47023000	-0.18434500	0.49964100
C	6.43707300	-0.63131500	1.76924500
N	7.54497900	-1.01549100	2.38143700
N	5.24783900	-0.60512000	2.43105300
C	6.65599200	-4.27487900	-2.15951700
C	5.32744300	-4.58730000	-2.82810200
O	4.56795200	-3.69012400	-3.19633800
C	6.52185000	-3.10878300	-1.16204500
C	5.81027700	-3.47012700	0.12259700
C	6.54087300	-3.87503700	1.24674600
C	4.41457000	-3.37486500	0.22463700
C	5.88904100	-4.18545600	2.44232200
C	3.76220600	-3.68477100	1.41667800
C	4.49809100	-4.09330000	2.53193800
N	5.06073100	-5.90062100	-3.05579400
C	3.76218800	-6.32979200	-3.55810700
C	3.01175900	-7.03702900	-2.43870500
O	3.37555900	-8.15468400	-2.06396100
N	1.99038600	-6.34793000	-1.89250000
C	1.25752700	-6.93221600	-0.78767600
C	13.15721500	-2.78438500	2.69220100
C	11.97334600	-2.70650200	1.71057600
C	10.63551400	-2.17149900	2.28851600
O	10.54503300	-1.90868100	3.49530000
O	9.69212700	-2.05414400	1.40806400
C	8.20406600	6.12115500	2.80257100
C	6.60127100	4.24555700	1.47173200
S	7.59617700	5.73773900	1.12145000
C	-1.92867300	5.89470500	3.88176100
C	-2.57931600	4.54386100	4.04550100
C	-3.89982400	4.42723700	4.49163400
C	-1.85910100	3.37532400	3.75786800
C	-4.48838300	3.17212800	4.66701600
C	-2.44573700	2.12370100	3.93628200
C	-3.76007600	2.01232600	4.40154200
C	-13.82977200	-1.16779300	0.12098400
C	-12.52399900	-0.69919800	-0.48904500
C	-11.46029400	-1.58111000	-0.72236400
C	-12.31711200	0.64868900	-0.81115600
C	-10.24266200	-1.14916700	-1.24799100
C	-11.10983600	1.09913800	-1.33778600
C	-10.05015200	0.20585000	-1.56517900
O	-8.90696200	0.69441500	-2.08795400
C	-0.70217500	-1.14224100	8.14448200
C	-1.05725900	-1.59443700	6.72457800
C	-0.70195400	-3.06036800	6.41305800
C	-1.25577000	-3.47831700	5.04434900
C	0.80813800	-3.32879000	6.49017900
N	-1.98399400	-4.17240800	-2.58779500
N	-3.49303300	-3.12769900	-1.12364700
N	-4.30692500	-4.39984400	-2.83795200
N	-3.76155500	6.52215000	1.27873900
N	-2.65540600	4.79660300	0.45524700
N	-5.41981300	-0.23828300	-0.35085400
N	-4.07770100	-1.37734800	4.73634900
N	-4.47850700	-2.40800200	2.81111300
N	-0.68800900	0.66269700	-2.64842200
N	1.80065200	1.55552400	-1.67940200
N	0.05455100	-1.56226400	-2.65722900
N	2.36344600	-1.98843500	-2.39722500
C	-9.23027500	-3.06733700	-4.42867900
C	-8.37906000	-1.79886100	-4.33952500
C	-7.19262900	-1.95114700	-3.36875600
C	-8.66748800	-1.79611100	3.04932900
C	-8.38037900	-2.02893300	1.55010500
C	-7.18989600	-2.98137600	1.29260800
C	-0.93896100	-6.03587600	-3.95361800
C	-1.58872000	-4.63862300	-3.92529900
C	-3.24208000	-3.88560800	-2.19763200
C	2.21550100	3.37113500	5.97618400
C	2.46979700	2.15542900	5.08518200
C	2.06474400	2.35607200	3.62111200
C	-2.28920200	8.52173200	0.75623400
C	-2.61831900	7.05718700	0.69788400
C	-3.72993000	5.16636800	1.11782500
C	-1.95491300	5.96144200	0.19058600
C	-6.44815700	1.30256500	1.34204800
C	-6.08461600	1.07706400	-0.12101900
C	-5.45606800	-2.80229600	6.30935100
C	-4.92089400	-2.45636100	4.95919200
C	-3.84539300	-1.37783400	3.42982600
C	-5.17948200	-3.10012600	3.77363000
C	-2.08284900	0.25374700	-2.83638100
C	-0.39373900	2.04266600	-2.60810800
C	-1.32419800	3.00089900	-3.00381200
C	-1.03834900	4.36736900	-2.92750700
C	-2.10468800	5.33679500	-3.38783600
C	0.19682700	4.81117400	-2.40987800
C	0.52808500	6.28784200	-2.51731500
C	1.11152200	3.85709300	-1.90465000
C	0.83669400	2.48963400	-2.08985800
C	0.33832600	-0.26152300	-2.48716100

C	1.61448200	0.22822700	-2.14598600
C	2.71018200	-0.66331400	-2.13530400
C	1.06429600	-2.45554100	-2.52757300
C	-1.59519200	-0.24684000	0.45933800
C	-1.49663200	1.20250100	0.53226300
C	-0.27633000	1.82116000	0.61825100
C	-0.36496200	-0.97164500	0.58448000
C	0.85287900	-0.33864900	0.58577700
C	0.98213800	1.09529400	0.63414200
C	1.15497000	-0.90445900	3.56579800
C	2.13224500	1.67102200	-0.23593600
C	2.48982500	3.13529400	0.07015100
C	2.41818800	4.12993400	-1.13155200
C	2.53168500	5.51834300	-0.47255100
C	3.62635600	3.93193000	-2.07381000
O	-7.01408800	-1.01895400	-2.51293000
O	-6.48263400	-2.96943800	-3.51788100
O	-7.26705800	-4.12808000	1.75011100
O	-6.17964800	-2.50940800	0.62534700
O	2.38489900	1.34866800	2.84806000
O	1.47342100	3.37353900	3.25586000
O	3.91914700	-0.38372300	-1.86511100
O	0.86644700	-3.70267300	-2.53123700
O	-2.71616800	-0.83965800	0.28061600
O	-2.68084800	1.87748600	0.42895200
O	0.19529600	-0.29069500	3.81954400
O	2.08961500	-1.58253200	3.36537700
H	-0.02071400	-6.03878400	-3.36748200
H	-1.61521800	-6.79978100	-3.55546300
H	-0.87159300	-3.90603200	-4.30068900
H	-2.46365900	-4.60649700	-4.58160900
H	-1.20065800	-3.74621500	-2.09364700
H	-2.85580000	-2.44104000	-0.70049200
H	-4.44019400	-3.12855100	-0.74365600
H	-4.13667700	-5.02237000	-3.61006200
H	-5.17057300	-3.81675100	-2.90597100
H	1.15929300	3.65007800	5.94153100
H	2.49122400	3.16137700	7.01511900
H	3.52230400	1.84774400	5.10500200
H	1.90061200	1.29110600	5.44991900
H	-1.36741200	8.70925000	0.20115700
H	-2.13338900	8.85731500	1.78866700
H	-1.02059800	5.92883500	-0.34531100
H	-4.47999400	4.50593400	1.52631500
H	-5.55347900	1.32966200	1.97271200
H	-7.11028300	0.51847600	1.71470500
H	-5.39849700	1.85309800	-0.47062900
H	-6.98186700	1.09759900	-0.74179200
H	-5.87032700	-1.06398300	0.18840500
H	-5.59047500	-0.53658100	-1.32702400
H	-6.00198000	-1.95889000	6.74934500
H	-6.14090900	-3.65262400	6.24237400
H	-5.82082300	-3.92307100	3.50183000
H	-3.23959900	-0.67041300	2.88357300
H	-2.13683900	-0.81886800	-2.67357600
H	-2.71220100	0.76756100	-2.10596000
H	-2.29487900	2.69399200	-3.37140600
H	-1.82003600	5.87963000	-4.29803200
H	-3.03121600	4.79990600	-3.60676000
H	-2.33309900	6.08395300	-2.62024200
H	1.54969700	6.43432800	-2.87301000
H	-0.12962400	6.77353000	-3.23835400
H	0.43647200	6.84024900	-1.57554100
H	-4.40183700	-0.22738600	-0.12068000

H	-2.51105600	2.84581900	0.38668000
H	-0.25098300	2.90576800	0.64666900
H	-0.43155700	-2.05568100	0.57420500
H	1.76368900	-0.93341100	0.57984600
H	-4.45123900	7.03264700	1.80726100
H	-4.67740800	-2.51548500	1.81595700
H	3.12570100	-2.65018300	-2.53289000
H	-3.08017200	9.13947900	0.31451200
H	2.79006200	4.23596000	5.63104000
H	-0.68927300	-6.29789800	-4.98735100
H	-4.65517400	-3.06499600	7.01256100
H	-6.96615400	2.26152100	1.44470600
H	-2.42735800	0.47910800	-3.85224500
H	3.00709400	1.03432800	-0.08847600
H	1.85236300	3.50695500	0.87585500
H	3.50956600	3.17846500	0.46864400
H	2.74255800	6.31815800	-1.18313600
H	1.62859900	5.77052000	0.09104900
H	3.36279100	5.49485600	0.24021400
H	3.59113100	4.64333500	-2.90573900
H	4.56163600	4.10377400	-1.52611700
H	3.64376700	2.92161300	-2.48235300
H	-7.94478500	-1.58304200	-5.32496100
H	-8.98701900	-0.93473300	-4.06191900
H	-9.74540500	-3.25738200	-3.48152600
H	-8.59057900	-3.92665100	-4.64418100
H	-8.23870500	-1.07918700	1.02712000
H	-9.26175800	-2.49816800	1.10034100
H	-9.56415700	-1.18207800	3.18469000
H	-7.82520900	-1.29871300	3.54243600
H	1.62752500	1.29508100	1.81236600
H	-8.81508300	-2.76049600	3.54280000
H	-9.99261700	-2.98402500	-5.21138400
H	-9.42591300	-1.84682700	-1.39497100
H	-10.96329100	2.14542000	-1.58750000
H	-13.12089800	1.36401600	-0.64612400
H	-11.57774200	-2.63516500	-0.48053200
H	-14.06198700	-2.19641100	-0.17435400
H	-4.46907300	5.32901100	4.71410200
H	-5.51279300	3.09969700	5.02372500
H	-4.18994300	1.03095900	4.57572200
H	-1.86893400	1.23123300	3.71777800
H	-0.83370100	3.44581900	3.40490500
H	-2.12667800	-1.44458900	6.53866600
H	-0.54915100	-0.94569900	6.00262800
H	-1.18837400	-3.68806400	7.17728300
H	-0.86261500	-2.83498900	4.24985400
H	-0.97881800	-4.51183600	4.80560200
H	-2.34401100	-3.39486500	5.01060700
H	1.35129300	-2.73616900	5.74456300
H	1.22259300	-3.08566700	7.47387900
H	1.02898500	-4.38326700	6.28878600
H	1.78860100	-5.37726000	-2.15670900
H	5.60949200	-6.60153800	-2.57775700
H	6.47405000	-4.48192500	3.30864600
H	3.98909400	-4.32181800	3.46377200
H	2.68431700	-3.58000600	1.48510000
H	3.84070800	-3.02016700	-0.62109300
H	7.52594300	-2.74833000	-0.92406800
H	5.97402400	-2.30908600	-1.66770500
H	12.22755000	-2.07714500	0.84819900
H	11.76370300	-3.69809100	1.28751700
H	5.77590400	4.46972100	2.15200100
H	6.18745700	3.90917900	0.51886000

H	7.22030300	3.44596100	1.88701500
H	5.61525400	-0.25081400	-0.04698800
H	4.44701000	-0.12812400	2.03797400
H	5.02539600	-1.38585400	3.03119800
H	8.48784000	-1.42986700	1.92658000
H	7.46790900	-1.18717700	3.37408600
H	8.28152400	0.83634600	0.28496800
H	8.36779900	-0.86385100	-0.13369600
H	6.86317300	-0.41156200	-2.12987200
H	8.44581000	0.34089300	-2.19621900
H	7.50669500	2.52176300	-1.54065200
H	5.90734900	1.82098500	-1.31813200
H	4.84081000	0.64200900	-3.12895500
H	0.86728600	6.80217700	-6.69762800
H	-0.12660300	5.75053800	-5.67417100
H	1.61145000	5.84849400	-5.40905900
H	1.71829000	3.74732800	-4.56821100
H	1.75619200	2.04338800	-4.97095200
H	2.90546600	3.14774100	-5.74489800
H	2.04407000	4.74646300	-7.60826000
H	0.32824400	4.77388300	-7.98964000
H	-0.13449900	3.21424300	-6.11718900
H	-14.66516900	-0.53183700	-0.19069900
H	-13.79667500	-1.14768900	1.21836600
H	1.80143300	-6.82915900	0.15924900
H	0.37784900	-1.16112700	8.32694900
H	2.05509900	2.20939200	-8.01779300
H	6.28627200	3.05196300	-3.53414200
H	13.39983400	-1.79250300	3.08501600
H	12.90289200	-3.41292500	3.55024400
H	-2.65398300	6.70735000	3.99391100
H	-1.45367700	5.98862500	2.90187400
H	8.82504800	7.01589400	2.72175900
H	7.37356600	6.32443700	3.48410400
H	3.24700000	-5.43805900	-3.91670400
H	7.07407400	-5.16216200	-1.67125000
H	0.33552500	2.34200900	-8.43517900
H	0.86497000	1.19980200	-7.18699100
H	1.10168000	-7.99631900	-0.97452000
H	0.29440800	-6.42596800	-0.69792600
H	3.89492900	-7.03765300	-4.38180300
H	7.34943900	-3.98400000	-2.95851600
H	14.04807800	-3.19525100	2.20329900
H	-1.17476400	-1.78657100	8.89687500
H	-1.04568200	-0.11793900	8.32314900
H	8.81029600	5.30159400	3.19788900
H	-1.14238200	6.04467200	4.63151400
H	7.38344900	1.76446400	-3.99937800
H	3.95844300	0.84226900	-5.22835400
H	-8.20962900	-0.02600700	-2.25946600
H	7.62712500	-3.88230000	1.20456800

Int3 (7.1 kcal/mol)

C	-0.42132200	0.86855000	-7.92231800
C	-0.36549800	-0.53415900	-7.30288300
C	-0.59092900	-1.59990800	-8.38954900
C	-1.37748600	-0.66072700	-6.16008400
C	-0.48473800	-3.04339100	-7.89377500
C	-4.39845100	0.84433600	-5.45292200
O	-4.90193500	0.54506200	-6.53466100
N	-4.91305000	0.54804200	-4.24768700
C	-6.15803200	-0.18827900	-4.19575100
C	-6.48966800	-0.68759000	-2.78755700
C	-6.70960900	0.45051800	-1.78358800
C	-7.52894000	0.08352700	-0.55449900
N	-6.81361100	-0.80641100	0.36638700
C	-7.31770700	-1.08069600	1.59231400
N	-8.51756100	-0.62118200	1.92059400
N	-6.61451700	-1.87930800	2.41202700
C	-6.23113700	5.23701300	-0.83282600
C	-4.80142100	5.66216200	-1.13707400
O	-3.95631000	4.88095200	-1.57070400
C	-6.38129100	3.71690600	-0.64929400
C	-6.14175600	3.23724500	0.76465500
C	-7.22383700	2.85031400	1.56571500
C	-4.84713100	3.15538100	1.29478000
C	-7.02389600	2.37404400	2.86238900
C	-4.64620600	2.70745200	2.59988700
C	-5.73179400	2.30967400	3.38569700
N	-4.53799400	6.99200700	-1.00582200
C	-3.20095100	7.51734700	-1.24635400
C	-2.48344900	7.75622900	0.07564800
O	-2.77644700	8.72958900	0.77395700
N	-1.56379900	6.83277000	0.42160200
C	-0.87343400	6.97845700	1.68685800
C	-13.07700500	2.42508900	2.80777400
C	-11.84190400	2.62028100	1.92583300
C	-10.76267100	1.52386500	2.08564200
O	-10.65664000	0.93614200	3.18383300
O	-10.02912000	1.34017100	1.05113000
C	-8.36085200	-6.15931600	-0.00655500
C	-6.21396700	-4.36545400	-0.01267400
S	-6.92118100	-5.69406400	1.01857800
C	1.67928100	-6.80423700	1.62606400
C	1.94843800	-5.48848700	2.31555200
C	3.21153800	-5.17837600	2.83340900
C	0.91780700	-4.55209500	2.46066000
C	3.43630800	-3.96892500	3.49618100
C	1.13871200	-3.34910900	3.12770000
C	2.39612400	-3.05259500	3.65829600
C	13.97411300	0.56986800	1.28526500
C	12.74160900	0.40654100	0.41830400
C	11.66292100	1.29749200	0.49227400
C	12.61945900	-0.67127500	-0.46906900
C	10.50790400	1.12700600	-0.27199700
C	11.47695900	-0.85745900	-1.24181000
C	10.39999100	0.03985600	-1.15435600
O	9.31687500	-0.19089100	-1.92622800
C	0.34218200	-1.69217500	8.05026000
C	0.55688000	-1.36089800	6.56846400
C	-0.27243200	-0.16833800	6.05971100
C	0.07548400	0.15423600	4.60165000
C	-1.78005300	-0.40177800	6.21144300
N	2.40464100	4.88588200	-0.83235500
N	3.81320800	3.33293200	0.21339500
N	4.74220700	5.05591000	-0.96540400
N	3.71977800	-6.61097800	-0.92072400
N	2.82551200	-4.61149600	-1.19176900
N	5.76263000	0.25481600	-0.04906800
N	3.94125200	-0.31139600	5.09934200
N	4.69078700	1.24944300	3.70673200
N	1.04550800	0.38572500	-2.55050400
N	-1.45881900	-0.73778500	-2.03800100
N	0.35549300	2.47480400	-1.74360700
N	-1.95025300	2.84233100	-1.40566400
C	9.73721000	4.17446400	-2.52507000
C	8.88059400	3.01974200	-3.04882700

C	7.63359200	2.78286400	-2.18013300
C	8.64422000	0.35692500	3.96224900
C	8.52618400	1.07799100	2.60296400
C	7.42250100	2.15922400	2.58234000
C	1.50754900	7.16404000	-1.46713300
C	2.06642900	5.80434100	-1.92723700
C	3.63330900	4.42092200	-0.54877600
C	-2.53330200	-4.99921700	4.25765600
C	-3.65630300	-4.22602800	3.55618800
C	-3.17852200	-3.32674600	2.39827500
C	2.18400300	-8.16317900	-2.20800700
C	2.61307900	-6.80505600	-1.73684300
C	3.79490200	-5.28389700	-0.60992100
C	2.08149800	-5.54419900	-1.89244400
C	6.46925500	-1.82575300	1.14312800
C	6.40878400	-1.07926100	-0.18330500
C	5.24725800	0.28788300	7.18850000
C	4.84636800	0.50189500	5.76512900
C	3.88745500	0.16612700	3.86227700
C	5.31845000	1.47013200	4.91249200
C	2.42035300	0.84182400	-2.61266300
C	0.72931300	-0.88164300	-3.07193400
C	1.65107000	-1.62718400	-3.80396600
C	1.34038200	-2.90760400	-4.26982600
C	2.40213300	-3.64671500	-5.05411200
C	0.08716800	-3.48989400	-3.98054200
C	-0.26108000	-4.80475100	-4.65367600
C	-0.82134900	-2.78909300	-3.15251400
C	-0.51609300	-1.46672800	-2.77938700
C	0.03642700	1.21235200	-2.07020300
C	-1.24204900	0.66175400	-1.95011800
C	-2.33518700	1.53788900	-1.72468700
C	-0.64108200	3.28494100	-1.31425000
C	1.88483700	0.03736900	0.52630100
C	1.78045500	-1.33472400	0.01954700
C	0.57536000	-1.95315700	-0.06247800
C	0.66553800	0.66528800	0.97906900
C	-0.54279700	0.07120900	0.83290400
C	-0.70487000	-1.30238800	0.31563700
C	-3.78606600	-0.43775800	3.57824300
C	-1.84481500	-1.39720700	-0.78665700
C	-2.24689900	-2.85195200	-1.08353700
C	-2.15276600	-3.30416800	-2.57328100
C	-2.31073100	-4.83583600	-2.50752000
C	-3.32369800	-2.72396900	-3.39579400
O	7.43102000	1.58771400	-1.77016400
O	6.90666500	3.77414400	-1.96194300
O	7.51069500	3.08438900	3.39679600
O	6.45433100	2.01069300	1.72528300
O	-4.06276000	-2.60909000	1.81020600
O	-1.96148100	-3.34935900	2.11371100
O	-3.55439800	1.23880900	-1.75723300
O	-0.41214600	4.43856400	-0.85074800
O	2.99217200	0.64517300	0.56939300
O	2.96022100	-1.88199100	-0.38624100
O	-2.98500500	0.02603700	2.86933500
O	-4.57240100	-0.85114000	4.34143100
H	0.58297800	7.02146000	-0.90679200
H	2.22445800	7.69812400	-0.83477600
H	1.31016300	5.29145000	-2.52566900
H	2.94780400	5.94062900	-2.56136800
H	1.57888400	4.37546700	-0.50917400
H	3.13442500	2.57791300	0.32502300
H	4.74437100	3.14489200	0.58318300
H	4.63628200	5.91723900	-1.47431400
H	5.58304000	4.48488900	-1.18890700
H	-1.77044400	-4.31533600	4.63858500
H	-2.92537700	-5.58942500	5.09398700
H	-4.41452200	-4.91179800	3.15447500
H	-4.19058900	-3.58436300	4.26816600
H	1.31393700	-8.06323000	-2.86066400
H	1.90034200	-8.80849700	-1.36804300
H	1.21510500	-5.24095700	-2.45714700
H	4.54135500	-4.87055300	0.05152500
H	5.46203800	-2.03389900	1.52009000
H	7.00753600	-1.25336100	1.90143800
H	5.83570100	-1.64913300	-0.91964100
H	7.40969600	-0.91290600	-0.58602300
H	6.14840700	0.83755800	0.77959900
H	6.04314100	0.84735400	-0.85688500
H	5.67244100	-0.71228100	7.33657100
H	5.99875900	1.02431500	7.48757000
H	6.05156300	2.25357000	5.01583700
H	3.29798200	-0.23317900	3.05051500
H	2.49727500	1.75896900	-2.03684800
H	3.07893800	0.08414500	-2.18181500
H	2.63292900	-1.22215400	-4.01134700
H	2.12679900	-3.79673700	-6.10534400
H	3.33856600	-3.08324500	-5.04200700
H	2.60904700	-4.63446800	-4.62752300
H	-1.27465800	-4.78270100	-5.05854600
H	0.40895300	-4.99259700	-5.49255200
H	-0.20037300	-5.67737400	-3.99371600
H	4.73195400	0.18908800	0.05600800
H	2.79825100	-2.78742100	-0.74661300
H	0.53978200	-2.97921400	-0.41163700
H	0.75943000	1.66649600	1.38668600
H	-1.44310700	0.58751400	1.14545100
H	4.31448600	-7.33626300	-0.55196500
H	5.00458100	1.68623900	2.83554400
H	-2.69663300	3.52572900	-1.29617500
H	2.97336800	-8.67101500	-2.77476400
H	-2.03105300	-5.67511300	3.56078700
H	1.28775700	7.78314100	-2.34335600
H	4.39347900	0.37480300	7.87233500
H	6.98875400	-2.77950100	1.00507700
H	2.72816200	1.04961200	-3.64568200
H	-2.69953700	-0.85160100	-0.38446800
H	-1.63891400	-3.52534200	-0.47557800
H	-3.27104200	-3.00752900	-0.73594200
H	-2.51905700	-5.29138000	-3.47616600
H	-1.42699300	-5.31441900	-2.07399100
H	-3.15816200	-5.06509800	-1.85283800
H	-3.25999000	-3.04622500	-4.44103900
H	-4.27766200	-3.08167400	-2.99030400
H	-3.32254400	-1.63397300	-3.37072900
H	8.51772800	3.26377900	-4.05595900
H	9.46528700	2.10028500	-3.12920400
H	10.17953000	3.92223000	-1.55598200
H	9.11714800	5.06473700	-2.39352400
H	8.38339100	0.35881500	1.79162600
H	9.47379300	1.58740200	2.39989300
H	9.48056900	-0.35017800	3.95713700
H	7.72632600	-0.18824500	4.20588700
H	-1.17361700	-1.91163900	1.12319600
H	8.80621000	1.09566100	4.75164200
H	10.55621400	4.40974600	-3.21381100
H	9.67489900	1.81470800	-0.17647900

H	11.39494700	-1.69419800	-1.92859700
H	13.43854100	-1.38283000	-0.55676900
H	11.71677400	2.14500600	1.17186900
H	14.15532000	1.62241500	1.52602400
H	4.02263400	-5.89789900	2.72884800
H	4.41790300	-3.73953500	3.90169400
H	2.57486000	-2.13738900	4.21200700
H	0.30979800	-2.65814200	3.22569200
H	-0.07575200	-4.74226400	2.06721700
H	1.61718500	-1.14574900	6.38988500
H	0.31929800	-2.24374500	5.95868900
H	-0.00147900	0.70529500	6.67341400
H	-0.21883100	-0.66440600	3.93690900
H	-0.45232600	1.04881300	4.25429500
H	1.14913900	0.31919600	4.47913800
H	-2.08743700	-1.29588700	5.65589500
H	-2.07923800	-0.54407100	7.25462700
H	-2.34860800	0.44970000	5.82226500
H	-1.39688400	5.98442700	-0.12953000
H	-5.16994200	7.56719300	-0.46776500
H	-7.88228100	2.04044700	3.43813500
H	-5.56803200	1.93927600	4.39375100
H	-3.63681900	2.64473200	2.99354700
H	-3.99581300	3.42377200	0.67906400
H	-7.39377800	3.43205500	-0.94900000
H	-5.68124700	3.22726700	-1.33247100
H	-12.10879300	2.68393500	0.86587200
H	-11.35725500	3.57515400	2.17581200
H	-6.94164900	-3.56568700	-0.16953600
H	-5.36506300	-3.95145100	0.53806900
H	-5.87429100	-4.75784300	-0.97576200
H	-5.81794900	-0.94096200	0.24842400
H	-5.65000600	-2.21294400	2.19088500
H	-6.87381700	-1.88426800	3.38476700
H	-9.05470700	0.17956000	1.44397500
H	-8.88765600	-0.79080200	2.84545900
H	-8.46513900	-0.40636100	-0.84939900
H	-7.80968000	0.99617700	-0.02847000
H	-5.75057300	0.87714600	-1.47509000
H	-7.26009800	1.25588600	-2.28522500
H	-7.40441600	-1.29067700	-2.86025900
H	-5.69790400	-1.35928000	-2.43914200
H	-4.40368300	0.79171300	-3.39039300
H	-0.57066400	-3.75700300	-8.72084200
H	0.47647000	-3.21498600	-7.39857700
H	-1.26852000	-3.27806500	-7.16903100
H	-1.28198500	-1.61277000	-5.63571700
H	-1.22659800	0.12408100	-5.41216200
H	-2.40063100	-0.57391600	-6.54159700
H	-1.58194200	-1.43889700	-8.83718900
H	0.13883600	-1.44125500	-9.19600900
H	0.63974400	-0.68886200	-6.88283200
H	14.86742800	0.18045100	0.78515900
H	13.87510600	0.03342900	2.23819700
H	-1.55123000	6.83176700	2.53548500
H	-0.68027700	-2.02724200	8.25374100
H	-1.42257300	1.07170800	-8.32103100
H	-6.09106300	-1.03095300	-4.89326400
H	-13.63836300	1.53405900	2.50486000
H	-12.76479600	2.27242500	3.84401700
H	2.57678700	-7.42969400	1.58788100
H	1.32921400	-6.64883800	0.60103700
H	-8.88746800	-6.96179700	0.51524700
H	-9.04111900	-5.31202200	-0.13166800
H	-2.68333700	6.79385500	-1.87691700
H	-6.61175200	5.78076400	0.03966300
H	0.29814700	0.97679600	-8.74288100
H	-0.20081800	1.63825500	-7.17532600
H	-0.44491600	7.98065900	1.77149800
H	-0.07680200	6.23424300	1.73201400
H	-3.27549700	8.47654900	-1.76612100
H	-6.83024500	5.56725400	-1.69221000
H	-13.75482200	3.28574300	2.75772700
H	0.53559800	-0.81559100	8.68108500
H	1.01992900	-2.48902900	8.37497600
H	-8.04708500	-6.52188000	-0.99002000
H	0.90129700	-7.37118100	2.15104000
H	-6.97959300	0.44569800	-4.56030700
H	-3.44353300	1.39770600	-5.37759000
H	8.62175400	0.54423500	-1.85510400
H	-8.23459000	2.86496600	1.16735200

TS4 (12.0 kcal/mol)

C	0.58385400	0.10500500	7.95057600
C	0.50927700	-1.23939800	7.21708900
C	0.77352500	-2.39251900	8.20110900
C	1.48286900	-1.26050100	6.03502400
C	0.63529100	-3.78971000	7.59405400
C	4.51010100	0.33288300	5.41124500
O	4.99869500	-0.12184700	6.44409700
N	5.02868800	0.19869100	4.17980200
C	6.24619400	-0.56772200	4.02635100
C	6.53421500	-0.92585000	2.56544600
C	6.83836700	0.30122400	1.69823600
C	7.57168600	0.01986800	0.39203000
N	6.70611900	-0.60229400	-0.61698100
C	7.11242900	-0.72355900	-1.90046700
N	8.33462900	-0.33750400	-2.24190900
N	6.28312800	-1.31314100	-2.78421200
C	6.23874100	5.15354300	1.19537600
C	4.82349200	5.53517300	1.60557500
O	4.01917100	4.71854100	2.05217500
C	6.42535600	3.63865900	0.99336300
C	6.13141700	3.15092800	-0.40877700
C	7.16783000	3.05681500	-1.34603300
C	4.84179000	2.75456300	-0.78835500
C	6.92814100	2.58371400	-2.63664100
C	4.59925700	2.28402500	-2.07922500
C	5.63828200	2.20333400	-3.00912600
N	4.53686400	6.86526300	1.55225900
C	3.21239800	7.37135100	1.88490200
C	2.46739500	7.73754200	0.60806000
O	2.75603100	8.76783900	-0.00514600
N	1.53667700	6.85253200	0.19836400
C	0.82709900	7.10467700	-1.03924900
C	13.01575900	2.73015200	-2.83279500
C	11.78080500	2.81778000	-1.93134400
C	10.68096000	1.77082700	-2.23521500
O	10.55134600	1.34789000	-3.40319700
O	9.95569500	1.45483400	-1.22715200
C	8.37742300	-6.10335300	-0.75612800
C	6.26160500	-4.28287500	-0.97745300
S	7.43236200	-4.92073900	0.27076800
C	-1.69248500	-6.63171800	-2.24210000
C	-1.90145400	-5.24440600	-2.80358500
C	-3.15807100	-4.82234900	-3.25476900
C	-0.82163400	-4.35607100	-2.89317500

C	-3.33266600	-3.54874600	-3.80333400
C	-0.99419300	-3.08791700	-3.44610100
C	-2.24574900	-2.68132900	-3.91188500
C	-13.99463800	0.62471700	-0.95418800
C	-12.72097700	0.37526100	-0.17114700
C	-11.68024600	1.31309700	-0.13683500
C	-12.52069500	-0.82487200	0.52356700
C	-10.49052500	1.07382300	0.55090100
C	-11.34159300	-1.08159100	1.21782000
C	-10.30495200	-0.13459400	1.24322900
O	-9.18831100	-0.43238000	1.93959300
C	-0.49766000	-0.92508100	-8.17500800
C	-0.71650100	-0.48914400	-6.72157100
C	-0.38072400	0.98582500	-6.43198700
C	-0.69512300	1.32641800	-4.96967900
C	1.07106500	1.34254100	-6.77780200
N	-2.41424500	4.77747900	1.31536000
N	-3.81487200	3.29597400	0.15730900
N	-4.75264700	4.91829200	1.46739100
N	-3.64368800	-6.62970600	0.39126900
N	-2.66353200	-4.69924400	0.82049100
N	-5.65291100	0.20854600	0.15244900
N	-4.15884400	-0.01905700	-5.02831500
N	-4.75654100	1.44395900	-3.46615300
N	-0.98676400	0.13904900	2.56481700
N	1.48722900	-0.87537900	1.86906700
N	-0.32349300	2.31910800	2.01015500
N	1.98310100	2.74927900	1.72592900
C	-9.68954500	3.86754700	3.09715100
C	-8.83908100	2.63796100	3.42138600
C	-7.58938300	2.53250900	2.52719700
C	-8.71965100	0.69023000	-3.74518400
C	-8.56537600	1.29338900	-2.33261400
C	-7.44155100	2.35044200	-2.23849000
C	-1.48984100	6.97885800	2.16498600
C	-2.07703600	5.59139100	2.49148500
C	-3.64234700	4.32178900	0.99896800
C	2.46156400	-4.56632100	-4.77335600
C	3.61289900	-3.90993800	-3.99807900
C	3.19939200	-3.37259100	-2.60518100
C	-2.11627600	-8.35191200	1.45418700
C	-2.51469900	-6.94192700	1.13687900
C	-3.67917300	-5.27603000	0.21586400
C	-1.92963600	-5.72222900	1.39240800
C	-6.48300100	-1.74200700	-1.19105500
C	-6.25089900	-1.15648700	0.19840700
C	-5.38868500	0.94320900	-7.03052400
C	-4.97833600	0.94829700	-5.59280300
C	-4.06220600	0.31113300	-3.74541900
C	-5.35761700	1.85609800	-4.63385100
C	-2.36487700	0.57247400	2.72039400
C	-0.64569100	-1.16885900	2.93866000
C	-1.54235400	-2.00071800	3.60944800
C	-1.19761000	-3.30706000	3.95646900
C	-2.23400600	-4.14034200	4.67649800
C	0.07003300	-3.83129100	3.61679700
C	0.45633100	-5.18917000	4.16902500
C	0.95300300	-3.04420300	2.84428500
C	0.59915600	-1.71050500	2.57926900
C	0.00256500	1.03148700	2.17810200
C	1.29116300	0.52230500	1.98470300
C	2.37849400	1.42038600	1.85135700
C	0.66643900	3.18453400	1.67798000
C	-1.80786000	0.08932500	-0.51626700

C	-1.69043600	-1.31520200	-0.16457700
C	-0.50971300	-1.99533000	-0.34531700
C	-0.61787900	0.72142100	-1.00776500
C	0.56373500	0.03753500	-1.15743700
C	0.68300900	-1.34529400	-0.80856400
C	2.89820400	-0.68471900	-4.21130100
C	2.09988900	-1.39082500	0.76391500
C	2.53062600	-2.82886100	0.88542900
C	2.32461400	-3.47188300	2.28645800
C	2.50659800	-4.98126000	2.02863500
C	3.41878000	-2.98967400	3.26379900
O	-7.35149900	1.39379200	1.99585100
O	-6.89271400	3.56342500	2.41711700
O	-7.52825200	3.34427100	-2.96856700
O	-6.46505100	2.11447700	-1.41218200
O	3.81828600	-2.34160700	-2.16059200
O	2.29403200	-3.99109700	-2.00725700
O	3.59870000	1.11944800	1.81586200
O	0.43144300	4.37833700	1.35222800
O	-2.91386800	0.72709100	-0.38800800
O	-2.81854700	-1.88773800	0.35291500
O	1.80374700	-1.02166100	-3.98746600
O	3.96354300	-0.30125900	-4.51044800
H	-0.55960400	6.87339700	1.60689700
H	-2.18915800	7.57944700	1.57398500
H	-1.33922800	5.01784100	3.05730000
H	-2.96738400	5.68629500	3.12019700
H	-1.59571300	4.29223200	0.94702500
H	-3.14050400	2.53773300	-0.01672500
H	-4.74824100	3.14139700	-0.22435100
H	-4.64829700	5.74154200	2.03631100
H	-5.58714000	4.32560700	1.65990200
H	1.66733200	-3.84328900	-4.98064700
H	2.80770700	-4.98132600	-5.72695900
H	4.41250700	-4.64568200	-3.82819000
H	4.07477000	-3.10328800	-4.57884700
H	-1.21767900	-8.34517500	2.07498100
H	-1.88964700	-8.91921700	0.54352200
H	-1.02732900	-5.50952400	1.94150200
H	-4.43351800	-4.77193800	-0.36957600
H	-5.53907200	-1.86194200	-1.73339500
H	-7.14141800	-1.10760900	-1.78747500
H	-5.57092500	-1.78916800	0.77476200
H	-7.19632700	-1.07626100	0.73850300
H	-6.09471000	0.84702200	-0.59921300
H	-5.91666100	0.72176800	1.01513500
H	-5.91085000	0.01528700	-7.29417500
H	-6.06017100	1.78155700	-7.23711300
H	-6.02001500	2.70647300	-4.64156900
H	-3.49845000	-0.21815100	-2.99080600
H	-2.46587300	1.54539900	2.25066600
H	-3.02289100	-0.13828900	2.21852200
H	-2.52921000	-1.63650700	3.86046300
H	-1.93647200	-4.38871900	5.70223400
H	-3.18089600	-3.59818500	4.73379600
H	-2.42663000	-5.08368100	4.15402100
H	1.47067400	-5.17511800	4.57179300
H	-0.20566900	-5.47130800	4.98731200
H	0.41814300	-5.99661200	3.42997400
H	-4.61805500	0.19637300	0.00979100
H	-2.63133400	-2.82654700	0.58785000
H	-0.47997000	-3.06019200	-0.13908700
H	-0.69295800	1.77468100	-1.25974900
H	1.43559000	0.55161800	-1.55431700

H	-4.27952100	-7.29354900	-0.02198200
H	-5.02643200	1.81150100	-2.55039000
H	2.72009600	3.45274400	1.69188300
H	-2.89990100	-8.88894800	2.00183400
H	2.01928000	-5.36503600	-4.17495300
H	-1.27522800	7.51127000	3.09766800
H	-4.52464200	1.02426600	-7.70224100
H	-6.95260900	-2.72670400	-1.09823100
H	-2.63574400	0.65767700	3.78013100
H	2.78739500	-0.70659100	0.28552600
H	2.06192800	-3.43384900	0.10586700
H	3.59693700	-2.87545300	0.63990500
H	2.67552700	-5.55936000	2.93718600
H	1.65003100	-5.39938700	1.49162500
H	3.38542800	-5.11636600	1.39052900
H	3.27068700	-3.41809200	4.26051000
H	4.40587600	-3.30194500	2.90523100
H	3.41809400	-1.90231700	3.36187100
H	-8.47449200	2.70999700	4.45499500
H	-9.42859100	1.72074200	3.35394000
H	-10.13548300	3.78045500	2.10109500
H	-9.06338800	4.76298800	3.10930600
H	-8.42309200	0.50759700	-1.58552000
H	-9.49991800	1.80041900	-2.07043300
H	-9.57356500	0.00560300	-3.78645300
H	-7.81866200	0.14526000	-4.04608200
H	1.39633600	-1.95147300	-1.36009000
H	-8.87175300	1.49633200	-4.46781200
H	-10.50551800	3.99196100	3.81783300
H	-9.68958400	1.80449200	0.53832100
H	-11.20034100	-2.01343700	1.75660600
H	-13.30724700	-1.57734700	0.52200100
H	-11.79291800	2.25541100	-0.66844200
H	-14.27371800	1.68358300	-0.93698500
H	-4.00520800	-5.50397800	-3.18807800
H	-4.30687700	-3.22605400	-4.15955100
H	-2.38499200	-1.70740500	-4.36595100
H	-0.14656800	-2.41550900	-3.49850700
H	0.16459600	-4.63554600	-2.53151500
H	-1.76409700	-0.65604000	-6.44556400
H	-0.11668000	-1.12487700	-6.05631400
H	-1.03464800	1.60205000	-7.06901200
H	-0.09085200	0.72225100	-4.28483900
H	-0.48923900	2.38150500	-4.75539800
H	-1.74563100	1.13240200	-4.74376000
H	1.77287100	0.71242800	-6.21997800
H	1.28937900	1.21274600	-7.84277800
H	1.29046000	2.38429600	-6.51937600
H	1.37585000	5.96335800	0.68148400
H	5.13918100	7.47591300	1.01895000
H	7.76303100	2.47206200	-3.32193000
H	5.44222200	1.81757000	-4.00407000
H	3.59728300	1.96753300	-2.35609700
H	4.04254100	2.77440100	-0.05889900
H	7.46197200	3.38842100	1.23885700
H	5.78012300	3.12496600	1.71010400
H	12.04896300	2.73192500	-0.87330700
H	11.31180500	3.80577900	-2.04741100
H	5.58140400	-5.06534200	-1.32262400
H	5.66907300	-3.49694400	-0.50935000
H	6.79135100	-3.84105600	-1.82349600
H	5.70605100	-0.57401400	-0.47209400
H	5.34093800	-1.68271000	-2.52851500
H	6.41272500	-1.08177000	-3.75586500
H	8.94582400	0.38992400	-1.72306300
H	8.62814000	-0.43541300	-3.20356600
H	8.42758300	-0.64361700	0.56478300
H	7.97230100	0.95594300	0.00036900
H	5.91741200	0.85202100	1.48674100
H	7.48172900	0.98023000	2.27118300
H	7.39651100	-1.60438400	2.55232300
H	5.68953900	-1.48763900	2.15163700
H	4.51850200	0.55047700	3.36315500
H	0.76014000	-4.56916300	8.35386900
H	-0.35192400	-3.92018600	7.13819200
H	1.37997800	-3.96515600	6.81360500
H	1.36987500	-2.16278100	5.43279200
H	1.30562600	-0.41192200	5.36644300
H	2.51837000	-1.20386000	6.38770400
H	1.78394000	-2.27241800	8.61669300
H	0.08025700	-2.29807900	9.04858400
H	-0.50956300	-1.36258700	6.81951100
H	-14.83154500	0.04978300	-0.54393100
H	-13.88730400	0.33741100	-2.00847300
H	1.48168300	6.99130600	-1.91105700
H	0.55750800	-0.87666000	-8.46491000
H	1.59645600	0.27525700	8.33566500
H	6.15774700	-1.47680900	4.63149300
H	13.56507200	1.79867300	-2.65567300
H	12.70362400	2.72668600	-3.88041700
H	-2.62816100	-7.19904400	-2.21636400
H	-1.28938400	-6.59070700	-1.22578000
H	9.13000800	-6.56314700	-0.11173900
H	7.72663800	-6.88538300	-1.15805000
H	2.70740100	6.59169100	2.45622400
H	6.53471400	5.71064000	0.29864300
H	-0.11140000	0.14346700	8.79764500
H	0.34285900	0.93473300	7.27778800
H	0.43420200	8.12452200	-1.04641600
H	0.00299400	6.39375500	-1.11677800
H	3.30684900	8.27521500	2.49338300
H	6.89136000	5.50276100	2.00691400
H	13.70386800	3.56702100	-2.66368300
H	-1.06074500	-0.28610000	-8.86670900
H	-0.83409600	-1.95601300	-8.32767300
H	8.88100400	-5.58875500	-1.57931200
H	-0.97670000	-7.19793200	-2.85006700
H	7.09669300	-0.00594400	4.43913600
H	3.56408200	0.90750300	5.40872400
H	-8.51623100	0.32938100	1.95335700
H	8.18908200	3.27787300	-1.05106400

Int4 (2.4 kcal/mol)

C	0.60342200	0.31453300	7.97330300
C	0.51844100	-1.04575600	7.26926600
C	0.81689400	-2.17543500	8.27071000
C	1.45986400	-1.08913300	6.06067700
C	0.65922100	-3.58733300	7.70386500
C	4.53184000	0.47268300	5.43204000
O	5.01342500	0.03586800	6.47466900
N	5.04662200	0.29598900	4.20344900
C	6.26964600	-0.46577000	4.07467500
C	6.56370300	-0.86542600	2.62519900
C	6.87883200	0.33615400	1.72580100
C	7.59170800	0.01630400	0.41614700
N	6.69512300	-0.60354500	-0.56763200
C	7.08751600	-0.77298100	-1.85510200

N	8.31602300	-0.43224100	-2.21760600
N	6.22576200	-1.35758100	-2.70737100
C	6.26182600	5.17322900	1.08318600
C	4.84871700	5.56442000	1.49225500
O	4.05195300	4.76038700	1.97672400
C	6.44983000	3.65509000	0.91651500
C	6.14320700	3.13404200	-0.47052400
C	7.17676100	2.97940600	-1.40234300
C	4.84099400	2.77223100	-0.84255200
C	6.92194000	2.47715100	-2.67923500
C	4.58365700	2.26780000	-2.11755600
C	5.62172400	2.12376200	-3.04115300
N	4.55785300	6.88943900	1.39147600
C	3.23373300	7.40800600	1.70708100
C	2.49694800	7.73860100	0.41576700
O	2.79993100	8.74344300	-0.23125100
N	1.55983300	6.84804400	0.03547900
C	0.85121300	7.05754900	-1.21047400
C	13.04371300	2.64062300	-2.86889000
C	11.80954700	2.70086900	-1.96271800
C	10.71557000	1.64801800	-2.26970600
O	10.61463000	1.18873100	-3.42478700
O	9.95955600	1.36617600	-1.27145300
C	8.40800400	-6.13316000	-0.54759100
C	6.35917200	-4.22414300	-0.75571600
S	7.67543500	-4.74903600	0.39777200
C	-1.66028200	-6.70865200	-2.02701400
C	-2.04567400	-5.41672600	-2.70697100
C	-3.34900300	-5.21555500	-3.17535900
C	-1.10029400	-4.39660400	-2.88795800
C	-3.70240500	-4.03120000	-3.82563000
C	-1.45398200	-3.21562300	-3.54133900
C	-2.75254400	-3.02836500	-4.02303800
C	-13.96728700	0.57464300	-0.95552900
C	-12.72328300	0.36257800	-0.11537100
C	-11.63552500	1.24447300	-0.17083800
C	-12.59841100	-0.75161000	0.72503100
C	-10.47085400	1.03161100	0.56693000
C	-11.44571200	-0.98064700	1.47140600
C	-10.36007800	-0.09177800	1.40370900
O	-9.26835400	-0.36226400	2.14806100
C	-0.46301600	-1.17134000	-8.11766300
C	-0.59755900	-0.57397600	-6.71283100
C	-0.14463800	0.89223800	-6.59363400
C	-0.40071300	1.41033600	-5.17286400
C	1.32097700	1.09949700	-6.99894600
N	-2.46382600	4.82587100	1.18540400
N	-3.91987400	3.37709700	0.05703500
N	-4.79810900	5.06515900	1.33287700
N	-3.60692000	-6.62230800	0.60071700
N	-2.60076200	-4.68656400	0.95239000
N	-5.75175200	0.24323400	0.16538400
N	-3.88628700	0.00923200	-5.09415300
N	-4.64014400	1.35304900	-3.49758200
N	-0.96160100	0.18894100	2.50860600
N	1.52823100	-0.81853400	1.76398000
N	-0.30559000	2.36137700	1.93737700
N	2.00595900	2.80449700	1.68650000
C	-9.66750600	3.93294500	3.00638500
C	-8.79091000	2.76835700	3.46952300
C	-7.56841900	2.58467200	2.55402900
C	-8.68981800	0.56409900	-3.74258000
C	-8.55020400	1.22946100	-2.35966000
C	-7.45084400	2.31738600	-2.32103400
C	-1.46855900	7.02106200	1.99397000
C	-2.09291300	5.65244400	2.34219700
C	-3.71265100	4.40708300	0.87505500
C	2.49500400	-4.71341500	-4.61189400
C	3.61728800	-4.05186900	-3.79499400
C	3.17818200	-3.47142500	-2.42372400
C	-2.08652800	-8.32399800	1.71605200
C	-2.47361600	-6.92247200	1.34454800
C	-3.62893900	-5.27366600	0.37967000
C	-1.87270600	-5.70059700	1.54856200
C	-6.45423200	-1.79381000	-1.11876300
C	-6.31608300	-1.12916500	0.24757200
C	-5.35602000	0.72593400	-7.03079000
C	-4.88576500	0.81848300	-5.61275800
C	-3.77497600	0.35711000	-3.81679200
C	-5.36013600	1.65695400	-4.63251100
C	-2.34081000	0.63246500	2.72938400
C	-0.60234300	-1.11347300	2.85475400
C	-1.49015900	-1.94525700	3.54357600
C	-1.12165800	-3.22144700	3.95091100
C	-2.14018500	-4.04492300	4.70371700
C	0.16284400	-3.73279600	3.65084200
C	0.56745800	-5.05272200	4.27496000
C	1.02854600	-2.97593300	2.83539600
C	0.64758300	-1.66047100	2.50827100
C	0.01071200	1.07347900	2.08098100
C	1.30599600	0.57378800	1.85765700
C	2.40037400	1.48184200	1.77132600
C	0.68198200	3.23611100	1.61951900
C	-2.06471600	0.20262500	-0.54984800
C	-1.89397300	-1.17801500	-0.20560200
C	-0.72505700	-1.86069000	-0.52476100
C	-0.96861800	0.82094600	-1.19608800
C	0.20525600	0.13042300	-1.50214700
C	0.33395500	-1.22284400	-1.18625900
C	2.76413700	-0.88440900	-4.18075800
C	2.43520300	-1.31988300	0.96955300
C	2.72325000	-2.75729500	0.98508400
C	2.40219300	-3.45810100	2.32493900
C	2.47811500	-4.96213700	1.98519200
C	3.47624300	-3.08643300	3.37079400
O	-7.39587600	1.42348900	2.04605600
O	-6.83617000	3.58224400	2.39135000
O	-7.57994900	3.28094200	-3.08899800
O	-6.46244900	2.12963800	-1.50581800
O	3.79922900	-2.42902400	-2.00743200
O	2.27234600	-4.07135200	-1.80559500
O	3.61749700	1.17683200	1.77692500
O	0.45364000	4.42705700	1.30663100
O	-3.16596300	0.85688900	-0.26149300
O	-2.92337800	-1.78416700	0.48211000
O	1.72361200	-1.40288300	-4.08813000
O	3.78603300	-0.33392300	-4.34013400
H	-0.54398200	6.88409400	1.43401800
H	-2.15420600	7.63222900	1.39786800
H	-1.36838400	5.07184400	2.91764100
H	-2.97702800	5.78413500	2.97324300
H	-1.67934700	4.29564500	0.81457800
H	-3.31987600	2.52110100	-0.05140800
H	-4.86673200	3.24591200	-0.30315000
H	-4.65485500	5.88580900	1.89756400
H	-5.62274700	4.48240000	1.56855400
H	1.73154400	-3.98146800	-4.88942500
H	2.88654300	-5.17186400	-5.52687200

H	4.39978600	-4.79412700	-3.57862000
H	4.11233700	-3.26331100	-4.37397500
H	-1.17715500	-8.30187400	2.32074500
H	-1.88264800	-8.93341700	0.82751500
H	-0.95728000	-5.48443200	2.07354300
H	-4.38506800	-4.78285100	-0.21499300
H	-5.47787600	-1.89941200	-1.60349100
H	-7.10472400	-1.21759200	-1.78066300
H	-5.65044900	-1.71472300	0.88751100
H	-7.28947700	-1.05498900	0.73883000
H	-6.16589500	0.84624300	-0.61723700
H	-6.04401000	0.77640000	1.00717800
H	-5.72700300	-0.27987900	-7.26319000
H	-6.16700100	1.43766800	-7.21032900
H	-6.13405400	2.40803200	-4.61012400
H	-3.08686900	-0.06895700	-3.10047400
H	-2.44472700	1.61666000	2.28641200
H	-3.01922200	-0.05297800	2.22291000
H	-2.48384800	-1.58879800	3.77244500
H	-1.83915000	-4.22930700	5.74141700
H	-3.10263200	-3.52931800	4.72529200
H	-2.29642200	-5.01812300	4.22738600
H	1.61221800	-5.04336100	4.58607500
H	-0.02766600	-5.24673900	5.16721400
H	0.43588900	-5.91244800	3.60873900
H	-4.69823400	0.27227500	0.04249900
H	-2.66895900	-2.70969800	0.68233000
H	-0.64926700	-2.91521500	-0.27579800
H	-1.07704500	1.87044800	-1.45587500
H	1.01421800	0.65243200	-2.00854700
H	-4.25395900	-7.29125700	0.21383700
H	-4.88459200	1.71704300	-2.57632100
H	2.74176600	3.51231200	1.67471500
H	-2.86703600	-8.82670900	2.29959100
H	2.00293900	-5.48028900	-4.00921700
H	-1.23638700	7.55909100	2.91927900
H	-4.54859400	0.93992800	-7.74247000
H	-6.88854400	-2.79225700	-1.00058400
H	-2.55646400	0.68661800	3.80244700
H	2.97377500	-0.63213800	0.33770600
H	2.20921500	-3.22858000	0.12574700
H	3.77921400	-2.89948700	0.73566100
H	2.58908200	-5.60212800	2.85939100
H	1.60060800	-5.27887300	1.41457800
H	3.35141300	-5.12772500	1.34758100
H	3.26075000	-3.53884700	4.34352400
H	4.46000500	-3.43939900	3.04223100
H	3.53424700	-2.00395400	3.51672200
H	-8.40590100	2.97953400	4.47573200
H	-9.36544300	1.84046400	3.52548300
H	-10.12622100	3.71185500	2.03728900
H	-9.05664000	4.83246400	2.89556800
H	-8.38388000	0.47818100	-1.58217700
H	-9.49695400	1.72355600	-2.11651800
H	-9.52096100	-0.14971100	-3.75420800
H	-7.77232400	0.03668600	-4.02396600
H	1.20706000	-1.79296800	-1.47673300
H	-8.87082700	1.33382700	-4.49788700
H	-10.47549400	4.13716900	3.71814200
H	-9.63167600	1.71361400	0.48512800
H	-11.36224500	-1.84577800	2.12203800
H	-13.42358500	-1.45811300	0.79687000
H	-11.68993500	2.11886500	-0.81540800
H	-14.17079600	1.64007600	-1.10590700
H	-4.09160100	-6.00046600	-3.03631100
H	-4.71875100	-3.88986700	-4.18413900
H	-3.02818300	-2.11593300	-4.54387400
H	-0.71473600	-2.43017200	-3.65256600
H	-0.08507500	-4.51220000	-2.51299000
H	-1.64318900	-0.63102400	-6.38836000
H	-0.02043900	-1.18075700	-6.00318800
H	-0.76493400	1.48413900	-7.28522200
H	0.15613700	0.82354400	-4.43477100
H	-0.09707100	2.45871600	-5.06893400
H	-1.45903100	1.32970600	-4.91454200
H	1.99398900	0.50672500	-6.36863400
H	1.50734000	0.81156200	-8.03875100
H	1.61382800	2.14949800	-6.88679900
H	1.39214400	5.98178900	0.55401500
H	5.15586000	7.48045300	0.83153600
H	7.75277900	2.32441000	-3.36144600
H	5.41309200	1.71254200	-4.02299500
H	3.57394300	1.97185800	-2.38699200
H	4.03912300	2.85392600	-0.12047900
H	7.48882500	3.41176100	1.15863300
H	5.81161800	3.15934700	1.65210900
H	12.08317600	2.60545200	-0.90665500
H	11.32911800	3.68521400	-2.06120400
H	5.58226700	-4.98558600	-0.85829200
H	5.91245900	-3.31843600	-0.34614400
H	6.77155500	-3.97227700	-1.73462900
H	5.70139400	-0.46648400	-0.44045300
H	5.29396800	-1.73855400	-2.42459600
H	6.35129400	-1.17255500	-3.68921000
H	8.95185800	0.30855600	-1.73485800
H	8.60315400	-0.59648000	-3.17220300
H	8.43146400	-0.66692600	0.58792100
H	8.01219400	0.93589500	0.00616800
H	5.96567500	0.89911000	1.51050500
H	7.53842300	1.01691800	2.27772600
H	7.42413000	-1.54610700	2.63545100
H	5.71994300	-1.43793800	2.22269000
H	4.55986900	0.65907400	3.38084000
H	0.81778600	-4.34722800	8.47680000
H	-0.34656400	-3.73208100	7.29416700
H	1.37230600	-3.78075600	6.89848200
H	1.33839400	-2.00763700	5.48357100
H	1.26072900	-0.25637200	5.37796000
H	2.50347400	-1.01970400	6.38598900
H	1.84047100	-2.04522600	8.64903400
H	0.15249800	-2.06064600	9.13815100
H	-0.51073200	-1.18278100	6.90369900
H	-14.84790300	0.12760900	-0.48182600
H	-13.86818000	0.12248100	-1.95125800
H	1.49933800	6.88765800	-2.07785500
H	0.58168000	-1.24497500	-8.43930100
H	1.62374700	0.49744100	8.33111000
H	6.18354100	-1.35650500	4.70620400
H	13.60367400	1.71288500	-2.70720000
H	12.73119700	2.64965600	-3.91636700
H	-2.51682900	-7.38427100	-1.93706700
H	-1.26140600	-6.52671500	-1.02486100
H	9.22618100	-6.53686000	0.05287400
H	7.67340000	-6.92367400	-0.72591600
H	2.72464700	6.64668800	2.29901300
H	6.55220300	5.70853500	0.17169100
H	-0.07188900	0.36510400	8.83527200
H	0.34125000	1.12955400	7.29058600

H	0.48266200	8.08487300	-1.26337500
H	0.01018700	6.36372700	-1.25273400
H	3.32902300	8.32839300	2.29007600
H	6.91836200	5.54263800	1.88261100
H	13.72147200	3.48308300	-2.68716800
H	-0.99550600	-0.56175400	-8.85865200
H	-0.88840400	-2.17992700	-8.15285200
H	8.80766800	-5.78343800	-1.50353200
H	-0.88011300	-7.23414300	-2.59106500
H	7.11560300	0.11260400	4.47320600
H	3.59430800	1.06133200	5.41529000
H	-8.56938400	0.37366800	2.09729300
H	8.20471700	3.17832600	-1.11460200

E:P (-6.8 kcal/mol)

C	0.74164100	1.03907200	7.80304800
C	0.64672100	-0.37408500	7.21487500
C	0.97467800	-1.41749600	8.29716000
C	1.55913300	-0.51320500	5.99140400
C	0.82264600	-2.87137600	7.84717200
C	4.65199900	0.97185900	5.23092100
O	5.13703400	0.62577500	6.30584600
N	5.16116100	0.68844400	4.02089200
C	6.38226400	-0.08266300	3.95034400
C	6.66826400	-0.59171900	2.53225400
C	6.93952900	0.54005200	1.53316800
C	7.62523400	0.13398500	0.23182100
N	6.72259200	-0.56869700	-0.68531100
C	7.12051600	-0.91618400	-1.93341900
N	8.37708400	-0.67429400	-2.30187200
N	6.23888600	-1.54085900	-2.72753700
C	6.34253500	5.26179800	0.46157600
C	4.94113800	5.69337000	0.87022700
O	4.16766000	4.94707400	1.47176600
C	6.51653900	3.73560700	0.40180600
C	6.10426300	3.09616000	-0.90696600
C	7.07139600	2.77892800	-1.86899500
C	4.76606600	2.77040800	-1.16613300
C	6.71277600	2.14120900	-3.05726200
C	4.40504300	2.13353100	-2.35340500
C	5.37852400	1.81581800	-3.30282000
N	4.63665000	6.99564200	0.62289300
C	3.31424400	7.53960000	0.90188000
C	2.57112000	7.74772500	-0.41067300
O	2.87810900	8.68184700	-1.15459500
N	1.62570900	6.83118200	-0.69736700
C	0.91236100	6.92296900	-1.95506500
C	13.10180000	2.39116200	-3.29257200
C	11.84428600	2.52204400	-2.42606700
C	10.79847000	1.39080000	-2.59467100
O	10.81507900	0.68582300	-3.62128500
O	9.95579400	1.30671900	-1.62795300
C	8.50230900	-6.14407000	-0.15309900
C	6.67444500	-4.03040400	-0.27119100
S	6.80472000	-5.79250100	-0.73061600
C	-1.57692800	-6.86403200	-1.50418900
C	-1.97892200	-5.62328800	-2.26574500
C	-3.29309400	-5.44851300	-2.71468700
C	-1.03722200	-4.62045600	-2.53682000
C	-3.66222300	-4.30728700	-3.43092900
C	-1.40876000	-3.48265500	-3.25347500
C	-2.71817500	-3.31917500	-3.71364900
C	-13.89071500	0.46893600	-1.00971900
C	-12.64039000	0.33551200	-0.16255900
C	-11.55598500	1.21244900	-0.30438300
C	-12.50499700	-0.69900300	0.77235900
C	-10.38467800	1.06880800	0.43934900
C	-11.34542200	-0.85789500	1.52665300
C	-10.26319200	0.02373600	1.37091900
O	-9.16386400	-0.17777200	2.12606300
C	-0.43331900	-1.89942200	-8.07980200
C	-0.74827800	-1.42948200	-6.65666600
C	0.28381700	-0.43266700	-6.09525700
C	-0.30057700	0.35790300	-4.92176600
C	1.58460800	-1.13235000	-5.67403200
N	-2.38362700	4.90782000	0.67843400
N	-3.85708300	3.36780400	-0.29645600
N	-4.71553800	5.17692200	0.81575700
N	-3.51126000	-6.54048300	1.12925400
N	-2.49080100	-4.58503300	1.26929700
N	-5.66513700	0.25540400	0.07129600
N	-3.86447600	-0.35798800	-5.11107200
N	-4.66675300	1.15395100	-3.69814300
N	-0.85831700	0.42075100	2.40187900
N	1.62779600	-0.64531200	1.74166100
N	-0.20006200	2.54172600	1.66406800
N	2.11152600	2.96231600	1.38493300
C	-9.57061500	4.17830000	2.60136800
C	-8.69142400	3.05563700	3.15455800
C	-7.47152700	2.79654000	2.25386000
C	-8.63256200	0.21349500	-3.82149400
C	-8.51347300	0.99533400	-2.49915500
C	-7.43728600	2.10499100	-2.54983000
C	-1.38518800	7.17349800	1.25574700
C	-2.00156100	5.84478300	1.74374300
C	-3.63746700	4.47007400	0.41676100
C	2.55656800	-5.10399700	-4.28781200
C	3.51899400	-4.06139900	-3.69749800
C	3.21364100	-3.58712900	-2.25624800
C	-1.97374200	-8.13447400	2.37288100
C	-2.36275200	-6.77330200	1.87414200
C	-3.53384800	-5.21918000	0.77980100
C	-1.75323800	-5.54069900	1.94511500
C	-6.37416500	-1.89403300	-1.01074000
C	-6.21966500	-1.10723500	0.28677200
C	-5.32200900	0.08157400	-7.13467700
C	-4.87608300	0.35718900	-5.73336100
C	-3.77702100	0.14616100	-3.88582100
C	-5.38136400	1.29761400	-4.86789000
C	-2.23918600	0.87666900	2.57275000
C	-0.50173300	-0.85048800	2.85195300
C	-1.39116400	-1.62399700	3.60300100
C	-1.02082700	-2.86047200	4.11746400
C	-2.04019200	-3.61822300	4.93541300
C	0.26579100	-3.39247400	3.86582100
C	0.67241600	-4.65100100	4.60594800
C	1.13126500	-2.70598100	2.99039100
C	0.74827400	-1.42340500	2.55349700
C	0.11565800	1.26931000	1.91158500
C	1.41041700	0.75144600	1.72977900
C	2.50351900	1.64791200	1.56391000
C	0.78767700	3.38791800	1.27722000
C	-1.95930800	0.18912600	-0.62611500
C	-1.78215300	-1.16937200	-0.20292700
C	-0.60740400	-1.86053700	-0.47648000
C	-0.85513300	0.78524700	-1.27843100
C	0.32876000	0.08993000	-1.53028200

C	0.45557300	-1.24852100	-1.15848300
C	2.52302600	-1.21465500	0.98000100
C	2.79785900	-2.65028300	1.10935700
C	2.50281000	-3.22547700	2.51329700
C	2.58838300	-4.75405900	2.31771400
C	3.58630900	-2.74990000	3.50579100
O	-7.29319500	1.59410400	1.85543700
O	-6.74553100	3.77879500	1.99771500
O	-7.58360500	3.00215800	-3.39078200
O	-6.44286700	1.99942200	-1.72539800
O	4.02176800	-2.71548700	-1.76533700
O	2.22488300	-4.06247700	-1.65910600
O	3.72011400	1.34113600	1.57965500
O	0.56094800	4.54802400	0.86458300
O	-3.07400400	0.84347600	-0.39829000
O	-2.81535700	-1.74166100	0.50642400
H	-0.46694900	6.98513900	0.70051500
H	-2.07985500	7.72363400	0.61240100
H	-1.26814500	5.32470800	2.36337800
H	-2.87828200	6.03680800	2.36985900
H	-1.60662300	4.33470000	0.36101300
H	-3.25720500	2.50336200	-0.33592300
H	-4.80971600	3.20899800	-0.62811200
H	-4.56319600	6.04561100	1.30037900
H	-5.54181900	4.62294600	1.10840800
H	1.53774100	-4.71036600	-4.33605200
H	2.86634200	-5.39315400	-5.29826800
H	4.54840200	-4.44547700	-3.69385300
H	3.53953900	-3.16278700	-4.32811500
H	-1.05491100	-8.05788400	2.95855600
H	-1.78520200	-8.82786100	1.54455300
H	-0.82546200	-5.28045600	2.42663000
H	-4.30156000	-4.78399300	0.15736300
H	-5.40379900	-2.04508100	-1.49541100
H	-7.03273700	-1.37995900	-1.71462500
H	-5.54076500	-1.62854600	0.96700900
H	-7.18602900	-0.99241200	0.78403500
H	-6.09727300	0.78207300	-0.75420000
H	-5.95091700	0.86178900	0.86493200
H	-5.65177600	-0.95816900	-7.25026400
H	-6.15620300	0.73529700	-7.40597300
H	-6.17410400	2.02467500	-4.94293100
H	-3.09005900	-0.17161900	-3.11444800
H	-2.33802600	1.82675900	2.06013400
H	-2.91272200	0.15671100	2.10940500
H	-2.38624700	-1.25189600	3.79873200
H	-1.75306900	-3.69247200	5.99091500
H	-3.00878300	-3.11569700	4.89089800
H	-2.17740300	-4.63621400	4.55770100
H	1.71272200	-4.60537800	4.92968400
H	0.06683800	-4.77134400	5.50392100
H	0.55730400	-5.56589100	4.01472600
H	-4.61384800	0.27774500	-0.06357100
H	-2.55256800	-2.64596500	0.77924600
H	-0.53011200	-2.90285300	-0.18116200
H	-0.96597500	1.81990200	-1.59138800
H	1.14111600	0.58630500	-2.05535600
H	-4.16987700	-7.24002800	0.82475600
H	-4.94413700	1.60527400	-2.82354300
H	2.84746800	3.66663400	1.31596000
H	-2.74636800	-8.57492300	3.01430900
H	2.52679500	-5.99876000	-3.65968400
H	-1.14281100	7.79906900	2.12151500
H	-4.51356400	0.24126900	-7.85951700
H	-6.80736100	-2.87672600	-0.79607700
H	-2.46784000	1.00795700	3.63650400
H	3.06510400	-0.58094000	0.29657300
H	2.24914000	-3.18502400	0.31162100
H	3.84148700	-2.82753800	0.83515400
H	2.71229800	-5.30514800	3.24874100
H	1.70746900	-5.13161600	1.79091600
H	3.45595600	-4.97571100	1.68919800
H	3.38971300	-3.11601900	4.51799400
H	4.56927100	-3.12096900	3.19517200
H	3.63576000	-1.65843700	3.55294600
H	-8.30273900	3.34829200	4.13879800
H	-9.26509600	2.13547600	3.28902100
H	-10.03193500	3.87941300	1.65467900
H	-8.96136700	5.06666700	2.41640200
H	-8.33166900	0.31685300	-1.66089300
H	-9.47181600	1.48623700	-2.30014400
H	-9.44572500	-0.51933300	-3.77362200
H	-7.70164300	-0.31208400	-4.05804200
H	1.32926900	-1.83246300	-1.41935400
H	-8.83134400	0.91211400	-4.63890400
H	-10.37682100	4.43853200	3.29671500
H	-9.54786800	1.74241200	0.29033900
H	-11.25383900	-1.66225300	2.25009800
H	-13.32685500	-1.39895600	0.91307900
H	-11.61812500	2.02642200	-1.02318000
H	-14.12886100	1.51912500	-1.20952000
H	-4.03329600	-6.22052500	-2.50732600
H	-4.68661700	-4.18834600	-3.77408900
H	-3.00306900	-2.44134900	-4.28710800
H	-0.67117300	-2.70708000	-3.42790700
H	-0.01092300	-4.71226000	-2.18666100
H	-1.74404600	-0.97397700	-6.62830500
H	-0.80868400	-2.29623300	-5.98535400
H	0.52645500	0.28077300	-6.89947300
H	-0.55661400	-0.30401100	-4.09040200
H	0.41890800	1.08978500	-4.53734100
H	-1.21425900	0.88373600	-5.20996100
H	1.39687800	-1.81362600	-4.83605400
H	2.02327100	-1.71843600	-6.48925400
H	2.33643000	-0.40695300	-5.34199600
H	1.45817900	6.02130100	-0.09462500
H	5.21455300	7.51953500	-0.01917900
H	7.49036200	1.86969600	-3.76441600
H	5.09807500	1.29773600	-4.21488800
H	3.36534900	1.87169400	-2.52832300
H	4.01230400	2.98553300	-0.42186100
H	7.57039000	3.50525600	0.58481500
H	5.93533300	3.30760800	1.22235500
H	12.09814600	2.58657800	-1.36212300
H	11.32608900	3.46239000	-2.66311300
H	7.49479900	-3.45842100	-0.71028400
H	5.72945000	-3.66224000	-0.68166400
H	6.68246300	-3.90502600	0.81561100
H	5.72702900	-0.44750200	-0.56063300
H	5.34347100	-1.98928000	-2.38536000
H	6.52810500	-1.74143100	-3.67072900
H	8.99369900	0.14161500	-1.93119900
H	8.66788200	-0.94819600	-3.23013200
H	8.48139400	-0.52076700	0.43485800
H	8.02732800	1.02509500	-0.25277500
H	6.01076600	1.06866100	1.30097400
H	7.60045900	1.27222000	2.01314500
H	7.54773500	-1.24611200	2.58571100

H	5.83523900	-1.21609800	2.18891200
H	4.66740200	0.97848800	3.17283800
H	1.00256200	-3.56584100	8.67502300
H	-0.18787900	-3.05863000	7.46736500
H	1.52451600	-3.12062300	7.04740700
H	1.42181900	-1.47197200	5.48831500
H	1.34719800	0.26654800	5.25226300
H	2.61021200	-0.42459000	6.28683800
H	2.00341100	-1.24827700	8.64499100
H	0.32466100	-1.23985400	9.16495900
H	-0.39018600	-0.54362900	6.88672200
H	-14.75598200	0.01644600	-0.51379000
H	-13.77698700	-0.02542400	-1.98351600
H	1.54840800	6.64194400	-2.80236900
H	0.54175100	-2.39676000	-8.14152300
H	1.77057100	1.25587400	8.11428300
H	6.29887400	-0.92229600	4.64895800
H	13.68996000	1.51474500	-2.99955600
H	12.81802400	2.24995100	-4.33874500
H	-2.42893500	-7.53450200	-1.35303600
H	-1.16325700	-6.61034800	-0.52403300
H	8.71979300	-7.18750100	-0.39278100
H	9.22877700	-5.50395800	-0.66199000
H	2.81196900	6.83377400	1.56432300
H	6.61882700	5.72787400	-0.49134000
H	0.09146600	1.15547300	8.67793100
H	0.45302700	1.79469000	7.06493400
H	0.57172400	7.94835000	-2.11652900
H	0.05256200	6.25208400	-1.91818600
H	3.41046900	8.50975400	1.39769700
H	7.01854800	5.67903800	1.22028300
H	13.74332200	3.27652000	-3.20974400
H	-0.41438200	-1.05205500	-8.77631100
H	-1.18652000	-2.60876300	-8.44032100
H	8.59216600	-6.00361700	0.92821600
H	-0.80324000	-7.42396700	-2.04302500
H	7.23007100	0.52461800	4.29899100
H	3.71542300	1.55858200	5.16661200
H	-8.46706800	0.55182300	2.00482300
H	8.12457700	2.95667000	-1.66977500

References

[1] S. A. Marshall, K. Fisher, A. Ni Cheallaigh, M. D. White, K. A. Payne, D. A. Parker, S. E. Rigby, D. Leys, *J. Biol. Chem.* **2017**, *292*, 4623-4637.
[2] C.-R. Liao, Y.-H. Kuo, Y.-L. Ho, C.-Y. Wang, C. Yang, C.-W. Lin, Y.-S. Chang, *Molecules* **2014**, *19*, 9515.
[3] A. Burkhardt, T. Pakendorf, B. Reime, J. Meyer, P. Fischer, N. Stübe, S. Panneerselvam, O. Lorbeer, K. Stachnik, M. Warmer, P. Rödig, D. Göries, A. Meents, *Eur. Phys. J. Plus* **2016**, *131*, 56.
[4] a) D. Nurizzo, T. Mairs, M. Guijarro, V. Rey, J. Meyer, P. Fajardo, J. Chavanne, J. C. Biasci, S. McSweeney, E. Mitchell, *J. Synchrotron Radiat.* **2006**, *13*, 227-238; b) P. Theveneau, R. Baker, R. Barrett, A. Beteva, M. W. Bowler, P. Carpentier, H. Caserotto, D. de Sanctis, F. Dobias, D. Flot, M. Guijarro, T. Giraud, M. Lentini, G. A. Leonard, M. Mattenet, A. A. McCarthy, S. M. McSweeney, C. Morawe, M. Nanao, D. Nurizzo, S. Ohlsson, P. Pernot, A. N. Popov, A. Round, A. Royant, W. Schmid, A. Snigirev, J. Surr, C. Mueller-Dieckmann, *J. Phys.: Conf. Ser.* **2013**, *425*, 012001; c) D. Flot, T. Mairs, T. Giraud, M. Guijarro, M. Lesourd, V. Rey, D. van Brussel, C. Morawe, C. Borel, O. Hignette, J. Chavanne, D. Nurizzo, S. McSweeney, E. Mitchell, *J. Synchrotron Radiat.* **2010**, *17*, 107-118.
[5] W. Kabsch, *Acta Crystallogr., Sect. D: Biol. Crystallogr.* **2010**, *66*, 125-132.
[6] P. D. Adams, P. V. Afonine, G. Bunkoczi, V. B. Chen, I. W. Davis, N. Echols, J. J. Headd, L. W. Hung, G. J. Kapral, R. W. Grosse-Kunstleve, A. J. McCoy, N. W. Moriarty, R. Oeffner, R. J. Read, D. C. Richardson, J. S. Richardson, T. C. Terwilliger, P. H. Zwart, *Acta Crystallogr., Sect. D: Biol. Crystallogr.* **2010**, *66*, 213-221.
[7] T. C. Terwilliger, R. W. Grosse-Kunstleve, P. V. Afonine, N. W. Moriarty, P. H. Zwart, L. W. Hung, R. J. Read, P. D. Adams, *Acta Crystallogr., Sect. D: Biol. Crystallogr.* **2008**, *64*, 61-69.
[8] P. Emsley, B. Lohkamp, W. G. Scott, K. Cowtan, *Acta Crystallogr., Sect. D: Biol. Crystallogr.* **2010**, *66*, 486-501.
[9] A. J. McCoy, R. W. Grosse-Kunstleve, P. D. Adams, M. D. Winn, L. C. Storoni, R. J. Read, *J. Appl. Crystallogr.* **2007**, *40*, 658-674.
[10] G. J. Kleywegt, A. T. Brunger, *Structure* **1996**, *4*, 897-904.
[11] V. B. Chen, W. B. Arendall, 3rd, J. J. Headd, D. A. Keedy, R. M. Immormino, G. J. Kapral, L. W. Murray, J. S. Richardson, D. C. Richardson, *Acta Crystallogr., Sect. D: Biol. Crystallogr.* **2010**, *66*, 12-21.
[12] P. Di Tommaso, S. Moretti, I. Xenarios, M. Orobitg, A. Montanyola, J. M. Chang, J. F. Taly, C. Notredame, *Nucleic Acids Res.* **2011**, *39*, W13-17.
[13] X. Robert, P. Gouet, *Nucleic Acids Res.* **2014**, *42*, W320-324.
[14] L. Holm, P. Rosenstrom, *Nucleic Acids Res.* **2010**, *38*, W545-549.
[15] E. Krissinel, K. Henrick, *Acta Crystallogr., Sect. D: Biol. Crystallogr.* **2004**, *60*, 2256-2268.
[16] E. Krissinel, K. Henrick, *J. Mol. Biol.* **2007**, *372*, 774-797.
[17] J. Vonck, D. N. Parcej, D. J. Mills, *PLoS One* **2016**, *11*, e0159476.
[18] S. Q. Zheng, E. Palovcak, J.-P. Armache, K. A. Verba, Y. Cheng, D. A. Agard, *Nat. Meth.* **2017**, *14*, 331-332.
[19] S. H. W. Scheres, *J. Struct. Biol.* **2012**, *180*, 519-530.
[20] K. Zhang, *J. Struct. Biol.* **2016**, *193*, 1-12.
[21] S. H. W. Scheres, *Elife* **2014**, *3*, e03665.
[22] S. Chen, G. McMullan, A. R. Faruqi, G. N. Murshudov, J. M. Short, S. H. W. Scheres, R. Henderson, *Ultramicroscopy* **2013**, *135*, 24-35.
[23] E. F. Pettersen, T. D. Goddard, C. C. Huang, G. S. Couch, D. M. Greenblatt, E. C. Meng, T. E. Ferrin, *J Comput Chem* **2004**, *25*, 1605-1612.
[24] M. J. Frisch, G. W. Trucks, H. B. Schlegel, G. E. Scuseria, M. A. Robb, J. R. Cheeseman, G. Scalmani, V. Barone, B. Mennucci, G. A. Petersson, H. Nakatsuji, M. Caricato, X. Li, H. P. Hratchian, A. F. Izmaylov, J. Bloino, G. Zheng, J. L. Sonnenberg, M. Hada, M. Ehara, K. Toyota, R. Fukuda, J. Hasegawa, M. Ishida, T. Nakajima, Y. Honda, O. Kitao, H. Nakai, T. Vreven, J. A. Montgomery, Jr., J. E. Peralta, F. Ogliaro, M. Bearpark, J. J. Heyd, E. Brothers, K. N. Kudin, V. N. Staroverov, T. Keith, R. Kobayashi, J. Normand, K. Raghavachari, A. Rendell, J. C. Burant, S. S. Iyengar, J. Tomasi, M. Cossi, N. Rega, J. M. Millam, M. Klene, J. E. Knox, J. B. Cross, V. Bakken, C. Adamo, J. Jaramillo, R. Gomperts, R. E. Stratmann, O. Yazyev, A. J. Austin, R. Cammi, C. Pomelli, J. W. Ochterski, R. L. Martin, K. Morokuma, V. G. Zakrzewski, G. A. Voth, P. Salvador, J. J. Dannenberg, S. Dapprich, A. D. Daniels, O. Farkas, J. B. Foresman, J. V. Ortiz, J. Cioslowski, D. J. Fox, in *Gaussian, Inc.: Wallingford, Vol. CT*, **2013**.
[25] a) A. D. Becke, *J. Chem. Phys.* **1993**, *98*, 5648-5652; b) C. Lee, W. Yang, R. G. Parr, *Phys. Rev. B: Condens. Matter Mater. Phys.* **1988**, *37*, 785-789.
[26] A. V. Marenich, C. J. Cramer, D. G. Truhlar, *J. Phys. Chem. B* **2009**, *113*, 6378-6396.
[27] a) S. Grimme, J. Antony, S. Ehrlich, H. Krieg, *J. Chem. Phys.* **2010**, *132*, 154104; b) S. Grimme, S. Ehrlich, L. Goerigk, *J. Comp. Chem.* **2011**, *32*, 1456-1465.
[28] a) X. Sheng, M. E. Lind, F. Himo, *FEBS J.* **2015**, *282*, 4703-4713; b) M. E. S. Lind, F. Himo, *ACS Catal.* **2014**, *4*, 4153-4160.
[29] A. Jacewicz, A. Izumi, K. Brunner, R. Schnell, G. Schneider, *PLoS One* **2013**, *8*, e63161.
[30] K. A. Payne, M. D. White, K. Fisher, B. Khara, S. S. Bailey, D. Parker, N. J. Rattray, D. K. Trivedi, R. Goodacre, R. Beveridge, P. Barran, S. E. Rigby, N. S. Scrutton, S. Hay, D. Leys, *Nature* **2015**, *522*, 497-501.
[31] a) K. L. Ferguson, N. Arunrattanamook, E. N. Marsh, *Biochemistry* **2016**, *55*, 2857-2863; b) G. Tian, Y. Liu, *Phys. Chem. Chem. Phys.* **2017**, *19*, 7733-7742.
[32] a) A. Piano, B. A. Palfey, A. Mattevi, *Trends Biochem. Sci.* **2017**, *42*, 457-469; b) C. L. Lan, S. L. Chen, *J. Org. Chem.* **2016**, *81*, 9289-9295; c) M. Baunach, C. Hertweck, *Angew. Chem. Int. Ed.* **2015**, *54*, 12550-12552.
[33] K. L. Ferguson, J. D. Eschweiler, B. T. Ruotolo, E. N. G. Marsh, *J. Am. Chem. Soc*. doi: 10.1021/jacs.7b0506.

Author Contributions

S. E. Payer: preparation of reconstituted enzymes for crystallization and Cryo-EM; whole-cell biotransformations including mutagenesis and heterologous expression of variants; conversion analytics and structural characterization; writing of original draft (lead, equal)

S. A. Marshall: in vitro reconstitution for, and measuring of, kinetics, spectral and crystallization data; crystallization of proteins and processing of structural data; writing of original draft (lead, equal)

N. Bärland: performed cryo-EM experiments and data processing (lead)

X. Sheng: density functional calculations; writing of original draft (lead)

T. Reiter: contributed to preliminary results and preparation of *Ec*AroY variants (supporting)

A. Dordic: contributed to protein crystallization and structure refinement (supporting)

G. Steinkellner: suggested mutation sites (supporting)

C. Wuensch: contributed to preliminary results (supporting)

S. Kaltwasser: involved in cryo-EM experiments (supporting)

K. Fisher: performed and analyzed EPR experiments (supporting)

S. E. J. Rigby: analyzed EPR data and wrote corresponding sections (supporting)

P. Macheroux: discussion concerning the design of experiments (supporting)

J. Vonck: supervised the cryo-EM experiments, contributed to original draft writing (supporting)

K. Gruber: involved in discussion, acquired financial funding for this research (supporting)

K. Faber: involved in discussion, involved in the mechanistic proposal, acquirement of financial funding (lead)

F. Himo: density functional calculations, involved in discussion, preparing of the original draft (lead)

D. Leys: experimental design, preparation of the original draft, acquirement of financial funding (lead)

T. Pavkov-Keller: design, performance and analysis of X-ray and cryo-EM experiments, validation, preparation of the original draft (lead)

S. M. Glueck: involved in preliminary literature research, experimental design, preparation of the original draft, acquired financial funding for this research (lead)
